# Flavonoids as Nutraceuticals to Treat Inflammatory Diseases: Focusing on Quercetin, Kaempferol, Luteolin, Apigenin, Epicatechin and Their Effects on Hepatic, Nervous, and Pulmonary Systems

**DOI:** 10.3390/foods15122159

**Published:** 2026-06-15

**Authors:** Maiara Piva, Geovana Martelossi-Cebinelli, Soraia Mendes-Pierotti, Willian H. Chinen, Pedro H. F. Cardines, Renata M. Martinez, Sandra R. Georgetti, Marcela M. Baracat, Fabiana T. M. C. Vicentini, Waldiceu A. Verri, Rubia Casagrande

**Affiliations:** 1 Laboratory of Pain, Inflammation, Neuropathy and Cancer, Department of Immunology, Parasitology, and General Pathology, Center of Biological Sciences, Londrina State University, Londrina 86057-970, PR, Brazil; 2 Department of Pharmaceutical Sciences, Center of Health Science, Londrina State University, Londrina 86038-440, PR, Brazil; 3 Department of Biochemistry and Biotechnology, Center of Exact Sciences, Londrina State University, Londrina 86057-970, PR, Brazil; 4 School of Pharmaceutical Sciences of Ribeirão Preto, University of São Paulo, Ribeirão Preto 14048-900, SP, Brazil; fabtesta@fcfrp.usp.br

**Keywords:** flavonoids, inflammation, inflammatory diseases, nutraceutical

## Abstract

The immune response is essential in the protection of our body against pathogens; however, the inflammatory response caused by the immune system can become a disease itself. In fact, anti-inflammatory and immune-suppressive drugs are applied to limit the immune response to treat inflammatory diseases. Flavonoids are plant-derived polyphenols extensively investigated for their anti-inflammatory and antioxidant properties in inflammatory diseases. Studies applying isolated compounds as well as using supplements as nutraceuticals based on flavonoids have been conducted. Our review systematically analyzed the top five studied flavonoids between 2020 and 2025: quercetin (1742 articles), kaempferol (642), luteolin (589), apigenin (419), and epicatechin (354), highlighting their major therapeutic applications in diseases affecting the liver (12%), nervous system (11%), and lungs (10%). Mechanistically, these compounds act as multi-target agents mainly by inhibiting NF-κB and inducing Nrf2-dependent antioxidant programs. Application of advanced delivery systems, which increase oral bioavailability by up to 20-fold, overcomes pharmacokinetic bottlenecks. Clinical highlights demonstrated promising therapeutic effects, including reduced intrahepatic lipid accumulation in non-alcoholic fatty liver disease patients following quercetin supplementation (11.5% to 9.6%) and accelerated SARS-CoV-2 clearance after quercetin phytosome administration. The translation of flavonoids into standardized clinical therapies remains limited by the lack of large-scale, well-controlled clinical trials.

## 1. Introduction

**Figure 1:**
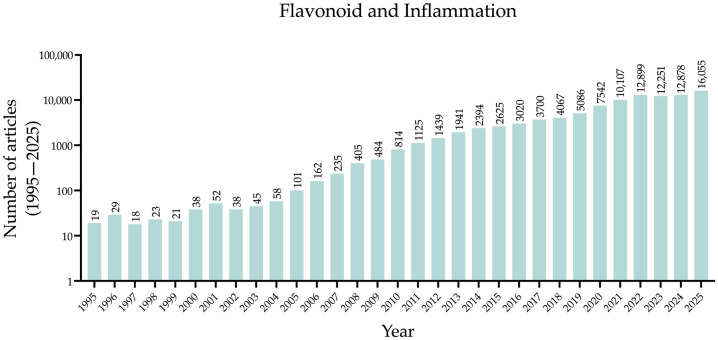
Number of articles on flavonoids and inflammation indexed in PubMed between January 1995 and December 2025. Source: PubMed database (National Center for Biotechnology Information—NCBI). Created in GraphPad Prism version 10.0.0 for Mac OS X, GraphPad Software, Boston, Massachusetts USA, www.graphpad.com.

Plant-based food sources and their derivatives are more than ever the aim of scientific research due to their medicinal and health-improving properties. Among these natural products, flavonoids are notorious antioxidants that form a family of plant-derived secondary metabolites and share a basic chemical structure called the flavan nucleus (composed of two benzene rings linked by a heterocyclic pyran ring) [1]. Scientific interest in flavonoids’ bioactive properties has been increasingly rising over the past three decades, accounting for almost 15,000 articles on inflammation (Figure 1). These studies have primarily evaluated the safety profile and protective effects of flavonoids against oxidative damage in non-cancerous settings, preventing and mitigating inflammatory and metabolic conditions [2].

It is believed that flavonoids were first described by the 1937 Nobel Prize laureate, the Hungarian biochemist Albert Szent-Gyorgyi, referring as “vitamin P” to the flavonoid nowadays known as rutin—a variation of the most studied flavonoid currently, quercetin. In rutin, the hydroxy group at the C-3 position of quercetin is substituted with glucose and rhamnose groups [3,4].

These compounds are widely present in plants and are included in the human and animal diet [1], being found mainly in the vacuoles of plant cells in the form of C-glycosides or O-glycosides, acting as attractants to pollinators and symbionts [e.g., sunscreens against ultraviolet (UV) radiation, allelochemicals, and antimicrobial and antiherbivore factors] [5,6].

Among the classes, isoflavones (present in soybean), flavonols (present in teas, onions, red wine, olive oil, among others), and flavones (present in fruit skins, buckwheat, red pepper, among others) are the flavonoids found in the greatest quantity in foods consumed by humans [1]. However, the amount ingested varies according to the food source, processing, and preparation of food, and there may be a reduction or redistribution of flavonoids [1]. Even so, their antioxidant and anti-inflammatory properties have placed flavonoids as important components in nutraceutical, pharmaceutical, medicinal and cosmetic applications [7].

The benefits of flavonoid-rich foods consumption are shown against diseases that reduce lifespan due to their protective role against oxidative damage, a condition associated with many non-communicable diseases such as cardiovascular diseases, diabetes, cancer, and dementia [2,8]. Among their advantages, the low toxicity profile of flavonoids and wide dietary availability favor the incorporation into preventive and complementary therapeutic strategies, whether through functional foods or pharmaceutical formulations. Preclinical and clinical studies have been advancing to better elucidate the pharmacokinetics, bioavailability and molecular mechanisms involved in their effects [9,10,11].

The role of flavonoids as nutraceuticals also involves their impact on the intestinal microbiome through promoting the growth of commensal bacteria and the production of metabolites (e.g., short-chain fatty acids). This interaction enhances the systemic effects of flavonoids and indicates the importance of an integrated approach in the treatment of chronic inflammatory conditions [12].

Inflammatory diseases pose a significant challenge to global public health. They are among the main causes of disability and account for a large share of global deaths and healthcare expenses [13]. The World Health Organization (WHO) states that non-communicable diseases lead to nearly 74% of deaths globally. Chronic inflammation is part of the physiopathology of some non-communicable diseases, including chronic respiratory diseases, metabolic diseases, cardiovascular diseases, autoimmune diseases, rheumatic diseases, cancers, chronic kidney disease, obesity, and neurodegenerative diseases [14], thus highlighting the urgent need for effective prevention and treatment strategies. Persistent inflammation is closely linked to oxidative stress because reactive oxygen species (ROS) can trigger intracellular signaling pathways leading to inflammation [14]. Excessive production of ROS and reactive nitrogen species (RNS) activates pathways such as NF-κB and MAPKs. These pathways contribute to tissue damage and increase the production of pro-inflammatory cytokines. In this context, flavonoids have garnered attention from scientists due to their antioxidant, anti-inflammatory, and immune-modulatory properties. They show promise as beneficial compounds for preventing and treating inflammation-related diseases [15,16].

In general, inflammatory conditions demonstrate an excessive release of ROS and RNS, culminating in the development of oxidative stress since endogenous antioxidants are not sufficient to limit the activity of ROS and RNS, including the deleterious effects such as cellular damage [17]. This condition promotes the development of nociceptive responses (pain), an increase in pro-inflammatory mediators, and the activation of transcription pathways important for the establishment of inflammation. In turn, inflammation also produces oxidative stress. For instance, phagocytes, including macrophages, produce ROS and RNS via enzymes such as nicotinamide adenine dinucleotide phosphate oxidase (NADPH) oxidase and inducible nitric oxide synthase (iNOS). This intimate relation between oxidative stress and inflammation explains why it is seen as a feedback-fueling relation. All these effects can be reversed through the anti-inflammatory action of flavonoids, which were approached in more detail in Section 4.

In this context, this review summarizes the effects of the following flavonoids: quercetin, kaempferol, luteolin, apigenin, and epicatechin, in the prevention or treatment of important inflammatory diseases, highlighting their mechanisms of action, participation in signaling pathways, and current clinical data. These flavonoids were selected because they were the top five most studied compounds from this class between 2020 and 2025.

A comparative analysis of their molecular mechanisms, structure–activity relationships, pharmacokinetic limitations, and therapeutic relevance across hepatic, nervous, and pulmonary inflammatory diseases was performed. A major distinguishing feature is the selective inclusion of in vivo and clinical evidence rather than predominantly in vitro or in silico studies, allowing a more clinically meaningful interpretation of flavonoid efficacy. Furthermore, this review highlights emerging bioavailability-enhancing strategies, including phytosomes and nanoformulations, as critical advances overcoming historical translational barriers, while also correlating mechanistic pathways such as NF-κB suppression and Nrf2 activation with functional and histopathological outcomes across disease models.

## 2. Methodology

**Figure 2:**
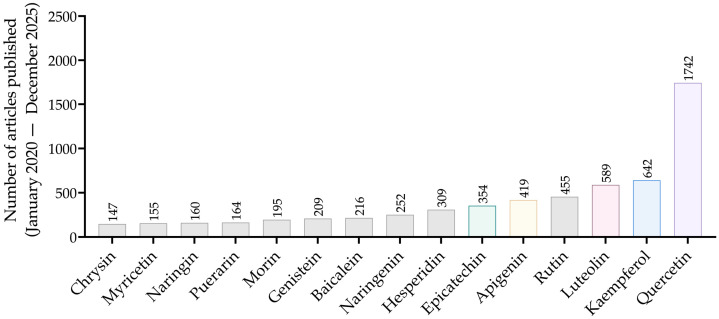
Number of research articles (excluding reviews) related to flavonoids and inflammation, indexed in PubMed from January 2020 to December 2025. Source: PubMed database (National Center for Biotechnology Information—NCBI). Bars representing the selected flavonoids are colored while other flavonoids are represented by gray bars. As mentioned in the methodology, despite the high number of studies on rutin, this flavonoid was not selected for this review, considering that quercetin has a higher number of studies and is the aglycone form of rutin. Created in GraphPad Prism version 10.0.0 for Mac OS X, GraphPad Software, Boston, Massachusetts USA, www.graphpad.com.

**Figure 3:**
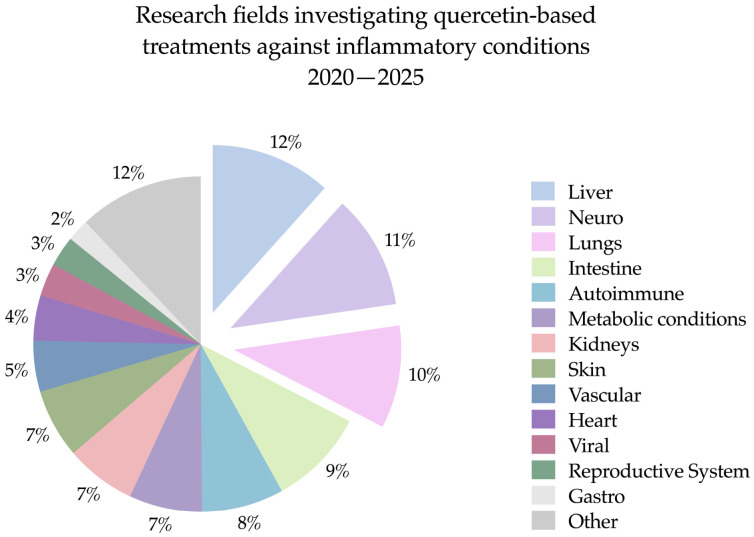
Main research areas investigating the use of quercetin- or quercetin-present extracts in inflammatory conditions (excluding reviews) between January 2020 and December 2025 and indexed in PubMed. Source: PubMed database (National Center for Biotechnology Information—NCBI), search conducted in December 2025. The top three research fields were selected to be approached in this review. Created in GraphPad Prism version 10.0.0 for Mac OS X, GraphPad Software, Boston, Massachusetts USA, www.graphpad.com.

This narrative review summarizes the main findings on flavonoids and flavonoid-containing nutraceuticals to improve inflammation-related conditions published between 2020 and 2025. A search of the PubMed database was initially conducted to identify the most studied flavonoids in inflammatory settings during the aforementioned timeframe, excluding reviews—search parameters “flavonoids” AND “inflammation” NOT “review” from January 1995 to December 2025 (Figure 2). Quercetin was the main flavonoid studied to improve inflammatory conditions, with over 1500 results in six years. Rutin was also among the most studied flavonoids. However, as quercetin is an aglycone form of rutin, only the data from quercetin were discussed. The next stage of our data recovery was to determine the main areas in which quercetin was employed as a treatment or dietary supplement for inflammatory conditions. The search parameters were “quercetin” AND “inflammation” NOT “review” from January 2020 to December 2025. The timeframe of six years was established to focus the current review on the latest research published, whereas previous studies have been reviewed by Ferraz et al. (2020) [18]. Scientific papers with in vivo experimentation and/or clinical trials were screened based on title and abstract content, and sorted according to the field of investigation, which indicated that the liver, the nervous system and lungs were the preferential targets to investigate quercetin effects (Figure 3).

These fields were extrapolated to the five most studied flavonoids in this period, namely quercetin (1742 studies), kaempferol (642 studies), luteolin (589 studies), apigenin (419 studies), and epicatechin (354 studies), as well as the three most investigated areas (liver—12%, nervous system—11%, and lungs—10%). Research articles based solely on in silico predictions and/or in vitro findings were not included in the present literature review, which was focused on in vivo findings and human clinical trials selected into each described category. The data search was performed independently by three of the authors.

## 3. General Flavonoid Structure, Dietary Sources, Structure–Activity Relationships, and Molecular Mechanisms of Action

Currently, there are discrepancies in the literature regarding the number of flavonoids already described, with estimates ranging from approximately 6000 [19], 9000 [20], to even more than 10,000 known [5].

**Figure 4:**
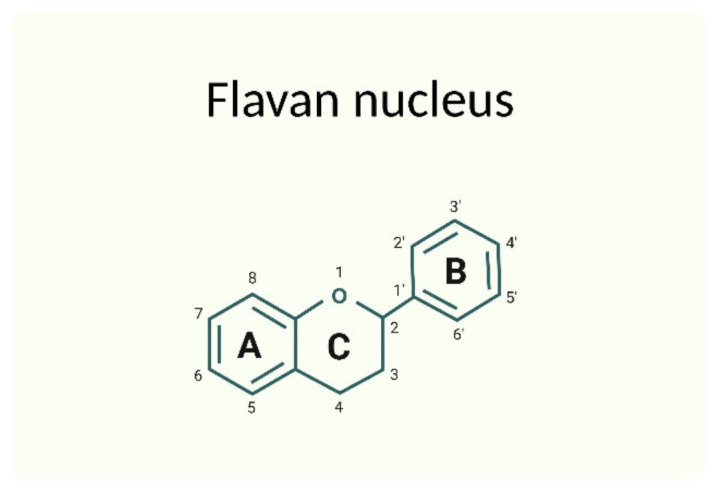
2D chemical structure of the flavan nucleus, the common structure shared by flavonoids. Numbers indicate the postion of carbon atoms. Created in BioRender. Verri Jr, W. A. (2026) https://BioRender.com/krbxie3 (accessed on 5 June 2026).

Flavonoids are natural sources of antioxidants synthesized in plants through the phenylpropanoid metabolic pathway, being regulated by multiple genes and enzymes that respond to environmental and physiological stimuli in plants [6]. These compounds are structurally based on the flavan nucleus, a basic structure composed of 15 carbon atoms arranged in three rings (C6–C3–C6) (Figure 4) [6].

**Figure 5:**
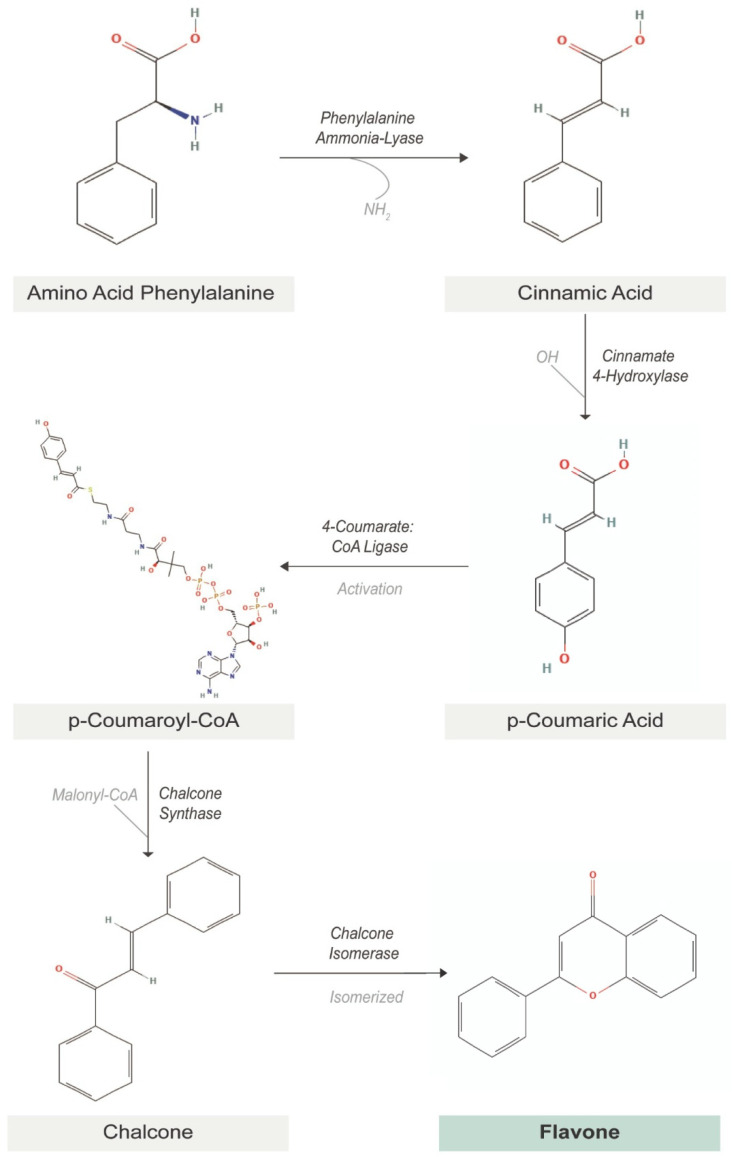
Schematic representation of flavonoid biosynthesis. Flavonoid biosynthesis begins with the conversion of phenylalanine into cinnamic acid by phenylalanine ammonia-lyase, followed by the formation of p-coumaroyl–CoA through the sequential action of cinnamate 4-hydroxylase and 4-coumarate:CoA ligase. Chalcone synthase then catalyzes the condensation of p-coumaroyl–CoA with malonyl–CoA to generate naringenin chalcone, the central precursor of flavonoids. Finally, chalcone isomerase converts this intermediate into flavone, which serves as the main branching point for the formation of the different flavonoid classes. Image of 2D structures of amino acid phenylalanine (CID 6140), cinnamic acid (CID 444539), p-coumaric acid (CID 637542), p-coumaroyl–CoA (CID 6440013), chalcone (CID 637760), and flavone (CID 10680) retrieved from PubChem. National Center for Biotechnology Information. https://pubchem.ncbi.nlm.nih.gov/ (accessed 23 April 2026). Created in BioRender. Verri Jr, W. A. (2026) https://BioRender.com/krbxie3 (accessed on 5 June 2026).

Flavonoid biosynthesis (Figure 5) begins with the conversion of the amino acid phenylalanine into cinnamic acid through the action of the enzyme phenylalanine ammonia-lyase (PAL), which is responsible for removing the amine group from this molecule. Subsequently, the enzyme cinnamate 4-hydroxylase (C4H) adds a hydroxyl group to cinnamic acid, forming p-coumaric acid, and the enzyme 4-coumarate: CoA ligase (4CL) promotes the activation of this acid to produce p-coumaroyl–CoA. Next, chalcone synthase (CHS) catalyzes the condensation of one molecule of p-coumaroyl–CoA with three molecules of malonyl–CoA, producing the key intermediate for all flavonoids, called chalcone (naringenin chalcone). Chalcone is subsequently isomerized into flavone via chalcone isomerase (CHI), reaching the central branching point for the different classes of flavonoids [5].

Based on structure, oxidation state and saturation of the heterocyclic ring, flavonoids can be classified into different classes. The two main groups are the 2-phenylchromans flavonoids, also known as classic flavonoids (flavanones, flavones, flavonols, flavan-3-ols, and anthocyanidins), and the 3-phenylchromans, known as isoflavonoids (isoflavones, isoflavans, and pterocarpans) [5]. Plants’ colors, fragrance, and flavor characteristics are some of the features that can be attributed to flavonoids’ secondary metabolites. Moreover, they can be involved in allelopathic processes, such as cell growth regulation, male fertility, seed development, protection against biotic and abiotic stresses, auxin transport regulation, and pollinator insects’ attraction [21,22,23].

### 3.1. Structure–Activity Relationships of Flavonoids in Redox and Inflammatory Modulation

Studies demonstrate that the specific structural characteristics of flavonoids are highly associated with their biological and pharmacological activities; that is, the position and number of substituents in the basic structure modulate the antioxidant and anti-inflammatory activities of these molecules [24,25,26]. For instance, the double bond between C2 and C3 in ring A, as well as the presence of hydroxyl groups at C3′ and C4′, and a carbonyl group at the C4 position, have been shown to increase the hepatoprotective activities of bioactive flavonoids. Conversely, hydroxymethylation at C3′ and C4′ has shown the opposite effect, decreasing hepatoprotective activity [27].

In addition to enzymatic modulations, flavonoids exert antioxidant effects. Peroxynitrite (ONOO^−^) is a cytotoxic intermediate produced by the reaction between a superoxide anion radical and nitric oxide (NO) [28]. Choi et al. (2002) evaluated the ability of a series of structurally related flavonoids to neutralize the ONOO^−^ and how structural differences influence this activity [28]. As a result, it was observed that ONOO^−^ was neutralized by the presence of several flavonoids, whose structures appear to be closely related to the degree of neutralization. It was postulated that the neutralizing activity is governed by the position of the hydroxyl group (OH), where O-hydroxyl structures increased the ONOO^−^ scavenging activity. Of the 31 compounds tested, the most active was quercetin (IC_50_ of 0.93 µM) [28].

Zhang et al. (2020) investigated the antioxidant activity of 60 flavonoids with different structural patterns through three assays: 2,2-diphenyl-1-picrylhydrazyl (DPPH) radical scavenging activity, oxygen radical absorbance capacity, and cellular antioxidant activity [29]. As a result, they identified six flavonoids with significant intracellular antioxidant activity, correlated with an up-regulation of antioxidant enzymes such as superoxide dismutase (SOD), catalase (CAT), and glutathione peroxidase (GPx) [29]. Through structure–activity relationship analysis, it was observed that the presence of a double bond between C2 and C3 in ring C, a ketone group at C4, a catechol structure in ring B, and the presence of a hydroxyl group at C3 favor cellular antioxidant activity [29].

From a pharmacological point of view, different flavonoids have been shown to exhibit antioxidant, anti-inflammatory, analgesic, antimicrobial, and immunomodulatory activities. These actions are attributed to their ability to modulate oxidative stress and regulate signaling pathways, such as nuclear factor kappa B (NF-κB), mitogen-activated protein kinase (MAPK), arachidonic acid (AA) pathway, phosphatidylinositide 3-kinases/protein kinase B (PI3K/Akt) and mammalian target of rapamycin complex 1 (mTORC1) signaling pathways, and by inflammatory mediators inhibition, like interleukin-1 beta (IL-1β), tumor nuclear factor-alpha (TNF-α), prostaglandin E_2_ (PGE_2_) and NO (Topic 4, Box 1 and Box 2) [30,31].

**Figure 6:**
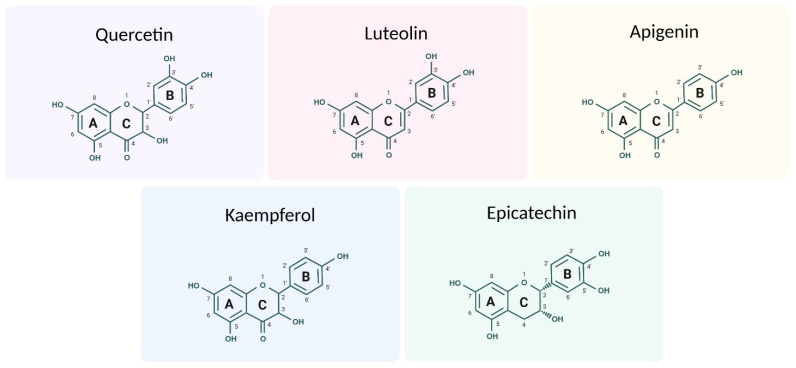
2D chemical structure of quercetin, luteolin, apigenin, kaempferol, and epicatechin. Numbers indicate the postion of carbon atoms. Created in BioRender. Verri Jr, W. A. (2026) https://BioRender.com/krbxie3 (accessed on 5 June 2026).

This section discusses the structural characteristics of the five selected flavonoids: quercetin, kaempferol, luteolin, apigenin, and epicatechin (Figure 6), their main dietary sources, and how structure–activity relationships determine their redox properties and the molecular mechanisms relevant to inflammation.

#### 3.1.1. Quercetin

Quercetin (2-(3,4-dihydroxyphenyl)-3,5,7-trihydroxy-4H-chromen-4-one) is considered a model flavonoid belonging to the flavonol class [32], with remarkable antioxidant properties [33]. Its chemical structure consists of five OH groups at positions 3, 5, 7, 3′, and 4′ of the basic skeleton of flavonol (Figure 6). For the formation of its derivatives (e.g., isoquercetin, quercetin 3-O-galactoside, quercetin 3-O-rhamnoside, quercetin 7-O-rhamnoside, and rutin), quercetin can present different glycosylation in its OH groups [32].

It is found widely distributed in fruits and vegetables, such as onions [34,35], berries, capers, coriander, and apples [36]. A dietary intake of 100 g onion is equivalent to approximately 35–120 mg of quercetin, and a dietary intake of 200 g of curly kale is equivalent to approximately 60–120 mg of quercetin [34]. Quercetin has low oral bioavailability due to its lipophilicity and metabolism in the gastrointestinal tract. After absorption, it accumulates in tissues, especially in the lungs, liver, kidneys, and intestines [37].

Among its therapeutic properties, studies show that quercetin is used in the treatment of cancer, allergic reactions, inflammation, arthritis, and cardiovascular disorders [38]. Furthermore, it plays an important role in platelet aggregation, acts as a reducing agent for coagulation, hyperglycemia, inflammation, and hypertension [38,39], and contributes to the elimination of free radicals [33,38]. Taken together, studies demonstrate that quercetin downregulates the glycolytic activity of macrophages and induces the anti-inflammatory reprogramming of the tricarboxylic acid (TCA) cycle [31].

**Figure 7:**
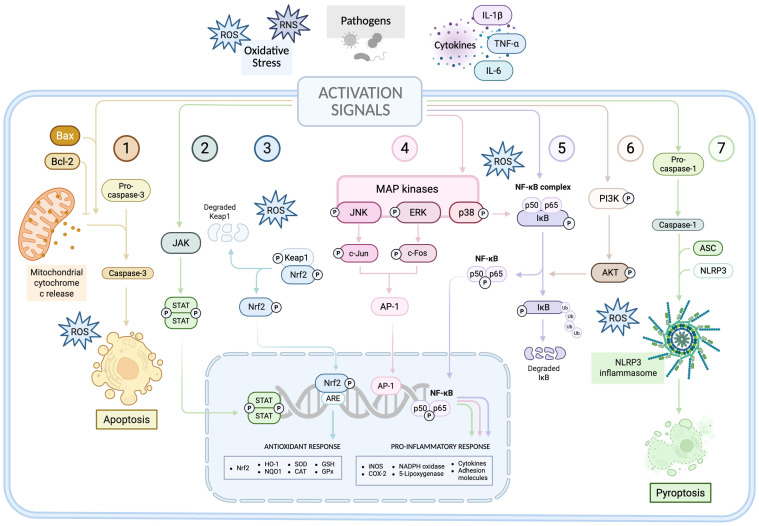
Summary of the multi-target mechanisms of flavonoids in inflammatory diseases. Evidence supports that, depending on the disease-specific mechanisms, flavonoids can inhibit multiple targets. The most consistent targets of quercetin, luteolin, apigenin, kaempferol, and epicatechin are shown in this figure. Flavonoids can inhibit apoptosis (1) and JAK/STAT signaling (2) while enhancing Nrf2 signaling (3), which is also stimulated by inflammatory and oxidative stress responses as a compensatory/regulatory mechanism. The activation of the Nrf2 signaling leads to up-regulation of molecules with a role in the endogenous antioxidant response. MAPK (4) can lead to AP-1 and NF-κB activation (5), and PI3K can also cause NF-κB activation (6), which are also targeted by flavonoids. The AP-1 and NF-κB pathways lead to the expression of pro-inflammatory enzymes and molecules. NLRP3 inflammasome and pyroptosis are also targeted by flavonoids (7). Created in BioRender. Verri Jr, W. A. (2026) https://BioRender.com/krbxie3 (accessed on 5 June 2026).

Regarding its antioxidant properties, studies demonstrate that quercetin acts by directly neutralizing reactive oxygen species (ROS) through electron donation, increasing the activity of endogenous antioxidant enzymes [40], and stimulating antioxidant pathways (e.g., nuclear factor erythroid 2-related factor 2—Nrf2) [41], in addition to reducing lipid peroxidation [42]. Regarding its anti-inflammatory properties, studies show that quercetin inhibits the activation and deoxyribonucleic acid (DNA) binding of the transcription factor NF-κB [43] and, consequently, reduces the expression of pro-inflammatory cytokines (e.g., TNF-α, IL-1β, and interleukin 6—IL-6) [42]. Another important mechanism of action is the targeting of MAPKs, such as extracellular signal-regulated kinases (ERK) and c-Jun amino-terminal kinases (JNK) [42] (Figure 7).

#### 3.1.2. Kaempferol

Kaempferol (3,5,7-trihydroxy-2-(4-hydroxyphenyl)-4H-1-benzopyran-4-one) is a natural compound with antioxidant and anti-inflammatory properties belonging to the flavonol class [44]. The structure of kaempferol consists of a flavone skeleton with three OH groups at positions 3, 5, and 7, and a double bond between carbons 2 and 3 (Figure 6). The structure–activity relationship studies on kaempferol suggest that the 2,3-double bond, the 4-keto group, the 3,4-catechol structure, and the 3-hydroxyl group are the key determinants of its biological activity [44].

Kaempferol is commonly found in a wide range of foods, beverages, and plants such as beans, spinach, tea, kale, and broccoli. It has a lipophilic nature, being absorbed by facilitated diffusion, passive diffusion, and active transport [44,45]. An intake of 14.97 mg per day of kaempferol results in a plasma concentration of 57.86 nM, and the consumption of 27 mg of tea containing this flavonoid results in a plasma concentration of 15 ng/mL [44,45].

Regarding its mechanisms of action to control inflammation, studies demonstrate that kaempferol inhibits the production of pro-inflammatory cytokines (e.g., TNF-α, IL-1β, and IL-6) and reduces the production of other mediators such as cyclooxygenase-2 (COX-2) and inducible nitric oxide synthase (iNOS) [44]. Furthermore, this flavonoid is known to modulate signaling pathways important for the development and perpetuation of inflammation, such as the NF-κB, MAPKs, and signal transducer and activator of transcription (STAT) protein pathways [44]. Regarding antioxidant mechanisms, studies demonstrate that kaempferol stimulates antioxidant pathways (e.g., Nrf2), increasing the expression of antioxidant enzymes and protecting against ROS [44] (Figure 7).

Furthermore, experimental studies have demonstrated that kaempferol and its nanoformulations modulate signaling pathways associated with oxidative stress, apoptosis, and inflammation, resulting in functional recovery and tissue preservation in various models of neuronal damage and toxicity [44,46].

#### 3.1.3. Luteolin

Luteolin (2-(3,4-dihydroxyphenyl)-5,7-dihydroxychromen-4-one) is a natural flavonoid with antioxidant, anti-inflammatory, apoptosis-inducing, and chemopreventive activity. Its structure consists of four OH groups located at positions 3′, 4′, 5, and 7; a double bond between C2 and C3; and a carbonyl group at C4 (Figure 6) [47]. This flavonoid can be found in vegetables, fruits, herba ajugae, artichoke, callicarpa nudiflora, and hemp seeds [48,49].

The B-cyclic catechol structure of luteolin can contribute hydrogen electrons (H^+^), stabilize free radicals, thereby clearing harmful free radicals, protecting human cells or tissues, and delaying human aging [47,50]. To further enhance the biological activity of luteolin, researchers have carried out various structural adjustments and modifications. In recent years, the research focus has mainly been on structural modifications such as alkylation, acylation, and salt formation on its phenolic OH groups [47,51].

Luteolin exhibits a high inhibitory activity against the synthesis of AA metabolites such as thromboxane and leukotriene. Luteolin structure also allows scavenging ROS by donating hydrogen electrons. In various models of neuroinflammation and injury, luteolin consistently counteracts oxidative stress and inflammatory cascades through suppression of pro-inflammatory mediators such as IL-1β, IL-6, TNF-α, and NF-κB, while enhancing endogenous antioxidant defenses (e.g., SOD, CAT, glutathione—GSH, Nrf2/heme oxygenase-1—Nrf2/HO-1) [52] (Figure 7). Additionally, it promotes neuronal survival and recovery by upregulating brain-derived neurotrophic factor (BDNF) and modulating apoptosis and autophagy pathways [53,54]. The studies presented in the following sections collectively illustrate luteolin’s potential as a nutraceutical compound capable of mitigating inflammation-associated neurotoxicity and tissue injury.

#### 3.1.4. Apigenin

Apigenin (5,7-Dihydroxy-2-(4-hydroxyphenyl)-4H-chromen-4-one) is a naturally occurring compound with a flavone core structure, with varied bioactivities, including anti-inflammatory, anti-toxicant, and anti-cancer [55]. It can be found in various fruits and vegetables, such as vegetables from the *Apiaceae* family, citrus fruits, thyme, chamomile, onions, and spices [56]. However, its oral bioavailability is relatively low due to its low solubility in lipids and water [57].

Structurally, apigenin is characterized by OH groups at positions 4′, 5, and 7 (Figure 6). The presence of C5–OH, C7–OH, C2=C3, and C4=O functional groups in apigenin and other flavonoids is associated with enhanced anti-inflammatory effects [58,59]. Oxidative stress, α-glucosidase, and α-amylase inhibitory activities of apigenin have been shown to be attributed to the presence of six double bonds in its two aromatic rings. In particular, double bonds between C-2 and C-3 are possibly crucial factors, and hydroxyls on rings A (C-7 and C-5) and B (C-4′) might serve as augmenters for such activity. Hydroxyl groups of apigenin are also suggested to be crucial moieties to chelate the zinc ions, thus inactivating the ACE activity [59,60]. The hepatoprotective activity of apigenin, which is through regulating the Nrf2/Kelch-like ECH-associated protein 1 (Keap1) antioxidant pathway, NF-κB signaling pathway, the release of pro-inflammatory cytokines, and apoptosis-related factors, has also been shown to be attributed to the presence of a C2=C3 double bond and the hydroxyl group of C4′ [57,59] (Figure 7).

Regarding its mechanisms of action, studies demonstrate that apigenin reduces the production of pro-inflammatory cytokines (e.g., IL-1β, TNF-α, and IL-6) by blocking the transcription factor NF-κB, inhibiting caspase-1, destabilizing NLR family pyrin domain containing 3 (NLRP3) inflammasome assembly, and protecting ERK1/2 [61]. In vitro, apigenin reduces the expression of COX-2, iNOS, and adhesion molecules involved in the leukocyte recruitment from the bloodstream to inflammatory sites [62]. Furthermore, this flavonoid can neutralize ROS and increase the expression and activity of antioxidant enzymes, such as SOD and GPx [63] (Figure 7).

#### 3.1.5. Epicatechin

Epicatechin [2R,3R)-2-(3,4-dihydrophenyl)-3,4-dihydro-1(2H)-benzopyran-3,5,7-triol] is a member of the flavonol class [64], which can be found in fruits such as pears, black grapes, apples, blackberries, raspberries, and cherries, also in broad beans, green tea and chocolate [65].

Structurally, epicatechin has five OH groups in the positions 3, 5, 7, 4′and 5′, which, together with the oxo chemical group, allow for accepting electrons, conferring antioxidant activity (Figure 6) [66]. Indeed, as part of the class of flavonoids called catechins [(+)-epicatechin, (−)-epicatechin (epicatechin), (+)-catechin, and (−)-catechin], epicatechin shows antioxidant and anti-inflammatory activities, which attenuate oxidative stress and improve hepatic enzyme profiles, as well as enable its neuroprotective properties [66,67]. However, when present in free form, gallate or glucuronide, catechins can undergo chemical modifications such as hydroxylation, oxidation, O-methylation, and sulfation [64].

Among catechins, compared with other flavonoids, the stereochemical configuration of epicatechin profoundly influences its absorption, and when present in the form of dimers, trimers, or oligomers, they are more relevant to the human diet, which helps to explain its physiological effects. After absorption in the small intestine, epicatechin is rapidly converted into conjugated metabolites (e.g., sulfates, glucuronides, and methylated compounds), which exert most of the biological effects [64].

Regarding its mechanism of action, epicatechin decreases the production of ROS by inhibiting the nicotinamide adenine dinucleotide phosphate (NADPH) oxidase (NOX) enzymatic complex and the production of superoxide anion (O_2_•^−^), directly contributing to the reduction in oxidative damage. Furthermore, it modulates intracellular signaling pathways such as PI3K/Akt, MAPKs, and NF-κB, and reduces the expression of pro-inflammatory molecules (COX-2 and iNOS) and cytokines (IL-1β, TNF-α, and IL-6), as well as adhesion molecules [e.g., intercellular adhesion molecule 1 (ICAM-1) and vascular cell adhesion molecule 1 (VCAM-1)] (Figure 7). Together, it acts by decreasing microglial activation and the release of neurotoxic mediators [64].

## 4. Reactive Species, Cellular Damage, and Inflammation

This section presents a brief overview of the main factors and pathways involved in oxidative stress modulation and inflammation and provides a necessary conceptual basis for understanding studies involving flavonoids or flavonoid-containing nutraceuticals in the treatment of inflammatory conditions.

Box 1 **Summary of the main reactive species (free radicals and non-free radicals) involved in oxidative stress and inflammation [68].**

Major reactive species in oxidative stress and inflammation.
**REACTIVE OXYGEN SPECIES—ROS**

**Superoxide anion (O_2_^−^):** Primary reactive oxygen species generated during cellular metabolism. It participates in redox signaling and serves as a precursor for the formation of additional ROS.
**Hydrogen Peroxide (H_2_O_2_):** Relatively stable ROS involved in intracellular signaling pathways and oxidative stress responses.
**Hydroxyl radical (OH•):** Highly reactive ROS capable of rapidly damaging lipids, proteins, and nucleic acids.
**Hydroperoxyl radical (HO^−^_2_):** Protonated form of superoxide anion that contributes to lipid peroxidation and oxidative damage to biomolecules.

**REACTIVE NITROGEN SPECIES—RNS**

**Nitric Oxide (NO):** Reactive nitrogen species involved in host defense, inflammatory signaling, vasodilation, and regulation of cellular function.
**Peroxynitrite (ONOO^−^):** Potent oxidizing and nitrating agent formed by the reaction between NO and superoxide anion, capable of inducing oxidative damage to lipids, proteins, and DNA.

ROS are the most common and well-understood reactive species, including highly reactive free radicals due to the presence of unpaired electrons (e.g., O_2_•^−^) and non-radical oxygen intermediates (e.g., hydrogen peroxide—H_2_O_2_) (Box 1) [69]. Endogenous ROS are produced by the mitochondria, which account for approximately 20% of hepatocyte volume and are present in an estimated 1000 to 2000 mitochondria per cell [70]. In addition to mitochondria, other cellular structures such as the plasma membrane, endoplasmic reticulum (ER), and peroxisomes also contribute to ROS production [71]. The use of molecular oxygen (O_2_) as the final electron acceptor in mitochondrial electron transport chains (ETC), as well as the metabolic activity of cytochrome P450, promotes continuous ROS generation [72]. In parallel, exogenous stimuli like ionizing radiation, UV rays, tobacco smoke, pathogen infections, environmental toxins, and exposure to herbicide/insecticides also trigger ROS production in vivo [68].

The production of ROS, mainly O_2_•^−^, is not only a byproduct of cellular aerobic metabolism but also plays a functional role in cell signaling and immune defense. O_2_•^−^, for example, plays an important role in the defense against microorganisms, effectively serving as a broad-spectrum antibiotic [73] and, at physiological concentrations, participates in redox regulation [17]. However, when its generation exceeds the cellular antioxidant capacity, oxidative stress occurs, which is closely related to the development of tissue damage, cell death, and amplification of inflammatory responses [17].

O_2_•^−^ can undergo dismutation into H_2_O_2_ that quickly and readily diffuses through cell membranes, primarily through aquaporins—membrane proteins that transport water across cell membranes in response to osmotic gradients created by active solute transport—making it the main paracrine secondary messenger of ROS involved in redox signaling cascades [74,75,76].

Under physiological conditions, ROS are maintained at low concentrations by means of antioxidant systems, which can be classified into enzymatic and non-enzymatic components [17]. Among the enzymatic components, GPx, SOD, and GSH can be cited as important antioxidant enzymes for maintaining redox homeostasis (Box 2) [17,77]. While SOD promotes the conversion of O_2_•^−^ into H_2_O_2_, GPx catalyzes the reduction of H_2_O_2_ into water and alcohol, thereby abating its toxicity [78,79]. Among the non-enzymatic components, vitamin C (ascorbic acid), vitamin E (α-tocopherol), vitamin A (β-carotene), and flavonoids (e.g., quercetin, hesperidin, and naringenin) are the best characterized [17]. All these components are important for maintaining redox homeostasis by blocking ROS [17].

When this balance is disrupted (establishment of oxidative stress), the excess of ROS promotes oxidative modifications in biomolecules, such as damage to glycolipids, phospholipids, and cholesterol. Lipid peroxidation is mainly mediated by hydroxyl (OH•) and hydroperoxyl (HO^−^_2_) radicals, targeting C-C double bond(s), especially polyunsaturated fatty acids, promoting a hydrogen abstraction from a carbon and an O_2_ insertion, forming lipid peroxyl radicals and hydroperoxides [80]. As secondary products of this process, different aldehydes can be formed, such as propanal, hexanal, 4-hydroxynonenal, and malondialdehyde (MDA), with the latter being widely used as an indirect marker of tissue lipid peroxidation. It is worth noticing that enzymes involved in liver metabolism and inflammation, such as lipoxygenases (LOX), cyclooxygenases (COX), and cytochrome P450, can also promote lipid oxidation under oxidative stress conditions [68].

Box 2 **Summary of the main oxidative stress and antioxidant biomarkers.**

Oxidative stress biomarkers and antioxidant defense.
**ANTIOXIDANT DEFENSE**

**Glutathione peroxidase (GPx):** Antioxidant enzyme that catalyzes the reduction in hydrogen peroxide and lipid hydroperoxides using glutathione as a cofactor [81].
**Reduced glutathione (GSH):** Endogenous non-enzymatic antioxidant. It can directly function as an antioxidant and indirectly as a cofactor for enzymes such as glutathione peroxidases, glutathione S-transferases, and glyoxalases that have antioxidant and detoxification roles [82].
**Superoxide dismutase (SOD):** An enzyme that catalyzes the dismutation of superoxide anion into hydrogen peroxide. Although hydrogen peroxide is still an active ROS, it can be metabolized to molecular oxygen and water; thus, this is an important antioxidant enzyme [83].
**Catalase (CAT):** An enzyme responsible for converting hydrogen peroxide into water and molecular oxygen [84].
**Glutathione S-transferase (GST):** A detoxifying enzyme that catalyzes the conjugation of glutathione to electrophilic and oxidized molecules [85].
**Heme-oxygenase-1 (HO-1):** A cytoprotective enzyme induced during oxidative stress that metabolizes heme into biliverdin, carbon monoxide, and free iron [86].
**Nuclear factor erythroid-related factor 2 (Nrf2):** A redox-sensitive transcription factor that regulates antioxidant, anti-inflammatory, and cytoprotective genes [87].

**ANTIOXIDANT CAPACITY ASSAYS**

**Total sulfhydryl groups (TSH):** Indicative of endogenous thiol-containing antioxidant molecules [88].
**Ferric reducing antioxidant power (FRAP):** Assay that evaluates the antioxidant capacity of biological samples through the reduction of ferric iron (Fe^3+^) to ferrous iron (Fe^2+^) [89].
**ABTS radical scavenging assay:** Measures the ability of a sample to neutralize the ABTS•+ radical cation, representing total endogenous antioxidant capacity [90].
**DPPH assay:** Evaluates free radical scavenging activity based on reduction in the DPPH radical [91].

**OXIDATIVE DAMAGE BIOMARKERS**

**Malondialdehyde (MDA):** The level of this byproduct is an important parameter to quantitate lipid peroxidation (LPO), representing oxidative stress. It reacts with thiobarbituric acid (TBA), forming the colored TBARS complex (thiobarbituric acid reactive substances) [68,92].
**Protein carbonyl (PCO):** Marker of irreversible oxidative protein modification [93].
**Inducible nitric oxide synthase (iNOS):** Enzyme induced during inflammatory responses that promotes high-output nitric oxide production and contributes to oxidative and nitrosative stress [94].

Regarding inflammation, experimental models demonstrate that the excessive release of ROS, such as O_2_•^−^, triggers nociceptive and inflammatory responses mediated by the sensitization of nociceptive neurons, recruitment of leukocytes, and increased expression of pro-inflammatory cytokines (e.g., TNF-α, IL-1β, and IL-6), as well as activation of the transcription factor NF-κB and expression of COX-2 [71]. Furthermore, during inflammation, phagocytes such as neutrophils and macrophages produce O_2_•^−^ by the activity of NADPH oxidase [95,96]. In addition, 5-lipoxigenase and cyclooxygenase-2 also produce O_2_•^−^ as a byproduct [97].

In this context, several studies demonstrate that the use and/or administration of bioactive flavonoids is effective in inhibiting inflammation and oxidative stress. Jeon et al. (2020) observed that the administration of puerarin (one of the main components of the root of *Pueraria lobata*) significantly inhibited inflammation through the downregulation of NF-κB and inflammatory mediators, in addition to exhibiting antioxidant effects through the regulation of Nrf2 and antioxidant enzymes [98]. The administration of cerium-luteolin ion nanocomplexes (CeLutNCs) was also effective in reducing the inflammatory response and oxidative stress in an animal model of acute kidney injury (AKI) and acute lung injury (ALI) [99]. Similarly, other flavonoids such as quercetin [100,101], naringenin [102,103], diosmin [104,105,106], hesperidin methyl chalcone [107,108,109], and *trans*-chalcone [110,111,112,113] exhibit antioxidant and anti-inflammatory effects in different disease models.

The following sections detail the protective and anti-inflammatory effects of quercetin, kaempferol, luteolin, apigenin, and epicatechin in different systems, such as the hepatic, nervous, and pulmonary systems.

## 5. Protective and Anti-Inflammatory Effects of Flavonoids in Different Systems

As mentioned in the aims and methodology, the three most investigated areas on flavonoid bioactivity were liver—12%, nervous system—11%, and lungs—10%. This section addresses the activity and mechanisms of quercetin, kaempferol, luteolin, apigenin, and epicatechin in those areas since these were the top five flavonoids most studied molecules of this class within 2020–2025.

### 5.1. Flavonoid Effects Against Liver Damage and Inflammation

The liver is an essential organ; among its main functions are (a) blood filtration to remove toxins; (b) bile production for fat digestion; and (c) storage and regulation of nutrients and hormones [114,115,116]. Its crucial role in drug metabolite removal from the bloodstream, particularly lipophilic agents, makes the liver the most affected organ when drug toxicity occurs [117]. Several factors can result in liver toxicity, such as viral infection, alcohol, drug abuse, metabolic and autoimmune disorders, toxins, and other pathogenic agents [118]. These factors can compromise liver function, progressing from an acute injury consisting of inflammation and tissue alterations to chronic damage such as metabolic disorders, steatosis, and end-stage conditions such as cirrhosis and hepatocellular carcinoma. Even though they might share common features, pathophysiologic, clinical, and therapeutic profiles can be distinct [119]. This review mainly focuses on the effects of flavonoids in experimental models for acute liver damage.

Drugs can either be passively internalized by hepatocytes or they can be transported through proteins located in the basolateral plasma membrane, such as the organic anion transporting polypeptides, organic anion transporters, and organic cation transporters [120]. Liver detoxification usually occurs via phase II conjugation through glucuronidation, acetylation, sulphation, glutathione conjugation, among other transformations, allowing their excretion by multi-drug resistance-associated protein transporters at phase III. Nonetheless, when phase I transformation is required, oxidation and reduction by cytochrome P450 result in the production of reactive metabolites. Drug-induced liver injury is often primed by reactive metabolites and their covalent binding to cellular proteins [121]. Therefore, the immune response plays an important role in drug-induced hepatic damage: the stress from dynamic drug metabolism can sensitize hepatocytes and induce liver cell death, a process that can be further aggravated by the immune cells [121]. Although this process may present itself in a gradual evolution manner, some toxicants are known to promote an acute liver toxicity, such as thioacetamide, carbon tetrachloride, and lipopolysaccharides (LPS) [118].

Tissue damage, including hepatic architecture disarrangement and dilation of sinusoidal space, leukocyte infiltration in the portal tract, granular vacuolization, and hydropic changes, are common findings in acute liver damage, as well as the elevation of alanine transaminase (ALT) and aspartate transaminase (AST), alkaline phosphatase (ALP), γ-glutamyl transferase (γGT), and lactate dehydrogenase (LDH) [122].

Increased hepatic ROS, MDA, NO, and myeloperoxidase (MPO) are also often described in liver damage, while antioxidant enzymes such as GSH, CAT, SOD, and GPx are usually downregulated, although some experimental models, such as smoke-induced liver damage, may promote increased levels of antioxidant enzymes depending on the time point as a feedback protective mechanism [123,124,125].

**Figure 8:**
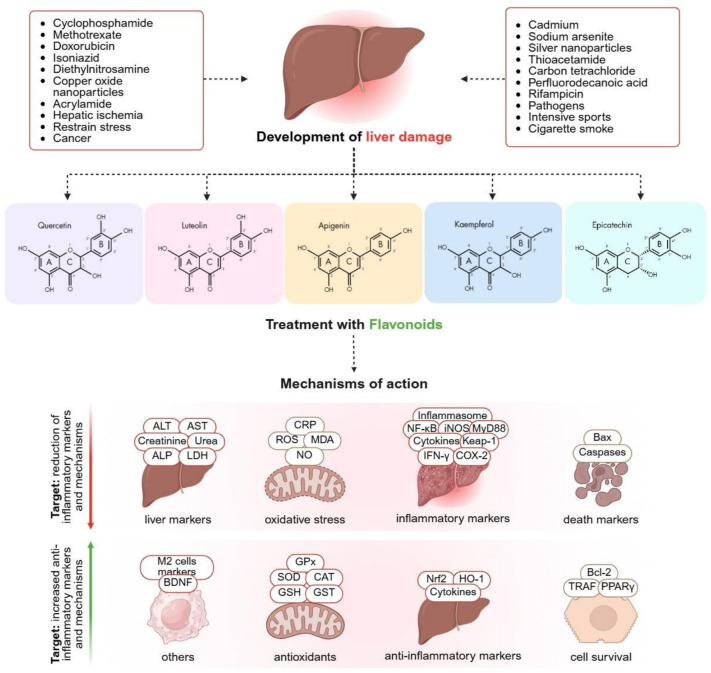
Shared mechanisms of action among flavonoids for the reduction in liver damage. Treatment with flavonoids, such as quercetin, luteolin, apigenin, kaempferol, and epicatechin, acts on the modulation of multiple cellular and molecular mechanisms. These compounds promote the reduction in markers of liver injury (ALT, AST, ALP, LDH, creatinine, and urea), oxidative stress (ROS, MDA, NO, and CRP), inflammation (NF-κB, iNOS, cytokines, inflammasome, MyD88, Keap1, IFN-γ, and COX-2), and cell death (Bax and caspases). Simultaneously, they stimulate endogenous antioxidant systems (SOD, CAT, GPx, GSH, and GST), anti-inflammatory pathways (Nrf2, HO-1, and cytokines), M2 cell markers, and cell survival mechanisms (Bcl-2, TRAF, and PPARγ). The acronyms mentioned are described in the “Abbreviations” section at the end of the article. Created in BioRender. Verri Jr, W. A. (2026) https://BioRender.com/krbxie3 (accessed on 5 June 2026).

Moreover, increased inflammatory enzymes such as COX, pro-inflammatory cytokines, the activation of the transcription factor NF-κB pathway, and pro-apoptotic markers are also commonly found in hepatic damage [126]. Considering the important role of ROS in liver toxicity and disease progression, the notorious antioxidant activities of flavonoids support a rational basis for envisaging their potential role in hepatic protection. Figure 8 summarizes the agents that cause liver damage and the mechanisms of action shared among the flavonoids mentioned in this article.

#### 5.1.1. Cyclophosphamide-Induced Hepatotoxicity

Among the drugs known to induce liver toxicity, cyclophosphamide-induced damage has been one of the most studied experimental models to analyze the effects of the selected flavonoids over the last six years. Cyclophosphamide is a potent immunosuppressive and antineoplastic agent that can cause multi-organ toxicity at high doses (over 120 mg/kg) [127]. Structurally, it is a nitrogen mustard that is effective against neoplasia through alkylation. Its hepatic bioactivation occurs mainly via the cytochrome P450 2B6 enzyme [128], generating the metabolites phosphoramide mustard and acrolein. Its therapeutic use is limited due to the severe side effects, especially hepatotoxicity attributed to oxidative stress, that occur not only as a result of metabolic functions but also due to the biotransformation of xenobiotics, as the enzyme cytochrome P450 can leak electrons to O_2_ during its catalytic cycle and generate superoxide radicals, resulting in redox imbalance and promoting an inflammatory response [124,129,130]. Therefore, cyclophosphamide-induced hepatic damage involves structural disruption, lipid peroxidation, and antioxidant depletion [126,131].

Table 1 summarizes the studies published between 2020 and 2025 that investigated selected flavonoids for protection against cyclophosphamide-induced liver damage. The use of quercetin to improve cyclophosphamide-induced hepatotoxicity can reduce MDA, protein carbonyl (PCO), ALT, AST, ALP, total bilirubin, albumin, urea, and creatinine levels. The flavonoid reduces liver function disruption and morphological degeneration, reverses body weight loss, food intake reduction, and total antioxidant capacity [124,132,133]. Improvement in hepatic congestion and dilatation of the central vein, perivascular fibrosis and hepatocellular vacuolation and necrosis induced by cyclophosphamide were also observed after treatment with a nanoformulation of quercetin and ameliorated total antioxidant capacity [134].

Additionally, quercetin inhibited oxidative-inflammatory stress along with the hepatic activities of indoleamine-2,3-dioxygenase (IDO) and tryptophan 2,3-dioxygenase (TDO) enzymes, which are part of the kynurenine pathway as tryptophan catabolic enzymes and can be elevated in inflammatory conditions characterized by elevated ROS and in liver destruction [126,135]. Anti-inflammatory effects of quercetin include cytokine production inhibition and the negative modulation of the transcription factor NF-κB pathway. Quercetin was able to inhibit the expression of tissue pro-apoptotic markers, therefore indicating reduced hepatic cell death [132,136]. Moreover, it promoted a switch in macrophage profile, reducing the M1 marker (inflammatory macrophage phenotype) cluster of differentiation (CD) 86 and upregulating CD163 expression, an M2 macrophage (repair macrophage phenotype) polarization marker [132].

The effects of quercetin on brain function after cyclophosphamide-induced hepatotoxicity were also investigated, showing improvements in working-memory and anxiety-related behaviors. Quercetin was able to modulate cerebral cortex neurotransmitters, reversing alterations in the levels of acetylcholine, dopamine, and BDNF, while reducing serotonin levels and astrocyte immunoreactivity [133].

The flavonoid apigenin has also shown encouraging effects against cyclophosphamide-induced hepatotoxicity, reducing liver function markers serum levels, promoting an anti-inflammatory response by reduced oxidative stress, pro-inflammatory cytokines production, and NF-kB activation. Apigenin promoted the upregulation of antioxidant pathways and reduced hepatocyte oxidative deoxyribonucleic acid (DNA) damage and apoptosis [123].

In summary, these findings reinforce the potential of flavonoids as complementary strategies to mitigate chemotherapy-associated toxicity. Flavonoids lead to changes in the phenotype of immune cells, reducing local tissue damage and also benefiting systemic outcomes in terms of improving memory and anxiety behaviors. Thus, supporting the relevance of antioxidant and anti-inflammatory mechanisms of flavonoids.

**Table 1:** Summary of the literature on selected flavonoids against cyclophosphamide-induced liver damage from 2020 to 2025.

Cyclophosphamide-induced liver toxicity				
AUTHORS	TREATMENT	SUBJECTS	MODEL INDUCTION	MAIN FINDINGS
Doustimotlagh et al. (2020) [124]	• Quercetin [75 mg/kg] • Oral administration	• Wistar rats [200–250 g]	• Cyclophosphamide [200 mg/kg] • Intraperitoneal administration • Day 10	Quercetin effects compared to cyclophosphamide group: • ↓ MDA • ↓ PCO • ↓ ALT
Al-Amarat et al. (2022) [123]	• Apigenin [20 and 40 mg/kg] • Oral administration • For 15 days	• Male Wistar rats [200–220 g]	• Cyclophosphamide [150 mg/kg] • Intraperitoneal administration • Day 16	Apigenin effects compared to cyclophosphamide group: • ↓ Serum ALT, AST, ALP, and LDH • ↓ ROS (H_2_DCF-DA), MDA, NO, NF-κB p65, iNOS, TNF-α, IL-6, oxidative DNA damage (8-oxo-dG), Bax, and caspase-3 • ↑ GSH, SOD, CAT, NQO-1, and Bcl-2 • ↑ Nrf2 and HO-1 mRNA and protein levels
Ebokaiwe et al. (2021) [126]	• Quercetin [50 mg/kg] • Oral administration • Every other day for 7 days	• Albino male Wistar rats [180 ± 2 g]	• Cyclophosphamide [100 mg/kg] • Oral administration • Every other day for 7 days	Quercetin effects compared to cyclophosphamide group: • ↑ SOD, CAT, GPx, GSH concentration, and GST activity • ↑ Thyroid hormones (T3 and T4) • ↓ AST, ALT, ALP, urea and creatinine • ↓ MPO, NO, and IL-6 • ↓ IDO and TDO
Onaolapo et al. (2023) [133]	• Quercetin supplemented diet [100 and 200 mg/kg of feed] • For three weeks	• Male Wistar rats [120–150 g]	• Cyclophosphamide [150 mg/kg/day] • Intraperitoneal administration • Days 1 and 2	Quercetin effects compared to cyclophosphamide group: • ↓ AST, ALT, and MDA • ↓ Creatinine, IL-1β, and TNF-α • ↑ Total antioxidant capacity and IL-10 • ↑ Acetylcholine, dopamine, and BDNF • ↓ Anxiety-related behaviors • ↓ Serotonin and astrocyte immunoreactivity
Ghaly et al. (2023) [134]	• Nano quercetin [50 mg/kg] • Oral administration • For 10 days	• Albino male adult rats [180 ± 20 g] • 12–14 weeks old	• Cyclophosphamide [200 mg/kg] • Intraperitoneal administration • Day 10	Nano quercetin effects compared to cyclophosphamide group: • ↓ AST and MDA levels • ↑ Total antioxidant capacity • Improved hepatic congestion and dilatation of the central vein, perivascular fibrosis and hepatocellular vacuolation, and necrosis
Turedi (2023) [136]	• Quercetin [100 mg/kg] • Oral administration • For 5 days before or after cyclophosphamide inductions	• Female Wistar Albino rats [300–400 g] • 12–16 weeks old	• Cyclophosphamide [200 mg/kg] • Intraperitoneal administration • Day 1 followed by [8 mg/kg per day] • Total of 14 doses	Quercetin liver effects compared to cyclophosphamide group: • ↓ Histopathological structural damage • ↓ Caspase-3 and Bax • ↓ TNF-α and IL-1β
Seker et al. (2025) [132]	• Quercetin [50 mg/kg] • For 14 days	• Mice	• Cyclophosphamide [200 mg/kg] • Days 1 and 7	Quercetin effects compared to cyclophosphamide group: • ↓ ALT, AST, ALP, total bilirubin, and albumin levels • ↓ Bax, caspase-3, TNF-α, IL-1β, NF-κB, and CD86 • ↑ CD163

Abbreviations: 8-oxo-dG (8-Oxo-2′-deoxyguanosine, a main product of DNA oxidation), ALP (alkaline phosphatase), ALT (alanine aminotransferase), AST (aspartate aminotransferase), Bax (Bcl-2 Associated X protein), Bcl-2 (B-cell lymphoma 2), BDNF (brain-derived neurotrophic factor), CAT (catalase), CD86 (cluster of differentiation 86), CD163 (cluster of differentiation 63), GPx (glutathione peroxidase), GSH (reduced glutathione), GST (glutathione S-transferase), H_2_DCF-DA (2′,7′-Dichlorofluorescin Diacetate—fluorescent probe used to determine total cellular ROS levels), HO-1 (heme oxygenase-1), IDO (indoleamine 2,3 dioxygenase), IL-1β (interleukin-1 beta), IL-10 (interleukin-10), IL-6 (interleukin-6), iNOS (inducible nitric oxide synthase), LDH (lactate dehydrogenase), MDA (malondialdehyde), MPO (myeloperoxidase), NF-κB (nuclear factor kappa-light-chain-enhancer of activated B cells), NO (nitric oxide), Nrf2 (Nuclear factor erythroid 2-related factor 2), PCO (protein carbonyl), ROS (reactive oxygen species), SOD (superoxide dismutase), TDO (tryptophan 2,3 dioxygenase), TNF-α (tumor necrosis factor-alpha), ↑ (increase), ↓ (decrease).

#### 5.1.2. Methotrexate-Induced Hepatotoxicity

Methotrexate is a folate reductase inhibitor with anti-inflammatory, antiproliferative, and immunosuppressive effects, commonly used against neoplasia and chronic inflammatory diseases such as rheumatoid arthritis and psoriasis because it inhibits the activation of NF-κB [137]. In contrast, its clinical use is limited by recognized adverse effects, especially the hepatotoxicity that involves stellate cell hypertrophy, steatosis, hepatic fibrosis, and anisonucleosis [138,139]. Table 2 summarizes the studies published between 2020 and 2025 that investigated selected flavonoids for protection against methotrexate-induced liver damage.

The flavonoid epicatechin has shown protective effects against methotrexate-induced liver damage, reducing inflammatory cell infiltration, vascular congestion, and fat deposits. It inhibits liver enzyme elevation, reduces oxidative damage, and inhibits pro-inflammatory cytokines production. Additionally, epicatechin induced antioxidant enzyme activity [140].

Apigenin is also effective in alleviating methotrexate-induced damage, reducing hepatic function markers, inducing antioxidant enzyme activity, improving liver morphology, and downregulating pro-inflammatory mediators [141,142]. It also attenuated oxidative stress and tissue injury markers, histopathological alterations, and apoptosis [142].

The flavonoid luteolin also showed protective effects against methotrexate-induced toxicity, reducing hepatic enzyme levels, lipidic peroxidation, hepatocyte cell death, and inflammatory markers such as cytokines and the activation of the transcription factor NF-κB. Furthermore, luteolin induced antioxidative and anti-apoptotic activities [143]. Thus, oxidative stress and NF-κB were the main targets of flavonoids in methotrexate liver damage with positive outcomes in hepatocyte survival and function, as well as tissue preservation. These findings suggest that flavonoids may act on central mechanisms involved in methotrexate hepatotoxicity and may represent promising adjuvant strategies to reduce treatment-related liver damage.

**Table 2:** Summary of the literature on selected flavonoids against methotrexate-induced liver damage from 2020 to 2025.

Methotrexate-induced liver toxicity				
AUTHORS	TREATMENT	SUBJECTS	MODEL INDUCTION	MAIN FINDINGS
Azadnasab et al. (2021) [140]	• Epicatechin [100 mg/kg/day] • Oral administration • For 9 days	• Male mice [25–30 g] • 7 weeks old	• Methotrexate [20 mg/kg] • Intraperitoneal administration • On day 5, 2 h before epicatechin treatment	Epicatechin effects compared to methotrexate group: • ↓ Serum ALT and AST • ↓ Histological damage composed of inflammatory cell infiltration, vascular congestion, and fat deposit • ↓ MDA, NO, IL-1β, and TNF-α • ↑ GPx, SOD, CAT, and GSH
Sahindokuyucu-Kocasari et al. (2021) [142]	• Apigenin [3 mg/kg/day] • Intraperitoneal administration • For 7 days	• Male CD-1 mice [20–30 g] • 10–12 weeks old	• Methotrexate [20 mg/kg] • Intraperitoneal administration • Day 4	Apigenin effects compared to methotrexate group: • ↓ Serum AST, ALT, and creatinine • ↓ Histological damage • ↓ MDA, CRP, iNOS, caspase-3, G-CSF, and iNOS expressions • ↑ SOD1, GPx, CAT, and GSH activities
Goudarzi et al. (2021) [141]	• Apigenin [20 mg/kg] • Oral administration • For 9 days	• Male Wistar rats [200–220 g]	• Methotrexate [20 mg/kg] • Intraperitoneal administration • Day 7	Apigenin effects compared to methotrexate group: • ↓ ALP, AST, and ALT • ↓ MDA, TNF-α, NO, and IL-1β • ↑ GSH, CAT, GPx, and SOD
Dar et al. (2021) [143]	• Luteolin [50 mg/kg] • Oral administration • For 14 days	• Male Wistar Albino rats • 8 weeks old	• Methotrexate [20 mg/kg] • Intraperitoneal administration • Day 9	Luteolin effects compared to methotrexate group: • ↓ AST, ALT, creatinine, urea, uric acid, and MDA • ↓ NF-κB, TNF-α, and IL-1β • ↓ Bax • ↑ GSH, Nrf2, and Bcl-2

Abbreviations: ALP (alkaline phosphatase), ALT (alanine aminotransferase), AST (aspartate aminotransferase), Bax (Bcl-2 Associated X protein), Bcl-2 (B-cell lymphoma 2), CAT (catalase), CRP (C-reactive protein), G-CSF (Granulocyte-Colony Stimulating Factor), GPx (glutathione peroxidase), GSH (reduced glutathione), IL-1β (interleukin-1 beta), iNOS (inducible nitric oxide synthase), MDA (malondialdehyde), NF-κB (nuclear factor kappa B), NO (nitric oxide), Nrf2 (Nuclear factor erythroid 2-related factor 2), SOD (superoxide dismutase), TNF-α (tumor necrosis factor-alpha), ↑ (increase), ↓ (decrease).

#### 5.1.3. Doxorubicin-Induced Liver Toxicity

Doxorubicin is an antineoplastic agent widely used in chemotherapy for various cancers due to its potency and broad spectrum of action [144]. This is an effective drug that acts by intercalating with the DNA double helix, inhibiting nucleic acid and protein synthesis [145]. Nonetheless, its metabolism by cytochrome P450 reductase into a semiquinone radical generates ROS, promoting oxidative damage and inducing calcium overload and mitochondrial dysfunction, resulting in toxicity and limiting its applicability [146]. One of the strategies developed to minimize the toxicity caused by doxorubicin is combined administration with flavonoids [144]. Table 3 summarizes the studies published between 2020 and 2025 that investigated selected flavonoids in the protection against doxorubicin-induced liver damage.

Quercetin was effective in reducing histological liver changes induced by doxorubicin, as well as hepatic enzyme levels. Moreover, it prevented the elevation in liver lipid peroxidation and the reduction in antioxidant response. The flavonoid was also able to reduce the tumor markers alpha-fetoprotein (AFP) and carbohydrate antigen 19.9 (CA19.9) in serum and inhibit the expression of pro-apoptotic and pro-inflammatory markers, while inducing an antioxidant response [147]. Luteolin was another flavonoid with protective effects on liver toxicity caused by doxorubicin in rats. It reduced hepatic damage markers, oxidative stress, inflammation, hepatocyte cell death, and promoted an antioxidant response [146]. Thus, the antioxidant mechanisms of flavonoids explain their protective effects in preserving the liver.

In general, the available evidence suggests that quercetin and luteolin exert hepatoprotective effects against doxorubicin-induced liver injury, mainly by reducing oxidative stress, inflammation, apoptosis, and histopathological damage, while restoring antioxidant defenses. These effects are particularly relevant because oxidative imbalance and mitochondrial dysfunction are central mechanisms of doxorubicin toxicity.

#### 5.1.4. Flavonoid Effects Against Liver Damage and Inflammation Caused by Chemical Compounds

Table 4 summarizes the studies published between 2020 and 2025 that investigated the selected top 5 flavonoids in protecting against liver damage induced by chemical or physical agents.

The hepatoprotective effects of the flavonoid quercetin were studied by Sanjay et al. (2021) against toxicity caused by the tuberculosis treatment protocol based on isoniazid and rifampicin (first-line antibiotics used in combination to treat active tuberculosis). This standard protocol treatment induces severe hepatotoxicity and leads to liver failure. Quercetin prevented hepatic enzyme elevation and reduced the severity of hepatic necrosis and inflammation in the histological analysis. Furthermore, quercetin treatment induced the activation and expression of the antioxidant transcription factor Nrf2, while inhibiting the production of the inflammatory mediators such as high mobility group box-1 (HMGB-1) and interferon γ (IFN-γ) [148]. The effects of quercetin were also investigated by Mukherjee et al. (2022) in an experimental model of liver injury induced by diethylnitrosamine (a potent hepatocarcinogenic and toxic compound). The group observed that quercetin improved hepatic function markers, promoted the activity of antioxidant enzymes, reduced oxidative stress, and inflammation [149].

Another experimental model that demonstrated the hepatoprotective effects of quercetin was based on the induction of sub-chronic liver toxicity by copper oxide nanoparticles. Haroun et al. (2024) observed that hepatic copper bioaccumulation induced an elevation of serum ALT and reduced arginase activity, which is indicative of the synthetic ability of the liver. The experimental model also increased oxidative stress, lipid peroxidation, inflammation, apoptosis, promoted pathological changes in the liver architecture and severe DNA fragmentation. On the other hand, quercetin was effective in reducing the sub-chronic liver toxicity due to its antioxidant, anti-inflammatory, and anti-apoptotic properties, as well as inhibiting DNA damage and showing its potential as a good metal chelator [150].

In another experimental model of hepatotoxicity induced by nanoparticles, Sousa et al. (2025) investigated quercetin’s potential against the pro-inflammatory effects of silver nanoparticles. The group confirmed the hepatoprotective effects of quercetin that reversed liver damage, inhibiting inflammatory markers and promoting an antioxidant response [151].

The use of nano-formulations was also investigated as an alternative to improve drug delivery. The comparison between the effectiveness of quercetin in its free form and via nanoparticles was investigated by Atta et al. (2025) in a model of hepatotoxicity induced by acrylamide (a toxic chemical compound and probable carcinogen). The authors attributed the modulation of MAPK/NF-κB/NLRP3 signaling pathways as the mechanisms of action involved in the improvement in liver toxicity. Moreover, the nanoparticles showed enhanced bioavailability and targeted delivery, indicating that the nano-formulation outperforms free quercetin in the reduction of oxidative stress, inflammation, and apoptosis [152].

In an experimental model of hepatotoxicity induced by cadmium (a toxic metal), Liu et al. (2024) observed that quercetin reduced liver function and oxidative stress markers and inhibited the production of inflammatory cytokines and the activation of the NF-kB pathway. Moreover, the flavonoid inhibited liver cell death by apoptosis and promoted the activity of antioxidant enzymes [153].

Hussein et al. (2021) studied the effects of quercetin against thioacetamide-induced acute liver toxicity (a potent hepatotoxin used to induce liver fibrosis, cirrhosis, and cancer), finding improved liver function and tissue integrity, lower reactive species and lipid peroxidation levels, along with increased antioxidant activity observed by the downregulation of Keap1, while Nrf2 and HO-1 were increased. Hepatic TNF-α expression and apoptotic cells were also inhibited by quercetin [118].

Hejazi et al. (2024) investigated the protective effects of Epicatechin against liver toxicity caused by sodium arsenite (a compound widely known as a potent cellular toxin, carcinogen, and teratogen). The flavonoid improved the levels of hepatic function markers and promoted an antioxidant response, while inhibiting liver apoptosis and inflammation [154].

Lin et al. (2020) investigated the hepatoprotective effects of quercetin against carbon tetrachloride-induced acute liver injury (a compound highly toxic). Liver function markers and NF-κB signaling pathway activation were inhibited in quercetin-treated animals, indicating its effect in liver protection by reduced inflammation [155]. The flavonoid apigenin also showed liver protection against carbon tetrachloride-induced acute liver injury. Yue et al. (2020) demonstrated anti-inflammatory and antioxidative effects of apigenin by reducing the levels of hepatic enzymes, promoting an antioxidant response, and inhibiting pro-inflammatory cytokine production and lipid peroxidation. Interestingly, apigenin’s protective effects were associated with the inhibition of the non-canonical NF-κB pathway [156].

These data lead to the conclusion that flavonoids consistently inhibit liver damage caused by varied chemical agents that are applied to study liver damage, such as carbon tetrachloride, but also toxic agents such as sodium arsenite or medication (isoniazid and rifampicin). Thus, the nutraceutical potential of flavonoids is supported by not only targeting inflammatory signaling (e.g., MAPK, NFκB and NLRP3), but also by stimulating antioxidant programs regulated by Nrf2, and their intrinsic structural antioxidant activities.

**Table 4:** Summary of the literature on selected flavonoids against liver damage and inflammation induced by chemical compounds, from 2020 to 2025.

Liver damage and inflammation induced by chemical compounds				
AUTHORS	TREATMENT	SUBJECTS	MODEL INDUCTION	MAIN FINDINGS
Isoniazid- and rifampicin-induced liver toxicity				
Sanjay et al. (2021) [148]	• Quercetin [25; 50 or 100 mg/kg] • For 28 days	• Male Wistar rats [200–250 g]	• Isoniazid [150 mg/kg] and rifampicin [150 mg/kg] • Oral administration	Quercetin effects compared to stimulus group: • ↓ ALT, AST, ALP, and bilirubin • ↓ Hepatic necrosis and inflammation • ↑ Nrf2 activation and expression • ↓ HMGB-1, IFN-γ, and NF-κB
Diethylnitrosamine-induced liver injury				
Mukherjee et al. (2022) [149]	• Quercetin [60 mg/kg] daily • Intraperitoneal administration • For 2 weeks	• Wistar rats [100–120 g]	• Diethylnitrosamine [10 mL/kg] • Intraperitoneal administration • After 2 weeks of treatment	Quercetin effects compared to stimulus group: • ↓ AST, ALT, ALP, γGT, and bilirubin • ↓ MDA, protein oxidation and nitrites, and collagen levels • ↑ GST, glycogen, and CAT • ↓ COX-2 and iNOS
Copper oxide nanoparticles-induced hepatic damage				
Haroun et al. (2024) [150]	• Quercetin [50 mg/kg] • Oral administration • For 8 weeks	• Adult male mice [20–30 g]	• Copper oxide nanoparticles [100 mg/kg] • Oral administration • For 8 weeks	Quercetin effects compared to stimulus group: • ↓ Caspase-3, Bax, TNF-α, MDA, and NO • ↑ Arginase and SOD
Acrylamide-induced liver toxicity				
Atta et al. (2025) [152]	• Quercetin nanoparticles [25 mg/kg/day] • Intraperitoneal administration • For 5 days	• Male albino rats [200–250 g] • 8–9 weeks old	• Acrylamide [50 mg/kg] • Oral administration • For 15 days	Quercetin nanoparticles effects compared to stimulus group: • ↓ ALT, AST, and MDA • ↓ MAPK, NF-κB, NLRP3, IL-1β, and IL-6 • ↓ Caspase-3, Bax, and p53 • ↑ GSH and GPx
Cadmium-induced hepatotoxicity				
Liu et al. (2024) [153]	• Quercetin [100 mg/kg/day] • Oral administration • Five times per week	• Male C57BL/6J mice [20 g] • 6–8 weeks old	• Cadmium [2.5 mg/kg/day] • Oral administration • Five times per week	Quercetin effects compared to stimulus group: • ↓ AST, ALT, ALP, MDA, and LDH • ↓ IL-1β, TNF-α, and IL-6 • ↑ Bcl-2, SOD, CAT, GPx, and GSH • ↓ p-Akt, p-PI3K, Bax, caspase-9, caspase-3, and NF-κB
Sodium arsenite-induced hepatotoxicity				
Hejazi et al. (2024) [154]	• Epicatechin [100 mg/kg] • Oral administration • For 2 weeks	• Male mice [20–25 g]	• Sodium arsenite [10 mg/kg] • Oral administration • For 5 weeks	Epicatechin effects compared to stimulus group: • ↑ SOD, CAT, GPx, and GSH • ↓ AST, ALT, ALP, NO, and TNF-α • ↓ TLR4, NF-κB, Bcl-2, and caspase-3
Silver nanoparticles-induced inflammation				
Sousa et al. (2025) [151]	• Quercetin [1 mg/kg] • Intraperitoneal administration • For 14 days	• C57BL/6J mice [21–27 g] • 12 weeks old	• Silver nanoparticles [10 mg/kg] • Oral administration • For 14 days	Quercetin effects compared to stimulus group: • ↑ SOD1, CAT, and CD4 T cells
Thioacetamide-induced acute liver toxicity				
Hussein et al. (2021) [118]	• Quercetin [300 mg/kg] • Oral administration • For 8 days	• Wistar albino rats [180–230 g]	• Thioacetamide [50 mg/kg] • Intraperitoneal administration • Day 8	Quercetin effects compared to stimulus group: • ↓ ALT, AST, ALP, bilirubin, ROS, MDA, and NO • ↑ SOD and GSH • ↓ TNF-α and Keap1 • ↑ Nrf2 ans HO-1
Carbon tetrachloride-induced acute liver injury				
Lin et al. (2020) [155]	• Quercetin [120 mg/kg] • Oral administration • For 10 days	• Male BALB/c mice [20 ± 2 g]	• Carbon tetrachloride [10 mL/kg, 0.2% in soybean oil] • Intraperitoneal administration • Day 10	Quercetin effects compared to stimulus group: • ↓ AST and ALT • ↓ NF-κB and p-IκB-α
Yue et al. (2020) [156]	• Apigenin [50, 100, and 200 mg/kg] • Oral administration • For 7 days	• Male ICR mice [18–22 g]	• Carbon tetrachloride [2.5 mL/kg, 10% in olive oil] • Intraperitoneal administration • Day 7	Apigenin effects compared to stimulus group: • ↓ AST, ALT, and MDA • ↑ SOD, GSH, GPx, CAT, and IL-10 • ↓ TNF-α and IL-6 • ↑ TRAF2/3 and c-IAP, • ↓ NIK, RelB, p100, and p52

Abbreviations: p-Akt (phosphorylated protein kinase B), AST (aspartate aminotransferase), ALP (alkaline phosphatase), ALT (alanine aminotransferase), Bax (Bcl-2 Associated X protein), Bcl-2 (B-cell lymphoma 2), CAT (catalase), CD4 (cluster of differentiation 4), c-IAP (Cellular Inhibitor of Apoptosis Proteins), COX-2 (cyclooxygenase-2), GPx (glutathione peroxidase), γGT (gamma-glutamyl transferase), GSH (reduces glutathione), GST (glutathione S-transferase), HMGB-1 (High Mobility Group Box 1), HO-1 (heme-oxygenase-1), IFN-γ (interferon gamma), p-IκB-α (phosphorylated inhibitor of nuclear factor kappa B alpha), IL-1β (interleukin-1 beta), IL-6 (interleukin-6), IL-10 (interleukin-10), iNOS (inducible nitric oxide synthase), Keap1 (Kelch-like ECH-associated protein 1), LDH (lactate dehydrogenase), MAPK (mitogen-activated protein kinase), MDA (malondialdehyde), NF-κB (nuclear factor kappa-light-chain-enhancer of activated B cells), NIK (NF-κB-inducing kinase), NLRP3 (NOD-, LRR- and pyrin domain-containing protein 3), NO (nitric oxide), Nrf2 (Nuclear factor erythroid 2-related factor 2), p-PI3K (phosphorylated phosphoinositide 3-kinase), RelB (a subunit of NF-κB called RELB proto-oncogene), ROS (reactive oxygen species), SOD (superoxide dismutase), TLR4 (toll-like receptor 4), TNF-α (tumor necrosis factor-alpha), TRAF2/3 (Tumor necrosis factor receptor-associated factors 2 and 3), ↑ (increase), ↓ (decrease).

#### 5.1.5. Hepatic Ischemia-Reperfusion Injury

Liver surgery is a condition accompanied by ischemia-reperfusion injury with deleterious outcomes such as hepatic damage and potential organ failure [157]. This life-threatening condition can be mimicked by a blockade of the hepatic portal vein blood supply within a timeframe sufficient to promote ischemia and then followed by reperfusion [158]. Table 5 summarizes the studies published between 2020 and 2025 that investigated selected flavonoids in the protection against hepatic ischemia-reperfusion injury.

Investigating the activity of quercetin, Lin et al. (2025) observed a reduction in liver tissue necrosis and inflammation. These effects appear to be related to an inhibition of macrophage pyroptosis by blocking caspase-8/apoptosis-associated speck-like protein containing a CARD (ASC) interaction. Therefore, the flavonoid could be an effective alternative for the prevention of hepatic ischemia-reperfusion injury in perioperative patients [159]. In agreement, Ferreira-Silva et al. (2022) developed and characterized a stable quercetin liposomal formulation intended to treat ischemia-reperfusion injury. The group observed that the quercetin liposomes reduced inflammatory markers and improved tissue recovery, indicating that quercetin liposomes could also be a promising alternative in hepatic ischemia-reperfusion injury treatment [160]. Chen et al. (2022) investigated the protective effect of the flavonoid kaempferol on the same experimental model. Pretreatment with kaempferol attenuated oxidative stress, inhibited the activation of NF-κB/p65, reduced the release of pro-inflammatory factors, and promoted antioxidant response by activating the Nrf2/HO-1 signaling pathways [161].

Thus, the inhibition of cellular death by pyroptosis seemed to be a relevant mechanism, as well as the targeting of transcription factors balancing inflammation and oxidative stress by inhibition of NF-κB and activation of Nrf2, to explain the application of flavonoids in hepatic ischemia-reperfusion injury.

#### 5.1.6. Pathogen-Related or Pathogen-Induced Liver Damage

Table 6 summarizes the studies published between 2020 and 2025 that investigated selected flavonoids in protecting against pathogen-related or pathogen-induced liver damage.

The mycotoxin zearalenone is produced by fungi of the genus *Fusarium* and can promote immunotoxic, hepatotoxic, and xenogenic effects [162]. Rajendran et al. (2020) observed kaempferol hepatoprotective activity against zearalenone-induced toxicity. This flavonoid decreased inflammation and lipid peroxidation, induced PI3K and Akt phosphorylation, improved antioxidative response, suggesting a possible role of the PI3K/Akt activation on Nrf2 phosphorylation and HO-1 and NAD(P)H-quinone oxidoreductase 1 (NQO-1) expression [163].

To investigate the effectiveness of flavonoids against liver damage in sepsis (extreme and dysregulated exposure of the immune system to an infection, which damages the body’s own tissues and organs), Deng et al. (2025) observed that luteolin was able to modulate liver macrophage polarization, promoting hepatoprotective effects. This flavonoid reduced liver injury and inflammatory response, promoted the change in macrophages from a proinflammatory M1 phenotype to an anti-inflammatory M2 phenotype, and inhibited the Toll-like receptor 4 (TLR4)/myeloid differentiation primary response 88 (MyD88)/NF-κB pathway [164].

Bacterial LPS are amphiphilic macromolecules and the main virulence factor of Gram-negative bacteria. It is formed by three structural parts: (a) an O-specific polysaccharide (OPS), which is covalently linked to (b) an oligosaccharide (core), which is in turn linked to (c) a glycolipid moiety (Lipid A) [165]. Its negative charge is exposed to the external environment and forms a physical barrier to protect the bacteria from potentially harmful agents. This endotoxin is a potent trigger of the inflammatory process, promoting the release of cytokines, chemokines, interleukins, and prostaglandins [166]. Berköz et al. (2021) evaluated the protective effects of apigenin on LPS-induced liver damage. The flavonoid reduced liver damage markers and inhibited pro-inflammatory cytokines and the activation of the NF-κB pathway, while promoting the activity of antioxidative enzymes [167].

Zhang et al. (2021) investigated the role of luteolin in HMGB-1 release and the effects on the P2X7 receptor (P2X7R)/receptor for advanced glycation end products (RAGE)/TLR4 signaling process in hepatocyte damage in an experimental model of septic hepatic injury induced by LPS. As a result, the indicators of hepatic injury were inhibited by luteolin and reversed hepatic injury, especially inflammation, likely by regulating the release of HMGB-1 through the P2X7R/RAGE/TLR4 axis [168].

Furthermore, Wang et al. (2021) studied how luteolin ameliorates LPS-induced acute liver injury by inhibiting thioredoxin-interacting protein (TXNIP)/NLRP3 inflammasome in mice. As a result, luteolin inhibits cytokine production and oxidative stress and promotes antioxidant enzyme activity. Since the protective effect of luteolin was blocked by si-TXNIP in vitro, the TXNIP-NLPR3 axis could be a luteolin target [169].

Along with LPS, galactosamine is a hepatotoxin widely used in experimental research as a model to investigate mechanisms of liver damage and liver cell death. Galactosamine toxicity stems from its ability to directly interfere with cellular metabolism, specifically with uridine nucleotide stores in the liver, directly compromising RNA and protein synthesis. When administered together with LPS, galactosamine potentiates the inflammatory response through the activation of Kupffer cells (resident liver macrophages), cytokine release, and cell death [170].

Wu et al. (2024) researched quercetin effects on acute liver failure induced by D-galactosamine and LPS. Quercetin reduced excessive ROS, cell apoptosis, inflammatory responses, ALT and AST levels. Quercetin also promoted mitophagy by regulating the peroxisome proliferator-activated receptor gamma (PPARγ)/PPARγ coactivator-1 alpha (PGC-1α)/NF-κB axis and inhibiting apoptosis and inflammation mediated by mitochondrial dysfunction [171].

Zhao et al. (2021) analyzed the use of glycyrrhizin-mediated liver-targeted alginate nanogels to deliver quercetin for acute liver injury treatment. Quercetin-glycyrrhizin nanogels increased radical scavenging, demonstrated target efficiency to the liver, and restored levels of liver function markers. Histopathology showed an improvement in the hepatic damage caused by D-galactosamine/LPS, along with the downregulation of TNF-α, IL-6, iNOS, and monocyte chemoattractant protein-1 (MCP-1)/chemokine ligand 2 (CCL2) gene expression, as well as a decrease in MDA and GST levels [172]. Furthermore, Wei et al. (2021) studied the use of quercetin-loaded liposomes modified with galactosylated chitosan to prevent D-galactosamine/LPS induced acute liver injury. Treatment promoted M2 macrophage polarization, inhibited liver enzymes and lipid oxidation, and promoted antioxidant activity [173].

Feng et al. (2025) analyzed the efficacy of an innovative nanotherapy composed of ultrafine quercetin–iron nanoparticles (QFN) in the treatment of acute liver failure induced by D-galactosamide/LPS. As a result, it was observed that QFN blocks inflammatory activation in macrophages and cellular senescence, preventing inflammation from accelerating cellular aging, which in turn generates more inflammation (feedback cycle). Furthermore, the nanoparticles neutralize ROS, suppress apoptosis, and facilitate liver regeneration. Thus, these results demonstrate that QFN presents itself as a potential therapeutic agent for the treatment of acute liver failure and, possibly, against pathologies associated with inflammation and cellular senescence [174].

Finally, in an experimental model of acute liver failure induced by D-galactosamide/LPS, Tian et al. (2021) observed dose-dependent effects of kaempferol, whereas low doses of the flavonoid had autophagy-related hepatoprotective effects, the high doses had the opposite effect, inhibiting autophagy and worsening the liver damage. This work illustrates the importance of appropriate dosage, seen as the excessive administration of these plant-derived compounds may exert deleterious effects [175].

The scenario of liver damage by pathogens, their pathogenic components and related conditions has been widely explored as models to assess the activity of the selected flavonoids between 2020 and 2025. Some mechanisms are dependent on the experimental condition, such as inhibition of TLR4 signaling, which is activated by LPS, HMGB-1 carrying LPS and Gram-negative bacteria infections. Considering that cellular necrosis occurs in those models, the release of HMGB-1 and ATP as alarmins is a disease mechanism, and ATP leads to inflammasome activation and IL-1β maturation. Macrophage profile (e.g., inflammatory or repair) is also relevant in these scenarios. The selected flavonoids were active and targeted the essential disease mechanisms in the models, supporting their benefit and also potential pharmaceutical formulation towards their industrial development.

**Table 6:** Summary of the literature on selected flavonoids against pathogen- or pathogen-related-induced liver damage, from 2020 to 2025.

Pathogen-related or pathogen-induced liver damage				
AUTHORS	TREATMENTS	SUBJECTS	MODEL INDUCTION	MAIN FINDINGS
Sepsis-induced acute hepatic injury				
Deng et al. (2025) [164]	• Luteolin [0.2 mg/kg] • Intraperitoneal administration • 1 h after cecal ligation and puncture	• Male C57BL/6 mice and TLR4^−/−^ mice • 8–12-week-old	• Sepsis was induced by cecal ligation and puncture	Luteolin effects compared to stimulus group: • ↓ AST, ALT, MPO, and iNOS • ↓ IL-1β, IL-6, and TNF-α, • ↑ IL-10 and Arg1 • ↑ Macrophage polarization from proinflammatory M1 phenotype to an anti-inflammatory M2 phenotype • ↓ p-NF-κB (p-p65), TLR4, and MyD88 expression
Zearalenone-induced liver damage				
Rajendran et al. (2020) [163]	• Kaempferol [50 mg/kg] • Oral administration • For 3 weeks	• Albino male mice [25–30 g]	• Zearalenone [40 mg/kg] • Oral administration • For 14 days	Kaempferol effects compared to stimulus group: • ↓ ALT, AST, and ALP • ↓ H_2_O_2_, MDA, caspase-3, cleaved PARP, and Bcl-2 • ↓ p-Akt and p-PI3K • ↓ IL-6, IL-1β, and TNF-α • ↑ Nrf2 phosphorylation and HO-1, and NQO-1 expression • ↑ SOD, CAT, and GSH
Lipopolysaccharide-induced acute liver injury				
Berköz et al. (2021) [167]	• Apigenin [200 mg/kg] • Oral administration • For 7 days	• Adult male Balb/c mice [18–22 g] • 6–8-week-old	• Lipopolysaccharide [5 mg/kg] • Intraperitoneal administration • Day 7	Apigenin effects compared to stimulus group: • ↓ ALT, AST, ALP, γGT, CRP, MDA, total and direct bilirubin • ↑ GSH, SOD, and CAT • ↓ NOx, PGE_2_, TNF-α, IL-1β, IL-6, and MPO • ↓ iNOS and COX-2 mRNA • ↓ p-NF-κB p65, p-IκB, and p-IKK
Zhang et al. (2021) [168]	• Luteolin [60 mg/kg]	• Male C57BL/6 mice [18–20 g] • 8–10-week-old	• Lipopolysaccharide [60 mg/kg]	Luteolin effects compared to stimulus group: • ↓ Serum ALT and AST • ↓ Leukocyte infiltrate • ↓ IL-1β • ↓ HMGB-1 production and release • ↓ Caspase 1 activation
Wang et al. (2021) [169]	• Luteolin [100 mg/kg] • Oral administration • For 3 days	• Male C57BL/6 mice [20–25 g]	• Lipopolysaccharide [10 mg/kg] • Intraperitoneal administration • Day 4	Luteolin effects compared to stimulus group: • ↓ Oxidative stress and MDA levels • ↑ SOD and GSH levels • ↓ TNF-α, IL-10, and IL-6 • ↓ ASC, caspase-1, IL-1β, and IL-18
D-galactosamine/Lipopolysaccharide-induced acute liver injury				
Zhao et al. (2021) [172]	• Quercetin-glycyrrhizin nanogels [3.32 mg/kg] • Intravenous administration	• Male Kunming mice [18–22 g]	• D-galactosamine [700 mg/kg] Lipopolysaccharide [20 μg/kg] • Intraperitoneal administration	Quercetin-glycyrrhizin nanogels’ effects compared to stimulus group: • ↓ AST, ALT, and total bilirubin • ↓ TNF-α, IL-6, iNOS, and MCP-1 mRNA expression
Wei et al. (2021) [173]	• Quercetin-loaded liposomes modified with galactosylated chitosan [15 mL/kg] • Intravenous administration • For 6 days	• Male ICR mice [≈22 g]	• D-galactosamine [500 mg/kg] Lipopolysaccharide [20 μg/kg] • Intraperitoneal administration 1 h after the last treatment	Quercetin-loaded liposomes modified with galactosylated chitosan effects compared to stimulus group: • ↓ AST, ALT, ALP, LDH, and MDA • ↑ GSH and IL-10 • ↓ iNOS, TNF-α, and IL-1β (M1 markers) • ↑ CD206, CD163, and Arg1 (M2 markers)
Wu et al. (2024) [171]	• Quercetin [100 mg/kg] • Oral administration • For 7 days	• Male C57BL/6J mice [20–22 g] • 6–8-week-old	• D-galactosamine [300 mg/kg] Lipopolysaccharide [10 μg/kg] • Intraperitoneal administration	Quercetin effects compared to stimulus group: • ↓ AST and ALT • ↑ Bcl-2, PGC-1α, PPARγ • ↓ Cleaved-caspase-3 and p62 • ↓ PINK1, Perkin, Beclin-1, and LC3I/LC3II • ↓ IL-1β, IL-6, and TNF-α • ↓ p-IκB and p-NF-κB
Feng et al. (2025) [174]	• Ultra-small quercetin-based nanotherapeutics [10 mg/kg] • Intravenous administration 24 h before stimulus	• Male C57BL/6J mice [18–20 g] • 6–8-week-old	• D-galactosamine [300 mg/kg] Lipopolysaccharide [30 μg/kg] • Intraperitoneal administration	Ultra-small quercetin-based nanotherapeutics effects compared to stimulus group: • ↓ AST, ALT, and MDA • ↓ IL-1β, IL-6, IL-8, TNF-α, CDK1 and CDK2 • ↓ γH2AX, p16, p21, and p53
Tian et al. (2021) [175]	• Kaempferol [2.5, 5, 10, 20, or 40 mg/kg] • intravenous administration 2 h before stimulus	• Male C57BL/6 mice • 8–12-week-old	• D-galactosamine [700 mg/kg] Lipopolysaccharide [10 µg/kg] • Intraperitoneal administration	Dose-dependent Kaempferol effects compared to stimulus group: • Low doses [2.5–10 mg/kg]: ↓ AST, ALT, TNF-α, IL-6, IL-12, p40, IL-1β, CXCL2, CXCL10, p-JNK, p-ERK, p-p38, and induced autophagy • High doses [20–40 mg/kg]: opposite effects

Abbreviations: p-Akt (phosphorylated protein kinase B), Arg1 (arginase-1), ASC (apoptosis-associated speck-like protein containing a CARD), AST (aspartate aminotransferase), ALP (alkaline phosphatase), ALT (alanine aminotransferase), Bcl-2 (B-cell lymphoma 2), CAT (catalase), CD163 (cluster of differentiation 163), CD206 (cluster of differentiation 206), CDK1 (cyclin-dependent kinase 1), CDK2 (cyclin-dependent kinase 2), CRP (C-reactive protein), COX-2 (cyclooxygenase-2), CXCL2 (C-X-C motif chemokine ligand 2), CXCL10 (C-X-C motif chemokine ligand 10), γGT (gamma-glutamyl transferase), GSH (reduced glutathione), γH2AX (DNA double-strand breaks marker), H_2_O_2_ (hydrogen peroxyde), HMGB-1 (High Mobility Group Box 1), HO-1 (heme-oxygenase-1), IL-1β (interleukin-1 beta), IL-6 (interleukin-6), IL-8 (interleukin-8), IL-10 (interleukin-10), IL-12 p40 (p40 subunit of interleukin-12), IL-18 (interleukin-18), iNOS (inducible nitric oxide synthase), LC3I/LC3II (autophagy-related protein light chain 3 I and II), LDH (lactate dehydrogenase), MCP-1 (monocyte chemoattractant protein-1/CCL2), MDA (malondialdehyde), MPO (myeloperoxidase), MyD88 (myeloid differentiation primary response 88), NOx (nitrogen oxides), p-ERK (phosphorylated extracellular signal-regulated kinase), p-IκB-α (phosphorylated inhibitor of nuclear factor kappa B alpha)), p-IKK (phosphorylated IkB kinase), p-JNK (phosphorylated c-Jun N-terminal kinase), p-NF-κB/ p65 (phosphorylated nuclear factor kappa-light-chain-enhancer of activated B cells p65 subunit), PARP (Poly ADP-Ribose Polymerase), PGC-1α (peroxisome proliferator-activated receptor gamma coactivator 1-alpha), PGE2 (prostaglandin E2), PINK1 (PTEN-induced putative kinase 1), p-p38 (phosphorylated p38 mitogen-activated protein kinase (MAPK) protein), p-PI3K (phosphorylated phosphoinositide 3-kinase), PPARγ (Peroxisome Proliferator-Activated Receptor gamma), NQO1 (NAD(P)H:quinone oxidoreductase 1), Nrf2 (Nuclear factor erythroid 2-related factor 2), SOD (superoxide dismutase), TLR4 (toll-like receptor 4), TNF-α (tumor necrosis factor-alpha), ↑ (increase), ↓ (decrease).

#### 5.1.7. Intensive Sports-Induced Liver Inflammatory Injury

Studies show that, although regular physical exercise is a protective factor against inflammation, intense exercise can induce metabolic disorders. In this context, the liver (an important organ of energy metabolism) becomes vulnerable to energy depletion and oxidative stress, with possible activation of the NF-κB pathway [176]. Gao et al. (2020) observed the protective effects of quercetin against liver inflammation induced by intensive exercise (Table 7). As a result, they observed that quercetin administration improved hepatic function markers, inhibited oxidative stress and inflammation by regulating the activation of the NF-κB pathway [176]. Thus, the multitarget mechanism of flavonoids is supported in an additional disease condition.

#### 5.1.8. Cigarette Smoke-Induced Liver Inflammation

Smoking is considered one of the leading preventable causes of morbidity and mortality. Cigarette smoke is highly toxic and can promote increased production of reactive species and inflammatory response (with the release of TNF-α, IL-6, and IL-1β), leading to liver disease [177]. As a central organ in detoxification and immune response, the liver becomes especially vulnerable, causing oxidative, inflammatory, and apoptotic damage [175,177]. Unlike most inflammatory conditions, smoke-induced inflammatory damage is usually associated with increased levels of antioxidant enzymes, such as CAT, GPxI, metallothionein-I (MT-I), metallothionein-II (MT-II), SOD−I, SOD−II, and SOD-III [125]. Machado-Júnior et al. (2020) demonstrated that animals that received daily treatment with quercetin and were exposed to cigarette smoke showed a lessening of the recruitment of inflammatory cells, oxidative stress, and histopathological alterations in the liver (Table 7) [177]. In contrast, quercetin treatment increased the hepatic levels of CCL2 [177], a chemokine that chemoattracts CCR2^+^ monocytes/macrophages. Since CCR2^+^ monocytes/macrophages are essential for tissue repair [178,179], it is possible that quercetin exerted fine tuning, allowing for the restoration of the hepatic environment, although this aspect was not investigated.

#### 5.1.9. Cancer-Induced Hepatic Inflammation and Fibrosis

Table 7 summarizes the studies published between 2020 and 2025 that investigated selected flavonoids in protecting against cancer-induced hepatic inflammation and fibrosis. Gowda et al. (2023) investigated the effects of quercetin on hepatic inflammation and fibrosis due to breast cancer. Quercetin reduced tumor cell number, volume, body weight and liver weight, and peritoneal neoangiogenesis—observed by the decrease in peritoneal neoangiogenesis by CD31^+^ (endothelial cell) cells, liver enzymes, hepatic inflammation, and fibrosis. These findings indicate that quercetin’s antiangiogenic effects could account for the reduction in tumor growth and hepatic improvement [180].

Similarly, Abdu et al. (2022) demonstrated that quercetin administration reduced hepatocellular carcinoma growth, induced cell cycle arrest and apoptosis, as well as necrosis. Furthermore, the combined administration of quercetin with sorafenib (a multikinase inhibitor applied in cancer therapy) significantly improved liver injury and exhibited considerable antioxidant and antitumor effects [181]. Finally, Ahmed et al. (2022) demonstrated that the combined administration of the flavonoids quercetin and naringenin, in a rat hepatocellular carcinoma model induced by diethylnitrosamine/2-acetylaminofluorene, prevented the elevation of serum levels of liver function indicators and liver tumor biomarkers. In addition, the treatment suppressed cancerous histological lesions and inflammatory cell infiltration in the liver, decreased markers of hepatic oxidative stress, and increased antioxidant activity [182].

In principle, a reduction in inflammatory response would reduce the ability of the organism to battle against tumors [183]. However, the effect of flavonoids is more complex than that. In the context of hepatic cancer, flavonoids may target the tumor itself as well as the inflammation and fibrosis involved in the disease. This group of different actions, depending on the cellular targets, explains how flavonoids can reduce oxidative stress and inflammation while reducing tumor growth.

**Table 7:** Summary of the literature on selected flavonoids against acute liver damage induced by intensive sports, cigarette smoke or cancer, from 2020 to 2025.

Intensive sports-induced liver inflammatory injury				
AUTHORS	TREATMENTS	SUBJECTS	MODEL INDUCTION	MAIN FINDINGS
Gao et al. (2020) [176]	• Quercetin [100 mg/kg] • Oral administration • For 4 weeks	• Male BALB/c mice [18 ± 1 g] • 8 weeks old	• Intensive exercise protocol: treadmill running protocol of 28 m/min, 5° slope, 90 min/day concurrently for 7 consecutive days • 4th week of treatment	Quercetin effects compared to stimulus group: • ↓ AST and ALT • ↓ TNF-α, IL-1β, and IL-6 • ↓ iNOS, COX-2, and ICAM-1 mRNA • ↓ IKKα and p-IκB-α • ↑ IκB-α
Cigarette smoke-induced liver inflammation				
Machado-Junior et al. (2020) [177]	• Quercetin [10 mg/kg/day] • Oral administration 1 h before the smoke exposure	• Male C57BL/6 mice • 12 weeks old	• Smoking chamber: 12 cigarettes per day divided into three times daily (morning, afternoon, and night) for 60 consecutive days	Quercetin effects compared to stimulus group: • ↓ AST, ALT, LDH, γGT, IL-10, MDA, and SOD • ↑ CCL2 and IFN-γ
Cancer-induced hepatic inflammation and fibrosis				
Gowda et al. (2023) [180]	• Quercetin [50 mg/kg] • Intraperitoneal administration • For 10 days	• Female Swiss albino mice [18–21 g] • 5–6 weeks old	• Ascites fluid containing 1.5 million viable Ehrlich Ascites Carcinoma cells was administered via intraperitoneal 5 days before treatments	Quercetin effects compared to stimulus group: • ↓ ALP, AST, and ALT • ↓ tumor cell numbers, tumor volume, body weight, and liver weight • ↓ CD31 positive cells
Abdu et al. (2022) [181]	• Quercetin [50 mg/kg] • Intra-gastric administration	• Male Wistar albino-strain rats [160–200 g] • 10–12 weeks	• Diethylnitrosamine [200 mg/kg] • Intraperitoneal administration • Single injection • 2-acetylaminofluorene [30 mg/kg] • Intra-gastric doses • For 4 weeks	Quercetin effects compared to stimulus group: • ↓ ALT, AST, ALP, TP, and conjugated bilirubin • ↓ lipid concentrations • ↓ CRP, IL-6, MDA, and LDH • ↑ GSH • ↓ ki-67-expressing hepatocytes, AFP and PIVKA-II • ↓ Liver tissue damage, tumor nodules, and tumor cells sheets • ↓ Inflammation, angiogenesis, and cytoplasm basophilic • ↑ Apoptosis and necrosis • ↓ *Nfkb*, *Vegf* and *p53* genes
Ahmed et al. (2022) [182]	• Quercetin and Naringenin [10 mg/kg] • Oral administration • For 20 weeks	• Male Wistar rats [120–140 g]	• Diethylnitrosamine [150 mg/kg] • Intraperitoneal administration • For 2 weeks • 2-acetylaminofluorene [20 mg/kg] • Oral administration • For 3 weeks	Quercetin/Naringenin effects compared to stimulus group: • ↓ ALT, AST, ALP, γGT, total bilirubin, and albumin • ↓ AFP, CEA, and CA19.9 • ↓ Cancerous histological lesions and inflammatory cells infiltration • ↓ NO level and lipid peroxidation • ↑ SOD, GPx, and CAT • ↑ mRNA expression of liver IL-4, p53, and Bcl-2

Abbreviations: AFP (alpha-fetoprotein), ALP (alkaline phosphatase), ALT (alanine aminotransferase), AST (aspartate aminotransferase), Bcl-2 (B-cell lymphoma 2), CA19.9 (an antigen released by pancreatic cancer cells), CAT (catalase), CCL2 (chemokine C-C motif ligand 2), CD31 (cluster of differentiation 31), CEA (carcinoembryonic antigen), COX-2 (cyclooxygenase-2), CRP (c-reactive protein), GPx (Glutathione peroxidase), GSH (reduced glutathione), ICAM-1 (intercellular adhesion molecule-1), IFN-γ (interferon-gamma), IKKα (IκB kinase-alpha), IL-10 (interleukin-10), IL-1β (interleukin-1 beta), IL-4 (interleukin-4), IL-6 (interleukin-6), iNOS (inducible nitric oxide synthase), IκB-α (nuclear factor of kappa light polypeptide gene enhancer in B-cells inhibitor alpha), Ki-67 (proliferation marker), LDH (lactate dehydrogenase), MDA (malondialdehyde), NF-κB (nuclear factor kappa-light-chain-enhancer of activated B cells), NO (nitric oxide), p-IκB-α (phosphorylated nuclear factor of kappa light polypeptide gene enhancer in B-cells inhibitor alpha), PIVKA-II (protein induced by vitamin K absence or antagonist-II), SOD (superoxide dismutase), TNF-α (tumor necrosis factor-alpha), TP (total protein), VEGF (vascular endothelial growth factor), γGT (gamma-glutamyl transferase), ↑ (increase), ↓ (decrease).

Taken together, studies indicate that selected flavonoids exert consistent hepatoprotective effects in different experimental models of liver injury. Despite the heterogeneity of these models, their protective effects mainly converge on the reduction in oxidative stress, inhibition of NF-κB-mediated inflammation, activation of antioxidant pathways such as Nrf2/HO-1, attenuation of cell death, inflammasome activation, and preservation of liver structure and function. However, most of the evidence remains preclinical and varies considerably in terms of flavonoid type, dose, formulation, and treatment protocol, limiting direct translation to clinical practice. Therefore, although flavonoids represent promising multi-target compounds for liver protection, more standardized mechanistic and translational studies are needed to define their safety, bioavailability, therapeutic window, and potential interactions with conventional treatments.

### 5.2. Neuroprotective Effects of Flavonoids

**Figure 9:**
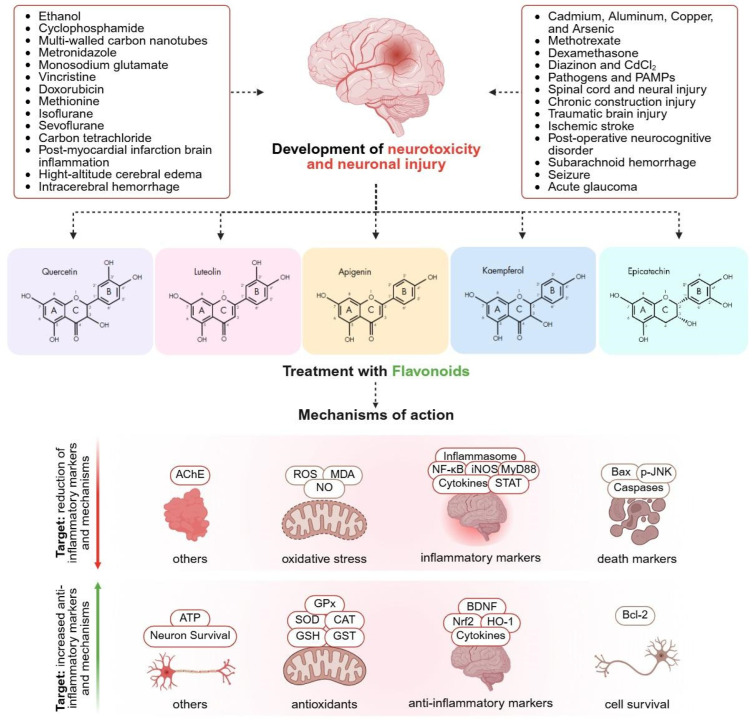
Shared mechanisms of action among flavonoids for the reduction in neurotoxicity and neural injury. Treatment with flavonoids, such as quercetin, luteolin, apigenin, kaempferol, and Epicatechin, acts on the modulation of multiple cellular and molecular mechanisms. These compounds promote the reduction in markers of neurotoxicity and neural injury (AChE), oxidative stress (ROS, MDA, NO), inflammation (NF-κB, iNOS, cytokines, inflammasome, MyD88, and STAT), and cell death (Bax, p-JNK, and caspases). Simultaneously, they stimulate endogenous antioxidant systems (SOD, CAT, GPx, GSH, and GST), anti-inflammatory pathways (Nrf2, BDNF, HO-1, and cytokines), neuron survival and cell survival mechanisms (Bcl-2). The acronyms mentioned are described in the “Abbreviations” section at the end of the article. Created in BioRender. Verri Jr, W. A. (2026) https://BioRender.com/krbxie3 (accessed on 5 June 2026).

In order to organize the discussion on the neuroprotective potential of flavonoids, the present section is divided into two major pathological contexts: neurotoxicity and neural injury. These categories were selected because they encompass key mechanisms underlying neuronal inflammation and oxidative stress, conditions in which flavonoids have demonstrated substantial protective activity. Neurotoxicity refers to cellular and molecular alterations triggered by chemical, metabolic, or inflammatory stimuli that impair neuronal homeostasis and activate glial responses [184,185], whereas neural injury involves ischemic, traumatic, or hemorrhagic insults characterized by acute inflammation and oxidative damage [186,187]. This framework allows for a comprehensive analysis of how flavonoids modulate inflammatory signaling pathways, redox balance, and neuronal survival, thereby highlighting their therapeutic relevance in non-degenerative neurological conditions. Figure 9 summarizes the agents that cause neurotoxicity and neural injury, and the mechanisms of action shared among the flavonoids mentioned in this article.

#### 5.2.1. Neurotoxicity

##### Drug-Induced Neurotoxicity

Table 8 summarizes the studies published between 2020 and 2025 that investigated selected flavonoids in protecting against drug-induced neurotoxicity. Quercetin has shown consistent neuroprotective effects across diverse models of chemically and biologically induced neurotoxicity, acting mainly through the suppression of oxidative stress, neuroinflammation, and apoptosis. In ethanol-related brain injury models, quercetin markedly attenuated mitochondrial dysfunction and neuronal apoptosis by downregulating the JNK/p38 MAPK signaling pathway, thereby reducing oxidative damage and inflammatory mediators, while enhancing antioxidant defense and antiapoptotic markers [188,189].

In toxin-induced neurotoxicity, quercetin consistently reversed oxidative and inflammatory insults. Against metronidazole, cyclophosphamide, carbon tetrachloride, and multi-walled carbon nanotube exposure, quercetin reduced lipid peroxidation and inflammatory cytokines, while restoring redox homeostasis and modulating key antioxidant transcriptional regulators [133,190,191,192]. Further, quercetin attenuated neuroinflammation and ferroptosis in sepsis-associated encephalopathy through suppression of the chemokine (C-X-C motif) ligand 2 (CXCL2)/C-X-C chemokine receptors 2 (CXCR2) axis and reduction in iron-dependent oxidative injury [193]. It also prevented cognitive deficits following isoflurane anesthesia by modulating the miR-138-5p/lipocalin 2 (LCN2) pathway, thereby suppressing hippocampal inflammation [194]. Similarly, in vincristine- and doxorubicin-induced neurotoxicity, quercetin preserved neuronal structure and function by activating the Nrf2/HO-1 and PI3K/Akt/Forkhead box protein O1 (FOXO1A) pathways and inhibiting apoptotic and ER stress markers [195,196].

In chemically induced neurotoxicity models, luteolin consistently mitigated oxidative stress, inflammation, and neuronal apoptosis. In doxorubicin-induced neurotoxicity, oral treatment with 50 or 100 mg/kg reduced levels of MDA, pro-inflammatory cytokines (e.g., IL-1β, IL-6, and TNF-α), and NF-κB, while increasing SOD, CAT, and GSH activities, leading to reduced apoptosis and improved memory performance [197]. In models of metal-induced neurotoxicity, treatment with luteolin reversed weight loss, motor retardation, multiple organ damage, and cognitive deficits induced by cadmium. Mechanistically, the treatment decreased the production of pro-inflammatory factors and neuroinflammation by inhibiting the Notch1/hairy and enhancer of split 1 (Hes1) inflammatory signaling axis and restoring BDNF-TrkB/Akt signaling [54]. An anti-inflammatory activity was also observed in sevoflurane-induced neurotoxicity, where luteolin inhibited neuronal apoptosis, neuroinflammation, and cognitive impairment in mice—effects achieved through increased HO-1 expression and activation of the autophagic pathway [198].

Apigenin exhibits consistent neuroprotective effects across various models of neurotoxicity in rodents. In rats exposed to monosodium glutamate, apigenin attenuated cortical oxidative and inflammatory damage by decreasing MDA levels and increasing antioxidant enzymes, such as SOD, CAT, and GPx. The treatment also lowered the expression of inflammatory mediators, including IL-1β, TNF-α, NO, and iNOS, thereby restoring cortical cytoarchitecture and overall neuronal integrity [199]. Similarly, in an aluminum chloride–induced neurotoxicity model, apigenin reduced oxidative and nitrosative stress, downregulated acetylcholinesterase (AChE) activity, and decreased NO levels. Histological analyses revealed preservation of the Purkinje cell layer in the cerebellum, suggesting strong structural neuroprotection. These effects were associated with modulation of the c-AMP response element-binding protein (CREB)-BDNF pathway and inhibition of β-amyloid accumulation, mechanisms that may contribute to its cognitive benefits [200]. In methotrexate-treated rats, apigenin prevented hippocampal microglial activation and ameliorated cognitive impairment through regulation of the microRNA 15a (miR-15a)/Rho-associated coiled-coil kinase 1 (ROCK-1)/ERK1-2/CREB/BDNF signaling axis, resulting in decreased neuroinflammation, oxidative stress, and apoptosis, as well as improved performance in behavioral tests [201].

Complementarily, Epicatechin demonstrated combined antioxidant and anti-inflammatory activity in arsenic-induced neurotoxicity in mice, restoring redox homeostasis through Nrf2 upregulation and NF-κB suppression, mechanisms that jointly alleviate oxidative and inflammatory neuronal stress [202].

In cadmium-induced cortical injury, oral kaempferol significantly reduced lipid peroxidation and NO levels while restoring SOD, CAT, and GSH activity. It downregulated TNF-α, IL-1β, and iNOS and regulated apoptotic markers by increasing Bcl-2 and decreasing Bax and caspase-3 expression, confirming its role in protecting cortical neurons from oxidative and inflammatory stress [203]. Similar effects were observed in cadmium chloride-induced hippocampal damage, where kaempferol improved memory deficits and hippocampal architecture through activation of Sirtuin1 (SIRT1) and inhibition of poly ADP-ribose polymerase 1 (PARP1). This led to reduced ROS, reactive nitrogen species (RNS), and MDA; increased GSH and manganese superoxide dismutase (MnSOD) levels; and suppression of TNF-α, IL-6, Bax, and cleaved caspase-3, while enhancing Bcl-2 expression, indicating a comprehensive defense against cadmium neurotoxicity [204].

Therefore, the protective mechanisms of flavonoids against neurotoxicity involve antioxidant effects related to their chemical structure, but also the induction of endogenous antioxidant responses that depend on regulating gene expression. Anti-inflammatory mechanisms are also part of flavonoids’ neuroprotection by inhibition of cytokine release and NF-κB activation.

**Table 8:** Summary of the literature on selected flavonoids against drug-induced neurotoxicity, from 2020 to 2025.

Drug-induced neurotoxicity				
AUTHORS	TREATMENTS	SUBJECTS	MODEL INDUCTION	MAIN FINDINGS
Ethanol-induced neurotoxicity				
Zhang et al. (2025a) [188]	• Quercetin [25–100 mg/kg] • Oral administration 6 h after ethanol administration • For 12 weeks	• Male Sprague–Dawley rats [175–250 g] • 12 weeks old	• Ethanol [53% 15 mL/kg] • Oral administration • For 12 weeks	Quercetin effects compared to ethanol group: • ↓ MDA, IL-1β, TNF-α, IL-6, p-p38, and p-JNK • ↑ SOD, GSH, and Bcl-2 • ↓ Bax and caspase-3
Zhang et al. (2025b) [189]	• Quercetin [25–100 mg/kg] • Oral administration 6 h after ethanol administration • For 12 weeks	• Male Sprague-Dawley rats [175–250 g] • 12 weeks old	• Ethanol [53% 15 mL/kg] • Oral administration • For 12 weeks	Quercetin effects compared to ethanol group: • ↓ TNF-α, IL-6, IL-1β, p-JNK, p-p38, ROS, and apoptosis • ↑ Antioxidant status
Cyclophosphamide-induced neural toxicity				
Onaolapo et al. (2023) [133]	• Quercetin supplemented died [100–200 mg/kg of feed] • For 3 weeks	• Male Wistar rats [120–150 g]	• Cyclophosphamide [150 mg/kg/day] • Intraperitoneal administration • On days 1 and 2	Quercetin effects compared to cyclophosphamide group: • ↓ MDA • ↑ AChE, GSH, SOD, CAT, Nrf2, and HO-1 • ↓ NO, IL-1β, TNF-α, NF-κB, NLRP3, and caspase-1
Multi-walled carbon nanotubes-induced neurotoxicity				
Sallam et al. (2022) [192]	• Quercetin [30 mg/kg/day] • Intraperitoneal administration • For 14 days	• Male Swiss albino mice [22–23 g] • 7–8 weeks old	• Multi-walled carbon nanotubes [0.25 and 0.5 mg/kg] • Intraperitoneal administration • Day one	Quercetin effects compared to carbon nanotubes group: • ↓ MDA and NO • ↑ GSH, SOD, and CAT • ↓ IL-1β, TNF-α, NF-κB, NLRP3, Caspase-1, and AChE
Metronidazole-induced neurotoxicity				
Chaturvedi et al. (2020) [190]	• Quercetin [50–100 mg/kg] • Oral administration • For 28 days	• Male Sprague–Dawley rats [200–220 g]	• Metronidazole [135 mg/kg] • Oral administration • For 28 days	Quercetin effects compared to metronidazole group: • ↓ MDA, ROS, and NO • ↓ IL-1β, IL-6, TNF-α, and iNOS • ↑ eNOS • ↓ Bax and Caspase-3 • ↑ Bcl-2
Monosodium glutamate induced neurotoxicity				
Albrakati et al. (2023) [199]	• Apigenin [20 mg/kg] • Oral administration • For 30 days	• Adult male Wister albino rats [150–200 g]	• Monosodium glutamate [4 mg/kg] • Oral administration	Apigenin effects compared to monosodium glutamate group: • ↓ MDA • ↑ SOD, CAT, GPx, and GR • ↓ IL-1β, TNF-α, NO, and iNOS
Vincristine-induced peripheral neuropathy				
Yardim et al. (2020) [195]	• Quercetin [25–50 mg/kg] • Oral administration	• Male Sprague–Dawley rats [220–250 g] • 10–12 weeks old	• Vincristine [0.1 mg/kg] • Intraperitoneal administration	Quercetin effects compared to vincristine group: • ↓ NF-κB and caspase-3 • ↓ ATF-6 and ER stress (PERK/IRE1/GRP78) • ↑ Nrf2, HO-1, NQO1, and Akt • ↑ Neuronal survival
Doxorubicin-induced neurotoxicity				
El-Shetry et al. (2024) [196]	• Quercetin [80 mg/kg] • Oral administration • For 14 days	• Male albino rats [200–250 g]	• Doxorubicin [2.5 mg/kg] • Intraperitoneal administration	Quercetin effects compared to doxorubicin group: • ↓ MDA • ↓ DNA damage • ↓ IL-1β • ↑ SOD, CAT, and GPx • ↑ Synaptophysin • ↓ Neuronal degeneration
Imosemi et al. (2021) [197]	• Luteolin [50 or 100 mg/kg] • Oral administration	• Mature male albino Wistar rats [160 ± 5 g]	• Doxorubicin [2 mg/kg] • Intraperitoneal administration	Luteolin effects compared to doxorubicin group: • ↓ MDA • ↓ IL-1β, IL-6, TNF-α, and NF-κB • ↑ SOD, CAT, GSH • ↓ Caspase-3 and Bax • ↑ Bcl-2
Isoflurane-induced postoperative cognitive dysfunction				
Lou et al. (2025) [194]	• Quercetin [5 mg/kg] • Intraperitoneal administration	• Male Sprague–Dawley rats [570–680 g] • 20 months old	• 3% Isoflurane inhalation	Quercetin effects compared to isoflurane group: • ↓ TNF-α, IL-1β, and IL-6 • ↑ miR-138-5p • ↓ LCN2 • ↑ cognitive performance
Sevoflurane-induced neurotoxicity				
Li et al. (2021) [198]	• Luteolin [30 mg/kg] • Intraperitoneal administration	• 1-day-old C57BL/6J mice and adult (7 weeks old) mice	• 3% Sevoflurane for 6 h	Luteolin effects compared to sevoflurane group: • ↓ Caspase-3, PARP, and NLRP-3 • ↓ Neuronal apoptosis and inflammation • ↑ LC3-I, LC3-II, and beclin-1 • ↑ Autophagy • ↑ HEMOX1 and HO-1
Carbon tetrachloride-induced oxidative neurotoxicity				
Zargar et al. (2021) [191]	• Quercetin [100 mg/kg] • Intraperitoneal administration • 2 h before carbon tetrachloride	• Male Wistar rats [80–90 g] • 8–12 weeks old	• Carbon tetrachloride [1 mL/kg] • Oral administration • Single dose	Quercetin effects compared to carbon tetrachloride group: • ↓ ALT, AST, urea, sodium, MDA, and ascorbic acid • ↑ GSH, CAT, and SOD • ↓ Apoptosis • ↑ Neuronal integrity
Cadmium-induced cortical injury				
Al-Brakati et al. (2021) [203]	• Kaempferol [50 mg/kg] • Oral administration • Daily for 30 days	• Adult male Sprague–Dawley rats [160–190 g] • 7–9 weeks old	• Cadmium [4.5 mg/kg] • Intraperitoneal administration	Kaempferol effects compared to cadmium group: • ↓ Cadmiun accumulation • ↑ AChE • ↓ LPO and NO • ↑ SOD, CAT, and GSH • ↓ TNF-α, IL-1β, and iNOS • ↑ Bcl-2 • ↓ Bax and caspase-3
Ma et al. (2025) [54]	• Luteolin [50 mg/kg] • Oral gavage • Daily for 14 days	• C57BL/6J male mice • 8 weeks old	• Cadmium [2 mg/kg] • Intraperitoneal administration	Luteolin effects compared to cadmium group: • ↓ Memory and learning deficits • ↓ Notch1/Hes1 • ↑ Motor behavior • Improved neuronal survival • ↓ Microglial and astrocytic activation • ↓ Glial inflammatory markers • ↑ BDNF, TrkB, and Akt
Aluminum chloride model				
Oyagbemi et al. (2025) [200]	• Apigenin [50 mg/kg] • Oral administration • For 14 days	• Male Wistar strain rats [100–120 g]	•Aluminum chloride model [100 mg/kg] • Oral administration • For 14 days	Apigenin effects compared to cadmium group: • ↓ MDA and H_2_O_2_ • ↓ NO and AChE • ↑ GSH and SOD
Methotrexate-induced neurotoxicity and cognitive impairment				
Taha et al. (2023) [201]	• Apigenin [20 mg/kg] • Oral administration • For 30 days	• Male Sprague Dawley rats [150–200 g] • 4–5 weeks old	• Methotrexate [50 mg] • Intravenous administration	Apigenin effects compared to methotrexate group: • ↑ miR-15a, p-ERK1/2, p-CREB, t-CREB, BDNF • ↑ GSH • ↓ ROCK-1, Iba-1, MDA • ↓ IL-1β and Caspase-3
Arsenic-induced neurotoxicity				
Shariati et al. (2024) [202]	• Epicatechin [25–100 mg/kg] • Oral administration • For 2 weeks	• Male NMRI mice [22–27 g]	• Arsenic [10 mg/kg] • Oral administration • For 5 weeks	Epicatechin effects compared to stimulus group: • ↓ LPO, NO, and TNF-α • ↑ Antioxidant defenses • ↓ NF-κB and Nrf2 • Restored redox balance
CdCl_2_-induced hippocampal damage				
El-Kott et al. (2020) [204]	• Kaempferol [50 mg/kg/day] • Oral administration • For 30 days	• Adult male Wistar rats [120 ± 5 g]	• CdCl_2_ [0.5 mg/kg] • Oral administration • For 30 days	Epicatechin effects compared to stimulus group: • ↑ SIRT1 activation and PARP1 • ↓ ROS/RNS and MDA • ↑ GSH and MnSOD • ↓ TNF-α and IL-6 • ↓ Bax, cleaved caspase-3, and Bcl-2 • ↓ NF-κB p65 and FOXO1

Abbreviations: AChE (acetylcholinesterase), Akt (protein kinase B), ALT (alanine aminotransferase), AST (aspartate aminotransferase), Bax (Bcl-2 Associated X protein), Bcl-2 (B-cell lymphoma 2), BDNF (brain-derived neurotrophic factor), CAT (catalase), eNOS (endothelial nitric oxide synthase), Epicatechin (epicatechin), ER (endoplasmic reticulum), FOXO1 (forkhead box protein O1), GPx (glutathione peroxidase), GR (glutathione reductase), ATF-6 (activating transcription factor-6), GRP78 (glucose-regulated protein 78), GSH (reduced glutathione), H_2_O_2_ (hydrogen peroxide), Hes1 (hairy and enhancer of split 1), HMGB1 (high mobility group box1), HO-1 (heme oxygenase-1), Iba-1 (ionized calcium-binding adapter molecule-1), IL-1β (interleukin-1 beta), IL-6 (interleukin-6), iNOS (inducible nitric oxide synthase), IRE1 (inositol-requiring enzyme-1), LCN2 (lipocalin-2), LPO (lipid peroxidation), MDA (malondialdehyde), MnSOD (manganese superoxide dismutase), NF-κB (nuclear factor of kappa light polypeptide gene enhancer in B-cells), NLRP3 (NOD-, LRR- and pyrin domain-containing protein 3), NO (nitric oxide), Notch1 (Neurogenic locus notch homolog protein 1), NQO1 (NAD(P)H quinone oxidoreductase 1), Nrf2(Nuclear factor erythroid 2-related factor 2), p-CREB (phosphorylated cyclic AMP response element-binding protein), p-ERK 1/2 (phosphorylated extracellular signal-regulated kinase 1/2), p-JNK (phosphorylated c-Jun N-terminal kinase), p-p38 (phosphorylated p38 mitogen-activated protein kinase), PARP1 (poly ADP-ribose polymerase-1), PERK (protein kinase R-like ER kinase), RNS (reactive nitrogen species), ROCK-1 (Rho-associated coiled-coil-containing protein kinase 1), ROS (reactive oxygen species), SIRT1 (sirtuin 1), SOD (superoxide dismutase), t-CREB (Total Cyclic AMP Response Element-Binding Protein), TNF-α (tumor necrosis factor-alpha), TrkB (tropomyosin receptor kinase B), ↑ (increase), ↓ (decrease).

##### PAMP- or Pathogen-Induced Neurotoxicity

Table 9 summarizes the studies published between 2020 and 2025 that investigated selected flavonoids in protecting against pathogen-related or pathogen-induced neurotoxicity. In LPS-induced models of neuroinflammation, depressive-like behavior and anxiety, quercetin improved behavioral and cognitive outcomes by inhibiting microglial activation and suppressing the NLRP3/NF-κB/iNOS and TLR4/MyD88 pathways, leading to reduced pro-inflammatory cytokines and increased antioxidant and neurotrophic signaling [205,206,207]. In inflammation-driven models, including rhinosinusitis and bacterial meningitis, quercetin preserved blood–brain barrier integrity and reduced systemic-to-central neuroinflammatory signaling by regulating PI3K/Akt/ERK activation and lowering cytokine and angiogenic factor levels [208,209]. Moving to a model of cecal ligation and puncture sepsis, quercetin reduced the production of the chemokine CXCL2, diminishing the activity and recruitment of CXCR2^+^ microglia as well as neuronal ferroptosis (a type of programmed cell death characterized by the requirement of iron and accumulation of membrane lipid peroxides) [193]. Collectively, these findings reinforce quercetin’s multi-target neuroprotective mechanism, integrating antioxidant, anti-inflammatory, and antiapoptotic mechanisms through the tight regulation of redox-sensitive and inflammatory pathways.

In an LPS-induced neuroinflammatory model in C57BL/6 mice, luteolin reduced levels of the cytokines IL-1β, TNF-α, and IL-6, as well as reducing the production of inflammatory mediators such as NO and iNOS. Mechanistically, these effects were modulated by less NF-κB activation and attenuation of microglial activation, reinforcing the dual antioxidant and anti-inflammatory activity of luteolin [210].

Likewise, in an LPS endotoxemia model, Epicatechin suppressed IL-6, IL-1β, and TNF-α expression while enhancing mitochondrial biogenesis and function, leading to preserved synaptic integrity and improved memory performance [211]. Finally, kaempferol exhibits consistent protection against a variety of neurotoxic insults. In a model of *Naja haje* venom-induced neurotoxicity, intraperitoneal administration of kaempferol markedly reduced mortality and restored hematological and antioxidant balance. This treatment lowered TNF-α, IL-6, MAO, and AChE levels while preventing histological brain damage, demonstrating strong antioxidant and anti-inflammatory effects that mitigated venom-induced neural toxicity [212].

Thus, flavonoids were neuroprotective in conditions ranging from local and systemic LPS exposure, intestinal lesion causing sepsis and even envenomation neurotoxicity, which supports their beneficial application.

**Table 9:** Summary of the literature on selected flavonoids against pathogen-related or pathogen-induced neurotoxicity, from 2020 to 2025.

Pathogen-related or pathogen-induced neurotoxicity				
AUTHORS	TREATMENTS	SUBJECTS	MODEL INDUCTION	MAIN FINDINGS
LPS-induced neurotoxicity				
Singh et al. (2022) [205]	• Quercetin [50–100 mg/kg] • Intraperitoneal administration • For 7 days	• Zebrafish [470–530 mg]	• LPS [1 mg/kg] • Intraperitoneal administration • Day 1	Quercetin effects compared to LPS group: • ↓ MDA, IL-1β, TNF-α, and nitrite • ↑ GSH • ↓ AChE
Adeoluwa et al. (2023) [206]	• Quercetin [50 mg/kg] • Oral administration • For 7 days	• Male rats • 17–18 weeks old	• LPS [0.85 mg/kg] • Intraperitoneal administration • Day 7	Quercetin effects compared to LPS group: • ↓ TNF-α, IL-6, IL-17, NLRP3, NF-κB, iNOS, and microglial activation • Induced behavioral recovery
Lee et al. (2020) [207]	• Quercetin [10–100 mg/kg] • Intraperitoneal administration • For 21 days	• Male Sprague–Dawley rats [210–230 g] • 6 weeks old	• LPS [2 μL/min for 5 min] • Intracerebroventricular administration into the lateral ventricle of the rat brain	Quercetin effects compared to LPS group: • ↓ IL-6, IL-1β, COX-2, NF-κB, and iNOS • ↑ BDNF
Tiboc-Schnell et al. (2020) [208]	• Quercetin [80 mg/kg] • Intranasal administration • For 7 days 2 h	• Female Wistar rats [100 ±10 g] • 20 days old	• LPS [5 or 10 µg] • Intranasal administration • Once a day for seven consecutive days	Quercetin effects compared to LPS group: • ↓ TNF-α, IL-6, IL-1β in nasal mucosa, lungs, and brain • ↓ Inflammatory exudate
Ling et al. (2022) [211]	• Epicatechin [50 mg/kg] • Administration after 0.5, 24 and 48 h	• Male C57BL/6 mice • 8 weeks old	• LPS [5 mg/kg]	Epicatechin effects compared to LPS group: • ↓ IL-6, IL-1β, and TNF-α • ↑ ATP, TOMM20, and mtDNA • ↑ PSD95 and synaptic density • Improved memory and neuronal survival
Zhou et al. (2021) [210]	• Luteolin [20 mg/kg] • Intraperitoneal administration	• C57BL/6 mice	• LPS [0.5 mg/kg] • Intraperitoneal administration	Luteolin effects compared to LPS group: • ↓ IL-1β, TNF-α, IL-6, and NF-κB • ↓ NO and iNOS • ↓ Microglial activation • ↑ Cognition and memory
Yang et al. (2024) [193]	• Quercetin [30–50 mg/kg] • Intraperitoneal administration	• Male C57BL/6J mice	• Sepsis-associated encephalopathy (CLP model)	Quercetin effects compared to LPS group: • ↓ IL-1β, IL-6, and TNF-α • ↓ CXCL2/CXCR2 • ↓ Ferroptosis • ↓ MDA and Fe^2+^ • ↑ GSH • ↑ Survival • ↑ Cognition
*Glaesserella parasuis*–induced meningitis				
Sun et al. (2024) [209]	• Quercetin [2.5–10 µg/mL]	• Female Kunming mice • 6-week-old	• *Glaesserella parasuis* [2 × 10^9^ CFU] • Intraperitoneal administration	Quercetin effects compared to *G. parasius* group: • ↓ IL-18, IL-6, IL-8, and TNF-α • ↓ MMP9, VEGF, ANG-2, and ET-1 • ↑ Sema4D and PlexinB1 • ↑ PI3K/Akt/ERK activation
*Naja haje* venom-induced neurotoxicity				
Ajisebiola et al. (2024) [212]	• Kaempferol [4 and 8 mg/kg] • Intraperitoneal administration • 30 min post-envenoming for 7 days	• Male albino Wistar rats	• *Naja haje* venom [0.2 mL]	Kaempferol effects compared to *Naja haje* venom group: • ↓ Mortality • ↓ TNF-α, IL-6, MAO, and AChE • ↑ GSH, SOD, CAT, and GST • ↓ MDA and nitrite • Prevention of histological brain damage

Abbreviations: AChE (acetylcholinesterase), Akt (protein kinase B), ANG2 (angiopoietin-2), ATP (adenosine triphosphate), BDNF (brain-derived neurotrophic factor), CAT (catalase), COX-2 (cyclooxygenase-2), CXCL2 (chemokine (C-X-C motif) ligand 2), CXCR2 (C-X-C motif chemokine receptor 2), ERK (extracellular signal-regulated kinase), ET-1 (endothelin-1), GSH (reduced glutathione), GST (glutathione S-transferase), IL-10 (interleukin-10), IL-17 (interleukin-17), IL-18 (interleukin-18), IL-1β (interleukin-1 beta), IL-6 (interleukin-6), IL-8 (interleukin-8), iNOS (inducible nitric oxide synthase), MAO (monoamine oxidase), MDA (malondialdehyde), MMP9 (matrix metalloproteinase-9), mtDNA (mitochondrial DNA), NF-κB (nuclear factor of kappa light polypeptide gene enhancer in B-cells), NLRP3 (NOD-, LRR- and pyrin domain-containing protein 3), PI3K (phosphoinositide 3-kinase), PSD95 (postsynaptic density protein 95), Sema4D (semaphorin 4D), SOD (superoxide dismutase), TNF-α (tumor necrosis factor-alpha), TOMM20 (translocase of outer mitochondrial membrane 20), VEGF (vascular endothelial growth factor), ↑ (increase), ↓ (decrease).

#### 5.2.2. Neuronal Injury/Lesion

Table 10 summarizes the studies published between 2020 and 2025 that investigated selected flavonoids in protecting against neuronal injury or lesion. Quercetin and its derivatives also exhibit robust neuroprotective potential across diverse neuronal injury paradigms involving ischemia, trauma, and hemorrhage. In models of brachial plexus avulsion and traumatic spinal cord injury, quercetin significantly promoted axonal regeneration, reduced oxidative stress, and suppressed inflammation, ultimately improving motor and sensory recovery [213,214,215]. Similarly, in ischemic stroke and global cerebral ischemia, quercetin-based formulations and senolytic treatment (Dasatinib + Quercetin) restored mitochondrial function, enhanced antioxidant defenses, and reduced neuronal apoptosis, indicating improved behavioral and cognitive outcomes through mitochondrial preservation and suppression of glial senescence [216,217].

In traumatic brain injury, quercetin mitigated microglial activation and cerebral edema through PGC-1α/Nrf2 pathway activation and Histone Deacetylase 3 (HDAC3) inhibition, while in subarachnoid hemorrhage and neonatal hypoxic–ischemic brain injury, both quercetin and dihydroquercetin improved neurological scores, reduced infarct volume, and limited ferroptosis and inflammation via PI3K/Akt/Nrf2/HO-1 and TLR4/MyD88/NF-κB signaling, respectively [218,219,220]. Collectively, these findings highlight quercetin’s broad efficacy in mitigating neuronal loss and functional impairment after ischemic, traumatic, and hemorrhagic injuries, primarily through modulation of oxidative stress, inflammation, and mitochondrial homeostasis. These findings indicate that quercetin exerts its neuroprotective and pro-resolving actions across both neurotoxic and injury-related conditions primarily through the activation of Nrf2/HO-1 signaling, inhibition of NF-κB-mediated inflammation, attenuation of oxidative and ferroptotic stress, and regulation of microglial phenotype [213,215,216,217,218,219,220]. This multifaceted activity highlights quercetin’s potential as a nutraceutical capable of preventing or ameliorating a wide spectrum of neuroinflammatory and neurodegenerative disorders.

In experimental models of brain injury, apigenin has also demonstrated notable anti-inflammatory and antioxidant actions. In a mild traumatic brain injury model in Wistar rats, treatment with apigenin immediately after trauma led to reduced oxidative stress, evidenced by decreased luminol/lucigenin levels, and enhanced anti-inflammatory cytokine IL-10 production. The compound mitigated cortical tissue damage and preserved structural integrity, although functional behavioral recovery was not evident in the short term. These findings support apigenin’s protective effects at molecular and histological levels and highlight its therapeutic potential as an adjunct in traumatic brain injury management [213].

Similarly, in traumatic brain injury rats, Epicatechin promoted a shift in microglial phenotype toward anti-inflammatory and reparative states, decreasing neuroinflammation and improving recovery through modulation of the AKT–p53/CREB signaling pathway [221]. In high-altitude cerebral edema, epicatechin gallate attenuated brain water content, blood–brain barrier disruption, and microglial activation via inhibition of NF-κB p65 phosphorylation, demonstrating anti-edematous and anti-inflammatory neuroprotection [222].

In structural injury models, kaempferol and its nano-formulations exert significant neuroprotective effects by attenuating oxidative damage, neuroinflammation, and apoptosis. In a neuropathic pain model induced by chronic constriction injury (CCI), oral kaempferol suppressed microglial activation by promoting a phenotypic shift from pro-inflammatory (M1) to anti-inflammatory (M2) states. It inhibited TLR4/NF-κB and MAPK signaling pathways, decreased IL-1β, TNF-α, and IL-6, and increased IL-10 levels, demonstrating potent anti-inflammatory and neuroprotective properties [223].

Kaempferol-loaded solid lipid nanoparticles (K-SLNs, 1 mg/kg, oral) improved drug delivery to the brain in a focal cerebral ischemia model, leading to decreased infarct volume and ROS generation, suppression of NF-κB and phosphorylated STAT3, and enhanced myelination and neurological recovery. These results highlight cationic solid lipid nanoparticles as an effective strategy to overcome blood–brain barrier limitations and potentiate kaempferol’s therapeutic efficacy in ischemic conditions [224]. In postoperative neurocognitive disorder (PND) models, intraperitoneal administration of kaempferol nanoparticles decreased TNF-α, IL-6, and IL-1β expression, reduced microglial activation, and prevented hippocampal oxidative and apoptotic damage, resulting in significant improvement of postoperative cognitive performance [225]. In a middle cerebral artery occlusion (MCAO) rat model of ischemic stroke, oral kaempferol reduced cerebral infarct size and edema, improved neurological scores, and suppressed apoptosis and neutrophil-mediated inflammation. Mechanistically, these effects were linked to activation of the BDNF–TrkB–PI3K/Akt pathway and inhibition of Janus kinase (JAK)/STAT3 signaling, consolidating kaempferol’s dual anti-apoptotic and anti-inflammatory neuroprotective action [226]. Collectively, the evidence demonstrates that kaempferol exerts robust neuroprotection across multiple experimental paradigms. Through modulation of redox balance, inflammatory mediators, and cell survival pathways, both free kaempferol and its nano-formulations contribute to the preservation of neuronal integrity and recovery from neurotoxic or injury-related insults.

In seizure-induced oxidative and inflammatory damage, luteolin administration significantly improves the severity, frequency, and duration of kainic acid-induced seizures. Mechanistically, luteolin preserves neuronal integrity, reduces hippocampal damage, and inhibits neuronal apoptosis and inflammation in mice—effects achieved through the prevention of MAPK and NF-κB signaling pathways and the reduction in oxidative stress [227]. Similarly, luteolin administration improved outcomes after intracerebral hemorrhage by reducing inflammation through modulation of the TLR4/TNF receptor-associated factor 6 (TRAF6)/NF-κB signaling pathway and pro-inflammatory cytokines such as TNF-α and IL-1β. Furthermore, the treatment provided neuroprotection, decreasing neuronal degeneration, cerebral edema, and lesion extent, directly contributing to a significant improvement in motor and sensory deficits [228]. In neuropathic pain induced by chronic constriction injury, luteolin reduced thermal allodynia and hyperalgesia, as well as mood disturbances presented by the animals. Furthermore, it increased levels of glial cell-derived neurotrophic factor (GDBF), BDNF, Bcl-2, SOD, CAT, and Nrf2 in the hippocampus and prefrontal cortex. Additionally, luteolin decreased levels of MDA, Bax, NF-κB, NLRP3, and pro-inflammatory cytokines such as IL-1β, IL-18, IL-6, and TNF-α [229]. Finally, protective effects were also observed in subarachnoid hemorrhage, where luteolin significantly inhibited neuroinflammation by reducing microglial activation, neutrophilic infiltrates, and the release of pro-inflammatory cytokines (through reduced NLRP3 activation). Furthermore, the treatment increased antioxidant systems (e.g., Nrf2) and consequently improved damage caused by oxidative stress [230].

Thus, neuronal injury is a field in which flavonoids have been explored in recent years. Each study may apply different approaches depending on the research group’s expertise and identified disease mechanisms. Overall, the flavonoids presented antioxidant and anti-inflammatory mechanisms that were sufficient to interfere with cell survival, cellular recruitment and function, and disease parameters that were relevant to propose them as treatments. There is also ongoing pharmaceutical development for the controlled release of flavonoids.

**Table 10:** Summary of the literature on selected flavonoids against neuronal injury or lesion, from 2020 to 2025.

Injury-induced neuro-damage				
AUTHORS	TREATMENTS	SUBJECTS	MODEL INDUCTION	MAIN FINDINGS
Brachial plexus avulsion (neural trauma)				
Huang et al. (2020) [213]	• Quercetin-loaded PLGA–PEG–PLGA hydrogel [5–100 mg/mL]	• Female Sprague–Dawley rats [200–250 g] • 10–12 weeks old	• Brachial plexus avulsion	Quercetin effects compared to stimulus group: • ↓ ROS • ↓ IL-6 • ↑ Motor neuron survival • ↑ Muscle fiber regeneration • ↑ Motor function recovery
Spinal cord injury				
Yang et al. (2024) [214]	• Quercetin-loaded small extracellular vesicles [sEVs-Que; 5 mg/kg] • Intravenous administration	• Female Sprague–Dawley rats	• Spinal cord injury	Quercetin effects compared to stimulus group: • ↓ TNF and IL-1α • ↓ C1q and iNOS • ↓ GFAP/C3 • ↑ JAK2/STAT3 signaling • ↑ Axonal growth • ↑ Motor recovery
Keyhanifard et al. (2023) [215]	• Quercetin + Hyperbaric Oxygen Therapy [100 mg/kg] • Intraperitoneal administration	• Male Sprague–Dawley rats (200–250 g)	• Traumatic spinal cord injury	Quercetin effects compared to stimulus group: • ↓ TNF-α and IL-1β • ↓ MDA • ↑ IL-10 • ↑ GSH, SOD, and CAT • ↓ Caspase-3 • ↑ Neuron survival
Chronic constriction injury				
Chang et al. (2022) [223]	• Kaempferol [60 mg/kg] • Oral administration • Daily for 21 days	• Adult male Sprague–Dawley rats [220–280 g] • 8 weeks old	• Chronic constriction injury of the right sciatic nerve	Kaempferol effects compared to stimulus group: • ↓ Microglial activation • M1 → M2 polarization • ↓ TLR4/NF-κB and MAPK • ↓ IL-1β, TNF-α, and IL-6 • ↑ IL-10
Mokhtari et al. (2023) [229]	• Luteolin [10, 20, and 50 mg/kg] • Oral gavage • For 21 days	• Adult male Sprague-Dawley rats (200–220 g)	• Chronic constriction injury	Luteolin effects compared to stimulus group: • ↓ Thermal allodynia and hyperalgesia • ↓ Mood disturbances • ↑ GDBF, BDNF, Bcl-2, SOD, CAT, and Nrf2 • ↓ MDA, Bax, NF-κB, and NLRP3 • ↓IL-1β, IL-18, TNF-α, and IL-6
Traumatic brain injury				
Zhai et al. (2024) [218]	• Quercetin [50 mg/kg] • Intraperitoneal administration	• ICR male mice [28–32 g] • 6–8 weeks old	• Traumatic brain injury (weight-drop model)	Quercetin effects compared to stimulus group: • ↓ TNF-α, IL-1β, and IL-6 • ↓ Apoptosis • ↓ Cerebral edema • ↑ PGC-1α/Nrf2 • ↓ HDAC3
Kuru Bektaşoğlu et al. (2023) [231]	• Apigenin [20 and 40 mg/kg]	• Wistar albino male rats	• Mild traumatic brain injury model	Apigenin effects compared to stimulus group: • ↓ Oxidative stress • ↓ Cortical damage • ↑ IL-10
Wang et al. (2024) [221]	• Epicatechin [0.5–2 mg/kg] • Oral administration • Daily for 7 days	• Adult male Sprague-Dawley rats	• Neuroinflammation after traumatic brain injury	Epicatechin effects compared to stimulus group: • ↓ Microglial activation (CD86, iNOS) • ↑ CD206 and Arg1 • ↓ p53 • ↑ AKT and CREB phosphorylation • Improved neurological recovery
Ischemic stroke				
Jian et al. (2025) [216]	• Quercetin-based polydopamine nanoparticles • Intravenous administration	• MCAO rats	• Ischemic stroke	Quercetin-based polydopamine nanoparticles effects compared to stimulus group: • ↓ MDA • ↓ Caspase-3 • ↓ LC3 • ↓ CD4/CD11b • ↑ M2 microglia • ↑ Behavioral recovery
Zhang et al. (2022) [226]	• Kaempferol [25–100 mg/kg] • Oral administration	•Adult male Sprague-Dawley rats [280–300 g] • 8 weeks old	• Ischemic stroke	Kaempferol effects compared to stimulus group: • ↓ Infarct volume and edema • ↑ BDNF–TrkB–PI3K/AKT pathway • ↓ Neutrophil infiltration • ↓ JAK1/STAT3 signaling • Improved neurological recovery
Gu et al. (2025) [217]	• Dasatinib + Quercetin (50 mg/kg 3×/week, oral)	• C57BL/6J mice • 3 months old	• Global ischemic brain injury	Quercetin effects compared to stimulus group: • ↓ ROS • ↓ SASP factors • ↓ Glial senescence • ↑ SOD2 • ↑ Mitochondrial integrity • ↑ ATP • ↑ Memory performance
Ghosh et al. (2024) [224]	• Kaempferol-loaded solid lipid nanoparticles [1 mg/kg] • Oral administration	• Male Wistar rats	• Focal cerebral ischemia	Kaempferol effects compared to stimulus group: • ↑ Brain bioavailability • ↓ ROS, NF-κB, and p-STAT3 • ↓ Infarct volume • Improved myelination and neurological outcomes
Le et al. (2020) [220]	• Quercetin [50 mg/kg i.p.]	• Neonatal mice (Rice-Vannucci method)	• Hypoxic–ischemic brain injury	Quercetin effects compared to stimulus group: • ↓ Infarct volume • ↓ Oxidative stress • ↓ TNF-α and IL-1β • ↓ TLR4/MyD88/NF-κB • ↑ Motor and cognitive recovery
Postoperative neurocognitive disorder				
Huang et al. (2024) [225]	• Kaempferol nanoparticles [10 mg/kg] • Intraperitoneal administration • For 7 days	• C57BL/6 mice	• Postoperative neurocognitive disorder	Kaempferol effects compared to stimulus group: • ↓ TNF-α, IL-6, and IL-1β • ↓ Microglial activation • ↓ Oxidative stress and apoptosis • Improved cognitive function
Intracerebral hemorrhage				
Yang et al. (2020) [228]	• Luteolin [5, 10 and 20 mg/kg] • Intraperitoneal administration	• Adult male Sprague Dawley rats [250–300 g]	• Intracerebral hemorrhage	Luteolin effects compared to stimulus group: • ↓ TNF-α and IL-1β • ↓ TLR4/TRAF6/NF-κB • ↓ Neuronal degeneration, cerebral edem, and lesion extend • ↓ Motor and sensory deficits • Provided neuroprotection
Subarachnoid hemorrhage				
Zheng et al. (2025) [219]	• Dihydroquercetin • Oral administration	• Rats	• Subarachnoid hemorrhage	Dihydroquercetin effects compared to stimulus group: • ↓ Brain edema • ↓ Ferroptosis • ↓ Inflammation • ↑ PI3K/AKT/Nrf2/HO-1 activation • ↑ Neurological recovery
Zhang et al. (2021) [230]	• Luteolin [10, 30, 60, and 90 mg/kg]	• Adult male Sprague Dawley rats [250–300 g]	• Subarachnoid hemorrhage	Luteolin effects compared to stimulus group: • ↓ Inflammatory cytokines • ↓ Microglial activation and neutrophilic infiltrates • ↑ Nrf2 • ↓ Oxidative stress
Seizure-induced neurotoxicity				
Luo et al. (2025) [227]	• Luteolin	• Murine model	• Kainic acid [10 mg/kg]	Luteolin effects compared to stimulus group: • ↓ Severity, frequency, and duration of epicatechinleptic seizures • ↓ Hippocampal damage, neuronal apoptosis, and inflammation • ↓ MAPK and NF-κB • Preserves neuronal integrity

Abbreviations: Akt (protein kinase B), Arg-1 (arginase-1), ATP (adenosine triphosphate), Bax (Bcl-2 Associated X protein), BDNF (brain-derived neurotrophic factor), C1q (complement component 1q), C3 (complement 3), CAT (catalase), CD11b (cluster of differentiation 11b), CD206 (cluster of differentiation 206), CD4 (cluster of differentiation 4), CD86 (cluster of differentiation 86), CREB (cAMP response element-binding protein), GFAP (glial fibrillary acidic protein), GSH (reduced glutathione), HDAC3 (histone deacetylase 3), HO-1 (heme oxygenase-1), IL-10 (interleukin-10), IL-1α (interleukin-1 alpha), IL-1β (interleukin-1 beta), IL-6 (interleukin-6), iNOS (inducible nitric oxide synthase), JAK1 (Janus kinase 1), JAK2 (Janus kinase 2), LC3 Microtubule-associated protein 1A/1B-light chain 3, LPO (lipid peroxidation), MAPK (mitogen-activated protein kinase), MDA (malondialdehyde), MMP9 (matrix metalloproteinase-9), MyD88 (myeloid differentiation primary response 88), NF-κB (nuclear factor of kappa light polypeptide gene enhancer in B-cells), NO (nitric oxide), Nrf2 (Nuclear factor erythroid 2-related factor 2), p-STAT3 (phosphorylated signal transducer and activator of transcription 3), PGC1α (peroxisome proliferator-activated receptor gamma coactivator 1-alpha), PI3K (phosphoinositide 3-kinase), ROS (reactive oxygen species), SASP (senescence-associated secretory phenotype), SOD (superoxide dismutase), SOD (superoxide dismutase), SOD2 (superoxide dismutase-2), STAT3 (signal transducer and activator of transcription 3), STAT3 (signal transducer and activator of transcription factor 3), TLR4 (toll-like receptor 4), TNF-α (tumor necrosis factor-alpha), TrKB (tropomyosin receptor kinase B), ↑ (increase), ↓ (decrease).

Taken together, studies show that selected flavonoids exert broad neuroprotective effects in different experimental models of neurotoxicity and neural injury. These findings corroborate the concept that flavonoids act as multi-target compounds capable of modulating central mechanisms shared by neurotoxic, inflammatory and injury-related disorders. However, similar to the studies in Section 5, most of the evidence remains preclinical and heterogeneous with respect to experimental models, doses, formulations, timing of treatment, and outcome measures. Therefore, while data indicate flavonoids and their nanoformulations are promising as neuroprotective strategies, translational studies are needed to define their cerebral bioavailability, penetration of the blood–brain barrier, therapeutic window, long-term safety, and potential interactions with conventional treatments.

### 5.3. Flavonoid Effects Against Lung Damage and Inflammation

**Figure 10:**
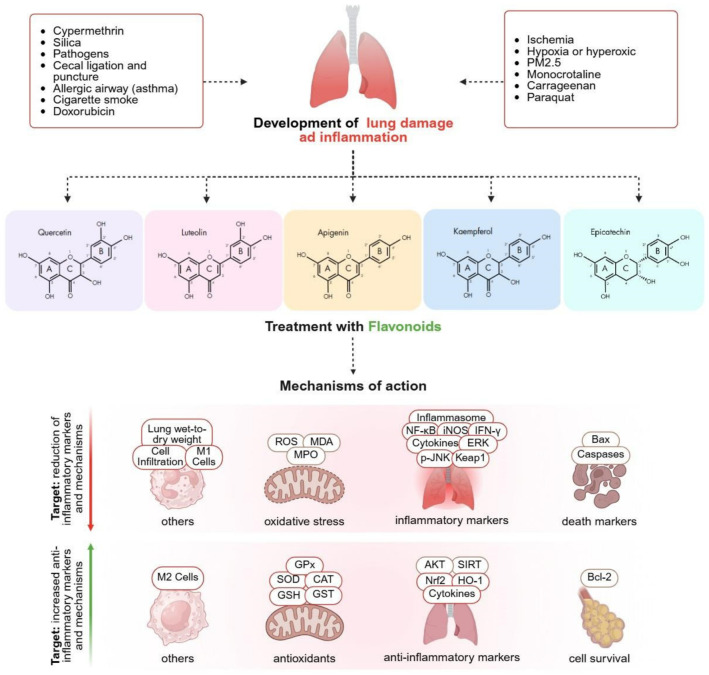
Shared mechanisms of action among flavonoids for the reduction in lung damage and inflammation. Treatment with flavonoids, such as quercetin, luteolin, apigenin, kaempferol, and epicatechin, acts on the modulation of multiple cellular and molecular mechanisms. These compounds promote the reduction in markers of lung damage (lung wet-to-dry weight, cell infiltration, and M1 cells), oxidative stress (ROS, MDA, MPO), inflammation (NF-κB, iNOS, cytokines, inflammasome, IFN, ERK, and Keap1), and cell death (Bax and caspases). Simultaneously, they stimulate endogenous antioxidant systems (SOD, CAT, GPx, GSH, and GST), anti-inflammatory pathways (AKT, SIRT, Nrf2, HO-2, and cytokines), neuron survival and cell survival mechanisms (Bcl-2). The acronyms mentioned are described in the “Abbreviations” section at the end of the article. Created in BioRender. Verri Jr, W. A. (2026) https://BioRender.com/krbxie3 (accessed on 5 June 2026).

The respiratory system is composed of the nose, oropharynx, larynx, trachea, bronchi, bronchioles, and lungs [232]. Of these, the lungs are essential organs of the respiratory system, whose main function is to promote gas exchange between the external environment and the bloodstream. In the alveoli, O_2_ is transported to the capillary network and then carried by the bloodstream to perfuse the tissues [232]. In general, acute or chronic lung diseases often present mechanisms based on the development of oxidative stress and the production of pro-inflammatory mediators, compromising lung function and the structural integrity of the tissue [233]. In this context, flavonoids become promising candidates for the prevention and treatment of lung diseases due to their antioxidant and anti-inflammatory properties. Below, we discuss the use of the flavonoids chosen here in different models of lung damage and/or inflammation. Table 11 summarizes the main studies described below. Figure 10 summarizes the agents that cause lung damage and inflammation and the mechanisms of action shared among the flavonoids mentioned in this article.

#### 5.3.1. Chemically or Physically Induced Lung Toxicity

Acute lung injury and its severe form, acute respiratory distress syndrome, represent a major clinical challenge due to their high morbidity and mortality rates. Characterized by uncontrolled pulmonary inflammation, acute lung injury can be triggered by severe sepsis, bacterial pneumonia, trauma, and burns [234]. Pathologically, the condition involves extensive diffuse alveolar damage, marked by inflammation, increased production of proinflammatory mediators, and apoptosis of alveolar epithelial cells, leading to the accumulation of inflammatory edema fluid, which compromises gas exchange [235]. Therefore, restoring alveolar fluid clearance is a critical therapeutic goal since effective fluid removal is associated with reduced mortality and a shorter duration of mechanical ventilation. Despite the use of lung-protective ventilation, a lack of targeted pharmacological therapies contributes to the persistently high mortality from this condition [236,237,238].

##### Cypermethrin-Induced Lung Toxicity

Ileriturk et al. (2022) investigated the effects of quercetin against lung toxicity induced by the insecticide cypermethrin, a type II synthetic pyrethroid that acts as a neurotoxin to insects by delaying the closure of voltage-sensitive sodium channels [239]. Pulmonary damage was induced by prolonged cypermethrin oral administration and confirmed by increased oxidative stress, inflammation, apoptosis, and autophagy in rats’ pulmonary tissues. The group that received co-treatment with quercetin showed decreased oxidative stress, inflammation suppression, and apoptosis inhibition. Quercetin also blocked the activation of the IL-6/JAK2/STAT3/MAPK14 pathway and autophagy, indicating comprehensive protection against cypermethrin-induced lung injury by quercetin and highlighting its potential as a therapeutic agent for managing insecticide toxicity [239].

##### Doxorubicin-Induced Lung Damage

As previously seen, doxorubicin is an antineoplastic drug also used in lung cancer treatment. However, it presents several side effects linked to increased oxidative stress, inflammation, and apoptosis. Owumi et al. (2021) evaluated the effect of luteolin on doxorubicin-induced lung dysfunction [240]. The results showed that luteolin increased survival and decreased oxidative stress in the lungs of treated animals. Furthermore, the treatment reduced levels of pro-inflammatory cytokines and apoptotic biomarkers and increased red blood cell, white blood cell, and platelet counts, thus attenuating pathological lung lesions. These findings suggest that supplementing patients undergoing doxorubicin chemotherapy with flavonoids, specifically luteolin, may prevent the onset of undesirable side effects linked to oxidative stress and toxicity [240].

##### Monocrotaline-Induced Pulmonary Hypertension

Elevated pulmonary arterial pressure is one of the main conditions contributing to the development of pulmonary arterial hypertension and occurs as a result of vascular remodeling caused by increased cell proliferation and reduced apoptosis, which leads to the narrowing of the vessel lumen and an increase in vascular resistance due to the inflammatory and fibrotic processes [241]. Rajabi et al. (2020) investigated the effects of quercetin on pulmonary arterial pressure in an experimental model of pulmonary arterial hypertension induced by monocrotaline, a plant-derived secondary metabolite that induces high acute lung toxicity once it is activated by the cytochromes P450 during hepatic metabolism [241,242]. They observed that quercetin was effective in attenuating inflammation and reducing arteriole thickness, as well as increasing miR-204 expression, a small non-coding RNA that has been associated with tumor suppression tumor and increased susceptibility to chemical treatments [243] Moreover, quercetin also decreased gene and protein expression of Poly ADP-ribose polymerase-1 (PARP1), a repair protein that is activated due to DNA damage in severe inflammation, hypoxia-inducible factor 1-alpha (HIF1α), nuclear factor of activated T-cells cytoplasmic 2 (NFATc2), and α-Smooth muscle actin (α-SMA), which are associated with apoptosis resistance and proliferation [241].

##### Acute Lung Injury Induced by Paraquat

In a model of acute lung damage induced by the highly toxic herbicide paraquat, Chen et al. (2022) developed an inhalable quercetin–alginate nanogel to improve the solubility and bioavailability of quercetin [244]. The authors observed that aerosol inhalation was effective in reversing paraquat-induced acute lung injury, alleviating oxidative stress, promoting antioxidant response, and downregulating gene and protein expression of pro-inflammatory cytokines [244].

##### Acrylamide-Induced Pulmonary and Hepatic Toxicity

The beneficial effects of quercetin have also been evaluated in acrylamide-induced pulmonary and hepatic toxicity [245]. El-Megharbel et al. (2024) synthesized a novel artemisinin/quercetin/zinc mixed-ligand complex and observed that its administration alleviates acrylamide-induced hepatic and pulmonary toxicity, improving all biochemical inflammatory markers, such as C-reactive protein (CRP), ALT, AST, and LDH, and demonstrating a potent antioxidant capacity against oxidative stress [245].

##### Carrageenan-Induced Pleural Inflammation

Studies have also investigated the role of quercetin in an experimental model of pleural inflammation induced by carrageenan, a type of natural carbohydrate extracted from seaweed [246]. The results showed that quercetin has systemic protective effects, decreasing oxidative stress (reduced lipid peroxidation) and increasing antioxidant protection (GSH and ceruloplasmin, an antioxidant enzyme responsible for most of the blood’s copper transport [247]. However, in the lungs, no reduction in redox imbalance was observed after 24 h—demonstrating beneficial, but temporary, effects [246].

##### PM_2.5_-Induced Lung Injury

PM_2.5_ particles are harmful air pollutants that can lead to acute exacerbation and worsening of respiratory diseases [248]. Ding et al. (2024) demonstrated that treatment with quercetin decreases pulmonary inflammation and fibrosis, lipid peroxidation, iron, and ferroptosis markers in mice with lung injury induced by the air pollutant PM_2.5_ [248]. Furthermore, quercetin increased nuclear Nrf2 expression and decreased Keap1 expression in lung tissue, suggesting that Nrf2 is involved in ferroptosis in PM_2.5_-induced lung injuries and that quercetin may attenuate these effects by activating the Nrf2-Keap1 pathway [248].

##### Silica-Induced Pulmonary Fibrosis

In a rat model of silicosis, a lung fibrotic disease caused by the inhalation of silica-containing dust, the intratracheal administration of chitosan-assisted encapsulation of quercetin in nanoparticles significantly enhanced the anti-fibrotic effects of quercetin [249]. The nanocarrier-assisted delivery reduced oxidative stress by decreasing ROS and MDA production, and it suppressed inflammation by inhibiting the release of IL-1β and TNF-α. Furthermore, treatment with chitosan-assisted encapsulation of quercetin in nanoparticles improved lung histology, downregulated the fibrotic marker α-smooth muscle actin, and reduced extracellular matrix deposition, thereby ameliorating pulmonary fibrosis. These findings suggest that chitosan nanoparticle delivery greatly augments quercetin’s therapeutic efficacy, providing a promising and feasible option for silicosis therapy with minimal systemic toxicity [249].

Similarly, treatment with dihydroquercetin significantly attenuated silica-induced inflammation and fibrosis in lung tissue in mice [250]. In vitro, treatment was shown to significantly decrease the expression of fibrosis markers such as α-SMA, type I collagen, and fibronectin, as well as decrease the morphological characteristics of ferroptosis, iron accumulation, and lipid peroxidation products. On the other hand, treatment with dihydroquercetin increased GSH and GPx4 levels. Taken together, the authors demonstrated that treatment suppressed ferritin phage through downregulation of the microtubule-associated protein 1 light chain 3 (LC3) protein and upregulation of ferritin pes chain 1 (FTH1) and nuclear receptor coactivator 4 (NCOA4) [250].

Fibrosis is a complex pathological condition, and although much has been conducted in advancing the understanding and treatment of it, there are still gaps in knowledge that prevent definitive treatments. Quercetin seems to be a special molecule for controlling fibrosis. Using cells of patients with idiopathic pulmonary fibrosis and bleomycin pulmonary fibrosis in aged mice (a senescence model akin to idiopathic pulmonary fibrosis), it was observed that quercetin, although not inducing apoptosis per se, enhances caveolin-1 and reduces Akt activation. This results in enhancement of susceptibility to ligand-triggered cell death of senescent fibroblasts via Fas ligand (FasL) and TNF-related apoptosis-inducing ligand (TRAIL). As a result, there is improved survival and weight gain [251]. Therefore, quercetin is an intriguing flavonoid with mechanisms of action that depend on the disease context and that are not entirely demonstrated yet.

##### Cigarette Smoke-Induced Lung Injury

Cigarette smoke is the leading cause of chronic obstructive pulmonary disease (COPD). Exposure to cigarette smoke triggers persistent airway inflammation and structural changes that lead to a progressive decline in lung function, directly and indirectly stimulating airway epithelial cells and macrophages, resulting in the production of ROS, interleukins, chemokines, and proteases. Oxidative stress is central to the disease’s pathophysiology, as it not only exacerbates chronic inflammation but also contributes to fibrosis, emphysema, and corticosteroid resistance. Therefore, targeting both inflammatory responses and oxidative stress is a promising strategy for managing COPD [252].

In conjunction, the effects of quercetin were evaluated in the treatment of cigarette smoke exposure in mice [253]. The results showed that quercetin reduced leukocyte recruitment, oxidative stress, changes in the histological pattern of lung parenchyma, and alterations in lung function [253].

Similarly, luteolin shows promise in treating COPD by mitigating inflammation and oxidative stress induced by cigarette smoke [252]. The compound reduces pro-inflammatory cytokines and boosts antioxidant defenses. Mechanistically, luteolin binds to and downregulates NOX4, a key source of ROS, which in turn inactivates the NF-κB signaling pathway. This dual action on the NOX4/NF-κB axis positions luteolin as a potential therapeutic for COPD [252]. Interestingly, Zhou et al. (2023) found an association between the expression of the nociceptive ion channel transient receptor potential vanilloid 1 (TRPV1) and luteolin-induced improved symptoms of COPD. The authors demonstrated that luteolin increased body weight, reduced lung tissue edema and lung damage indices, as well as attenuated systemic oxidative stress levels and decreased alveolar fusion in mice with cigarette smoke-induced COPD. The mechanisms associated with these findings seem to involve TRPV1/SIRT6 and cytochrome P450 family 2 subfamily A member 13 (CYP2A13)/Nrf2 signaling pathways regulation by the flavonoid [254].

Epicatechin increased cell viability and repressed ROS production by upregulating tripartite motif-containing protein 25 (TRIM25), which promotes the ubiquitin-mediated degradation of Keap1 [255]. This action enhances the nuclear localization of the antioxidant transcription factor Nrf2. Concurrently, epicatechin significantly inhibited the activation of the NLRP3 inflammasome and reduced pyroptosis, as evidenced by decreased caspase-1 and pro-inflammatory cytokines like IL-1β and IL-18. Nrf2 knockdown partially reversed Epicatechin’s protective effects, confirming its central role. These findings suggest Epicatechin is a promising therapeutic strategy for COPD, acting via the Nrf2-NLRP3 pathway [255].

##### Lung Ischemia-Reperfusion Injury

Zardak et al. (2024) investigated the effects of quercetin in preventing pulmonary ischemia-reperfusion injury and observed that the treatment had protective effects, reducing inflammation markers, oxidative stress, pulmonary edema, apoptosis, and preventing histopathological alterations [256]. These effects were modulated by the Nrf2/Keap1 pathway and by the inhibition of the NF-κB pathway, demonstrating a multifaceted approach of quercetin [256].

##### Lung Injury or Damage Caused by Hypoxia or Hyperoxia

Hypoxia is characterized by a reduction in the availability of O_2_ in tissues or cells, thus compromising tissue homeostasis [257]. Hypoxia increases the generation of free radicals and, consequently, oxidative stress [258]. Tripathi et al. (2020) correlated the prophylactic potential of quercetin in improving the lungs’ ability to eliminate fluids through the resensitization of beta-2 adrenergic receptor (β2-AR) signaling under hypoxic conditions [258]. As a result, they observed that mice treated with quercetin and exposed to hypobaric hypoxia had a significant increase in the expression of β2-AR, G protein-coupled receptor (GPR)-1, GPR-10, GCSα, and cAMP, as well as a decrease in the expression of G protein-coupled receptor kinase 2 (GRK)-2, β-arrestin, ROS, and NF-κB—mechanisms that contributed to better clearance of alveolar fluid [258].

Hyperoxia can be defined as excessive O_2_ exposure (above physiological levels) to tissues and organs, which increases ROS and causes toxic effects [259]. Exposure of lungs, especially immature ones, to hyperoxia also contributes to lung injury and airway hyperreactivity [260]. Kryeziu et al. (2023) demonstrated that newborn rats exposed to hyperoxia and treated with quercetin showed reduced contraction in response to methacholine, restoring relaxation. Furthermore, there was a decrease in pro-inflammatory cytokine expression, indicating that quercetin may exert a protective effect under these conditions [260].

Exposure to diesel exhaust particles is responsible for causing pulmonary abnormalities through increased inflammation and hypoxia [261]. Jeong et al. (2023) demonstrated that dietary quercetin significantly attenuated the presence of eosinophils in bronchoalveolar lavage fluid, reduced the expression of pulmonary cytokines, and hypoxia response mRNA [261].

##### Radiation-Induced Lung Damage

Rajabinasab et al. (2025) investigated the protective effects of apigenin against radiation-induced lung damage [262]. The group observed that the administration of apigenin reversed the adverse effects induced by radiation by decreasing alveolar wall thickness and macrophage and lymphocyte infiltration, as well as reducing the levels of inflammatory [NF-κB, glycogen synthase kinase-3 beta (GSK-3β), and transforming growth factor beta 1 (TGF-β1)] and epigenetic factors [DNA methyltransferase 3a (DNMT3A) and histone deacetylase 2 (HDAC2)] [262].

**Table 11:** Summary of the literature on selected flavonoids against chemically or physically induced lung damage from different agents, from 2020 to 2025.

Chemically or physically induced lung toxicity				
AUTHORS	TREATMENTS	SUBJECTS	MODEL INDUCTION	MAIN FINDINGS
Cypermethrin-induced lung toxicity				
Ileriturk et al. (2022) [239]	• Quercetin [25 or 50 mg/kg] • Oral administration 30 min after cypermethrin administration • For 28 days	• Male Sprague–Dawley rats [220–250 g] • 9–10 weeks old	• Cypermethrin [25 mg/kg] • Oral administration • For 28 days	Quercetin effects compared to stimulus group: • ↑ SOD, CAT, GPx, and GSH levels • ↓ MDA level • ↓ LC3A and LC3B mRNA (autophagy markers) • ↓ IL-1β, NF-κB, TNF-α, and iNOS mRNA expression • ↑ Nrf2 and HO-1 mRNA expression • ↓ Caspase-3, cytochrome c, and Bax protein expression • ↑ Bcl-2 protein expression
Owumi et al. (2021) [240]	• Luteolin [50–100 mg/kg] • Oral administration • For 14 days	• Male albino Wistar rats [160 ± 5 g]	• Doxorubicin [2 mg/kg] • Intraperitoneal administration • Every other day for 6 days	Luteolin effects compared to stimulus group: • ↓ XO, MDA, and ROS • ↑ Weight • ↑ CAT, SOD, GPx, GST, GSH, and TSH • ↓ NO, MPO, TNF-α, IL-1β, and caspase-1 • ↑ IL-10 • ↓ Thickening of the alveolar wall
Rajabi et al. (2020) [241]	• Quercetin [30 mg/kg] • Intraperitoneal administration • For 3 weeks	• Male Wistar rats [220–280 g] • 8–10 weeks old	• Monocrotaline [60 mg/kg] • Subcutaneous administration • Single dose	Quercetin effects compared to stimulus group: • ↓ Inflammation and arteriole thickness • ↑ miR-204 • ↓ PARP1, HIF1α, NFATc2, and α-SMA
Chen et al. (2022) [244]	• Quercetin–alginate nanogel [50–150 mg/kg] • Administered by inhalation	• Sprague-Dawley rats [200 ± 20 g] • 6–8 weeks old	• Paraquat [20 mg/kg] • Intraperitoneal administration	Quercetin–alginate nanogel effects compared to stimulus group: • ↓ Lung injury • ↑ CAT • ↓ MDA • ↓ TNF-α, IL-6, IL-1β protein and mRNA expression
El-Megharbel et al. (2024) [245]	• Artemisinin/Quercetin/Zinc mixed ligand complex [30 mg/kg] • Oral administration • For 30 days	• Male rats [150–180 g]	• Acrylamide [500 mg/kg] • Oral administration • For 30 days	Artemisinin/Quercetin/Zinc mixed ligand complex effects compared to stimulus group: • ↓ CRP, LDH, IL-6, and TNF-α • ↑ CAT, SOD, and GPx • ↓ MDA • ↓ Hepatotoxicity • Restored normal hepatic structures • ↓ Caspase-3 • ↓ Pulmonary fibrosis • ↑ Normal pulmonary sacculus and alveolar epithelial cells
Bidian et al. (2020) [246]	• Quercetin [50 mg/kg] • Intraperitoneal administration • For 3 weeks	• Male Wistar rats [200–220 g]	• Carrageenan [0.2 mL/1%] • Intrapleural injection	Quercetin effects compared to stimulus group: • ↓ MDA • ↑ GSH and ceruloplasmin • ↓ Redox imbalance
Ding et al. (2024) [248]	• Quercetin [50 and 100 mg/kg] • Oral administration • For 60 days	• Male C57BL/6 mice [18–22 g] • 6–8 weeks old	•PM_2.5_ particulates [5 mg/kg] • Intratracheal instillation • Once every two days, 20 times in total	Quercetin effects compared to stimulus group: • ↓ Lung inflammation, lung fibrosis, and lipid peroxidation • ↓ Iron contents and ferroptosis markers • ↑ Nrf2 • ↓ Keap1
Silica-induced pulmonary fibrosis				
Yao et al. (2023) [249]	• Chitosan-assisted encapsulation of Quercetin in nanoparticles [2 mg/animal] • Intratracheal administration	• Sprague–Dawley rats [180–220g]	• Silica particles [50 mg/mL] • Intratracheal administration	Quercetin effects compared to stimulus group: • ↓ ROS and MDA levels • ↓ IL-1β and TNF-α
Yuan et al. (2022) [250]	•Dihydroquercetin [10 and 50 mg/kg] • Oral administration • For 14 days	• Male C57BL/6 mice • 8 weeks old	• Silica suspension [20 mg in 50 μL saline] • Instilled in the mice	Dihydroquercetin effects compared to stimulus group: • ↓ Weight loss • ↑ Lung weight/body weight ratio • ↓ Inflammatory reaction, deposition of collagen fibers, and pulmonary fibrosis • ↓ IL-1β, TNF-α, and TGF-β • ↓ Expression of α-SMA, collagen I, and fibronectin
Cigarette smoke-induced lung injury				
Araújo et al. (2020) [253]	• Quercetin [10 mg/kg] • Oral administration	• Male C57BL/6 mice	• Smoking chamber: 12 cigarettes per day • For 6 days	Quercetin effects compared to stimulus group: • ↓ Volume densities of the alveolar air space • ↓ Carbonylated proteins and TBARS
Li et al. (2023) [252]	• Luteolin [20 and 40 mg/kg] • Oral administration • For 75 days	• Male BALB/c mice (20–25 g) • 6–8 weeks old	• Cigarette smoke [100 mg TPM/m^3^ and 250 mg TPM/m^3^) • 6 h/day, 5 days/week • For 75 days	Luteolin effects compared to stimulus group: • ↑ SOD and CAT • ↓ IL-1β, IL-6, TNF-α, and IL-8 • ↓ p- NF-κB • ↑ p-IκBα and HO-1
Zhou et al. (2023) [254]	• Luteolin [50 and 100 mg/kg] • Oral administration • Starting from the 15th week, one hour before cigarette smoke exposure	• Male SPF C57BL/6J	• Intratracheal instillation of LPS [10 mg/kg] combined with smoking chamber [20 cigarettes per day]	Luteolin effects compared to stimulus group: • ↓ MDA and LDH • ↑ SOD, CAT, and GSH • ↓ TRPV1 and CYP2A13 • ↑ SIRT6, Nrf2, PGC1α, SOD1 and 2
Tian et al. (2021) [255]	• Epicatechin [5, 15, 30 and 45 mg/kg] • Oral administration	• Male Wistar rats (250–300 g) • 12–13 weeks old	• Smoking chamber: 15 cigarettes per day divided into two times daily for 12 consecutive weeks	Epicatechin effects compared to stimulus group • ↓ ROS • ↑ HO1 and NQO1 mRNA • ↓ Keap1 • ↑ HO1, NQO1, SOD1, SOD2, and SOD3 • ↑ Nuclear Nrf2 • ↓ TRIM25 • ↓ NLRP3 inflammasome (NLRP3, GSDMD-N, caspase-1, IL-18, and IL-1β)
Lung ischemia-reperfusion injury				
Zardak et al. (2024) [256]	• Quercetin [30 mg/kg] • Intraperitoneal administration • For 7 days	•Male Wistar rats [190–210 g]	• The left pulmonary artery, vein, and bronchus were obstructed for 60 min, followed by a reperfusion period of 120 min	Quercetin effects compared to stimulus group • ↓ Lung wet/dry weight ratio • ↓ IL-1β and TNF-α • ↓ MDA and TOS • ↑ SOD, TAC, and GPx • ↓ Bax • ↑ Bcl2 • ↓ NF-κB and Keap1 • ↑ Nrf2 and HO-1 • ↓ Alveolar hemorrhage, neutrophil infiltration, and lug injury score
Lung injury or damage caused by hypoxia or hyperoxia				
Tripathi et al. (2020) [258]	• Quercetin [50 mg/kg]	• Male Sprague Dawley rats [180–200 g]	• Hypobaric hypoxia chamber	Quercetin effects compared to stimulus group: • ↑ β2-AR, GPR-1, GPR-10, GCSα and cAMP • ↓ GRK-2, β-arrestina, ROS and NF-κB
Kryeziu et al. (2023) [260]	• Quercetin [10 mg/kg] • Intraperitoneal administration • For 7 days	• Wistar rats	• Hyperoxia [FiO_2_ > 95%] • For 7 days	Quercetin effects compared to stimulus group: • ↓ Contraction • ↑ Relaxation of the tracheal smooth muscle • ↓ TNF-α and IL-1β
Diesel exhaust particles-induced lung damage				
Jeong et al. (2023) [261]	• Dietary quercetin [20 or 100 mg/kg] • For 14 days	• Male C57BL/6 mice	• Diesel exhaust particles instillation	Quercetin effects compared to stimulus group: • ↓ Eosinophils in the bronchoalveolar lavage fluid analysis, • ↓ Pulmonary cytokine • ↓ mRNA expression of hypoxia markers • ↓ Hyperactivity triggered by diesel exhaust particles
Radiation-induced lung damage				
Rajabinasab et al. (2025) [262]	• Apigenin [10 and 20 mg/kg] • Intraperitoneal administration • For 7 days	• Wistar rats	• Rats were irradiated once with 6 Gy whole-body X-ray after 7 days of treatment with Apigenin	Apigenin effects compared to stimulus group: • ↓ Alveolar wall thickness and macrophages and lymphocytes infiltration • ↓ NF-κB, GSK-3β, TGF-β1, DNMT3α, and HDAC2 expression • ↑ IκB-α expression

Abbreviations: α-SMA (alpha-smooth muscle actin), β2-AR (beta 2 adrenergic receptor), Bax (Bcl-2 Associated X protein), Bcl-2 (B-cell lymphoma 2), cAMP (cyclic adenosine monophosphate), CAT (catalase), GPx (glutathione peroxidase), CRP (c-reactive protein), LDH (lactate dehydrogenase), CY2A13 (cytochrome P450 2A13), DNMT3α (DNA methyltransferase 3 alpha), GCSα (Guanylate cyclase soluble subunit alpha-1), GPR-1 (G protein-coupled receptor 1), GPR-10 (G protein-coupled receptor 10), GRK-2 (G protein-coupled receptor kinase 2), GSDMD-n (N-terminal domain of Gasdermin D), GSH (reduced glutathione), GSK-3β (glycogen synthase kinase 3 beta), GST (glutathione S-transferase), HDAC2 (histone deacetylase 2), HIF-1α (hypoxia-inducible factor 1-alpha), HO-1 (heme oxygenase-1), IL-8 (interleukin-8), IL-10 (interleukin-10), IL-18 (interleukin-18), IL-1β (interleukin-1 beta), IL-6 (interleukin-6), iNOS (inducible nitric oxide synthase), IκB-α (nuclear factor of kappa light polypeptide gene enhancer in B-cells inhibitor alpha), Keap1 (Kelch-like ECH-associated protein 1), LC3A (Microtubule-associated protein 1 light chain 3 alpha), LC3B (Microtubule-associated protein 1 light chain 3 beta), MDA (malondialdehyde), MPO (myeloperoxidase), NF-κB p65 (nuclear factor of kappa light polypeptide gene enhancer in B-cells subunit p65), NFATC2 (nuclear factor of activated T cells 2), NLRP3 (NOD-, LRR- and pyrin domain-containing protein 3), NO (nitric oxide), NQO1 (NAD(P)H quinone oxidoreductase 1), Nrf2 (Nuclear factor erythroid 2-related factor 2), p-IκB-α (phosphorylated nuclear factor of kappa light polypeptide gene enhancer in B-cells inhibitor alpha), p-NF-κB (phosphorylated nuclear factor of kappa light polypeptide gene enhancer in B-cells), PARP1 (poly ADP-ribose polymerase-1), ROS (reactive oxygen species), SIRT6 (Sirtuin 6), SOD (superoxide dismutase), SOD1 (superoxide dismutase 1), SOD2 (superoxide dismutase 2), SOD3 (superoxide dismutase 3), TAC (total antioxidant activity), TBARS (thiobarbituric acid reactive substances), TGF-β (transforming growth factor beta), TNF-α (tumor necrosis factor-alpha), TOS (total oxidant status), TRIM25 (tripartite motif containing 25), TRPV1 (transient receptor potential vanilloid 1), TSH (thyroid-stimulating hormone), XO (xanthine oxidase), ↑ (increase), ↓ (decrease).

Overall, pre-clinical data, and even results obtained from human cells, support that flavonoids are conceivable active molecules to treat pulmonary diseases ranging from acute respiratory distress syndrome, insecticide and herbicide lung toxicity, antineoplastic drug lung dysfunction, pulmonary arterial hypertension, pleural inflammation, silica fibrosis, senescent fibrosis, idiopathic pulmonary fibrosis, cigarette smoke lung inflammation, lung ischemia-reperfusion, hypoxia, hyperoxia and radiation lung damage. This seems a promising organ-related disease field for flavonoids. It is also likely that flavonoids can be used together with other treatments, not solely as isolated treatments.

#### 5.3.2. Pathogen-Related- or Pathogen-Induced Lung Damage

Acute pneumonia, an infection of the pulmonary parenchyma, is a leading cause of morbidity and mortality, characterized by a complex inflammatory response critical for pathogen clearance. The disease is initiated when pathogens, such as bacteria, viruses, or fungi, overcome host defenses to reach the alveoli. This occurs most commonly via aspiration of oropharyngeal flora or inhalation of contaminated droplets. Initial host defenses, including mechanical barriers and resident flora, are the first line of protection. Upon breaching these defenses, pathogens are met by resident alveolar macrophages and surfactant proteins A and D, which act as innate immune sentinels. A persistent infectious challenge triggers a robust inflammatory cascade, mediated by the release of pro-inflammatory cytokines (e.g., IL-1, TNF, IL-8). This leads to neutrophil recruitment, an alveolar-capillary leak, and subsequent fluid accumulation within the alveoli, resulting in hypoxemia. At the tissue level, this process unfolds in distinct phases: an initial edema, followed by red and gray hepatization as erythrocytes, neutrophils, and fibrin dominate, before culminating in a resolution phase in which macrophages clear debris. An excessive pro-inflammatory response can escalate to systemic complications like sepsis and organ failure [263,264,265]. Table 12 summerizes the main findings on selected flavonoids against pathogen-related- or pathogen-induced lung damage.

##### LPS-Induced Pulmonary Injury

Yu et al. (2024) elucidate the protective mechanisms of quercetin-3-glucuronide (Q3G) against LPS-induced pulmonary injury, identifying a novel link between its anti-inflammatory effects and the dual activation of the Nrf2 and autophagy pathways [266]. In vitro, Q3G treatment attenuated LPS-induced cell injury and oxidative stress by suppressing ROS generation. This was achieved by activating Nrf2-antioxidant signaling, a process that involved repressing Nrf2 ubiquitination and enhancing the association between Keap1 and p62. Furthermore, Q3G promoted autophagy, as evidenced by the formation of acidic vesicular organelles and the upregulation of autophagy-related factors. The protective effects of Q3G were significantly reduced when either Nrf2 signaling was inhibited via siRNA or when autolysosome formation was blocked with a lysosomal inhibitor. In vivo results confirmed that Q3G significantly alleviated LPS-induced lung dysfunction and edema. These findings collectively demonstrate that Q3G exerts its lung-protective effects by a concerted activation of both the Nrf2 and autophagy pathways, providing new mechanistic insights into its therapeutic potential for acute lung injury [266].

Deng et al. (2023) demonstrated that quercetin significantly improved LPS-induced acute lung injury in a murine model, reducing histopathological changes, the release of pro-inflammatory cytokines, and ROS generation, as well as inhibiting ferroptosis [267]. Mechanistically, the treatment activated the SIRT1/Nrf2/GPx4 signaling pathway [267]. Similarly, Chen et al. (2022) demonstrated that quercetin administration significantly reduces lung injury and the production of pro-inflammatory cytokines, as well as inhibiting NLRP3 inflammasome activation, suppressing pyruvate kinase isozyme M2 (PKM2) nuclear accumulation and increasing SIRT1 levels [268].

Furthermore, studies demonstrate that dihydroquercetin attenuates histopathological changes, pulmonary edema, and apoptosis in mice with LPS-induced acute lung injury. The treatment affected M2 macrophage polarization and indicators related to cell injury, as well as increased interferon regulatory factor (IRF) 4 and miR-132-3p expression, inhibited the Notch pathway, and decreased F-box/WD repeat/containing protein 7 (FBXW7) levels, indicating that macrophage polarization occurred via IRF4/miR-132-3p/FBXW7 [269].

Studies also demonstrate that intranasal administration of quercetin reduces nasal inflammation and exerts a protective effect on the pulmonary inflammatory response induced by LPS [208]. These findings were mediated by a decrease in exudate and the degree of inflammation in the lamina propria of the nasal and sinus areas, along with a reduction in the secretion of TNF-α and IL-6. In the lungs, there was also a reduction in these pro-inflammatory cytokines [208]. Regarding cytokines, the results of Shaker et al. (2024) also demonstrated similar effects [270]. In this study, quercetin was effective in reducing IL-6 and TNF-α levels in the lungs of animals induced with LPS, protecting against tissue damage [270].

Studies with nanoparticles demonstrate that flavonoids, such as rutin and quercetin, may have superior effects to conventional treatments. Zhang et al. (2024) developed D-mannitol–cerium–quercetin/rutin coordination polymer nanoparticles (MCQ/R NPs) and demonstrated that their administration significantly reduces lung tissue inflammation in animals with LPS-induced acute lung injury [271].

Another approach to overcome the low availability and low solubility of quercetin is the development of liposomes loaded with this flavonoid. Zhai et al. (2023) investigated the effects of quercetin-loaded liposomes in a sepsis model induced by LPS challenge [272]. As a result, it was observed that liposomal encapsulation promoted the inhibitory effects of quercetin on pulmonary inflammation, decreased mortality in septic mice, and reduced NF-κB-dependent cytokine production and inflammasome activation [272].

Collectively, these studies indicate that quercetin and its derivatives exert consistent protective effects against LPS-induced pulmonary injury through multiple, interconnected mechanisms. Beyond reducing histopathological damage, pulmonary edema, and pro-inflammatory cytokine production, these compounds modulate key pathways involved in oxidative stress, ferroptosis, inflammasome activation, autophagy, and macrophage polarization.

The effects of other flavonoids, such as luteolin, have also been investigated in LPS-induced acute lung injury models. Chen et al. (2023) demonstrated that luteolin improves the pathological structure of the lungs and reduces the wet/dry weight ratio, as well as reducing bronchoalveolar protein and levels of pro-inflammatory cytokines [236]. Mechanistically, these effects were partially mediated by increased transepithelial sodium transport via the JAK/STAT pathway [236]. Li et al. (2024) demonstrated that inhalation of an aerosol containing luteolin-7-O-glucuronide (L7Gn) significantly reduced LPS-induced lung injury by inhibiting inflammatory infiltrate, the NLRP3 inflammasome and its components, and pro-inflammatory cytokines [273].

Zhou et al. (2024) demonstrated that the stimulator of interferon genes (STING) signaling pathway participates in the pathogenesis of acute lung injury and evaluated the role of apigenin in LPS-induced injury [274]. As a result, it was observed that apigenin reduced type I IFN synthesis in response to STING pathway agonists. Furthermore, the treatment reduced pathological lung inflammation and pulmonary edema, as well as attenuated inflammatory mediators and innate immune responses [274].

Sepsis-induced acute lung injury is a major contributor to high mortality rates in sepsis patients. Its primary pathogenesis involves a devastating interplay between acute inflammation and damage to the endothelial barrier [275]. Gao et al. (2024) investigated the therapeutic potential of kaempferol in an LPS-induced sepsis model in both mice and human umbilical vein endothelial cells (HUVECs) [275]. As a result, kaempferol significantly mitigated lung tissue damage and exhibited potent anti-inflammatory effects, evidenced by a reduction in pro-inflammatory cytokines such as IL-6 and TNF-α. Mechanistically, kaempferol protected the endothelial barrier by suppressing the hyperphosphorylation of myosin light chain 2 (MLC2) and downregulating the Sphingosine kinase 1 (SphK1)/S1P/S1PR1 signaling pathway. This modulation subsequently restores the expression of key tight junction proteins, including zonula occludens-1 (ZO-1), vascular endothelial cadherin (VE-cadherin), and Occludin. These results highlight kaempferol as a promising therapeutic candidate for sepsis-induced acute lung injury, acting by strengthening the pulmonary endothelial barrier through the modulation of the SphK1/S1P/S1PR1/MLC2 pathway [275].

Epicatechin shows promise for treating acute lung injury induced by LPS. In a mouse model, it reduced pulmonary edema and histopathological damage [276]. Epicatechin’s protective effects stem from its ability to suppress key pro-inflammatory cytokines while simultaneously inhibiting the MAPK and NF-κB signaling pathways, which are central to the inflammatory response. These findings suggest that epicatechin could be a therapeutic agent for pulmonary inflammation [276]. Taken together, these findings indicate that flavonoids other than quercetin also exert significant protective effects in LPS-induced acute lung injury. Although they act through distinct molecular targets, these compounds consistently reduce pulmonary inflammation, cytokine production, and tissue damage. Overall, this body of evidence reinforces the therapeutic potential of flavonoids as multi-target agents for the prevention and treatment of acute lung injury.

##### Pulmonary Diseases Induced by Other Pathogens

*Pseudomonas aeruginosa* is an opportunistic pathogen causing severe infections, particularly in immunocompromised patients. Studies investigated the therapeutic potential of quercetin in mitigating acute lung inflammation induced by *P. aeruginosa* [277]. Utilizing a combined approach of network pharmacology, molecular docking, and experimental validation, it was demonstrated that quercetin effectively protects against *P. aeruginosa*-induced lung injury. In vivo, quercetin treatment significantly reduced neutrophil infiltration, the production of pro-inflammatory cytokines (IL-1β, IL-6, and TNF), and ultimately improved survival rates in a murine model. Mechanistically, this protective effect was linked to the suppression of the PI3K/Akt/NF-κB signaling pathway. Furthermore, quercetin treatment decreased the phosphorylation levels of key regulatory proteins—PI3K, Akt, IκBα, and NF-κB p65—both in the lung tissues of treated mice and in *P. aeruginosa*-infected macrophages. These findings suggest that quercetin’s anti-inflammatory properties are mediated by its ability to inhibit the PI3K/Akt/NF-κB pathway, positioning it as a promising therapeutic candidate for treating acute lung inflammation caused by *P. aeruginosa* [277].

Similarly, Wang et al. (2025) investigated the protective mechanism of kaempferol against *P. aeruginosa*-induced acute pneumonia [278]. Network pharmacology and molecular docking analyses identified GSK-3β, JNK, and NF-κB as key targets of kaempferol. In both a mouse model and in vitro macrophage cultures, kaempferol treatment effectively attenuated lung inflammation by reducing the overproduction of pro-inflammatory cytokines like TNF, IL-1β, IL-6, and MIP-2. Mechanistically, kaempferol was found to inhibit the GSK-3β kinase by increasing its inactivating phosphorylation. This inhibition subsequently suppressed the downstream activation of the GSK-3β/JNK/c-Jun and NF-κB signaling pathways. These findings suggest that kaempferol mitigates *P. aeruginosa* pneumonia not by direct bacteriostatic effects but by modulating the host’s inflammatory response. This makes kaempferol a promising therapeutic candidate, particularly as an adjunct to antibiotics, to improve treatment outcomes and potentially reduce the risk of antibiotic resistance [278]. Other flavonoids, such as luteolin, are also promising treatments for acute *P. aeruginosa* pneumonia. It improves survival and reduces inflammation in mice by inhibiting the epidermal growth factor receptor (EGFR)/PI3K/Akt/NF-κB and EGFR/ERK/AP-1 signaling pathways [279]. This leads to a decrease in inflammatory cytokines, neutrophil infiltration, and bacterial load, while also shifting macrophages from a pro-inflammatory (M1) to an anti-inflammatory (M2) state [279].

Studies with other genera of bacteria, such as *Mycoplasma*, observed that apigenin protects against necroptosis induced by *Mycoplasma hyopneumoniae* in alveolar macrophages [280]. The bacterial infection triggers a pro-necroptotic cascade initiated by autocrine TNF-α. Apigenin effectively inhibits this process by suppressing TNF-α production and activating PPARγ, which subsequently upregulates the transcription of ubiquitin-like with prolyl hydroxylase domain (PHD) and RING finger domains 1 (Uhrf1). Uhrf1 then increases DNA methylation of the TNF-α promoter, thereby silencing its expression. This pathway ultimately promotes alveolar macrophage survival and reduces lung injury in infected mice, highlighting apigenin as a promising therapeutic agent for *Mycoplasma* pneumonia [280].

Finally, An et al. (2024) evaluated the effects and mechanisms of quercetin treatment in a respiratory syncytial virus (RSV)-induced infection model. As a result, quercetin was responsible for negatively regulating glycolysis and TCA metabolism in RSV-infected alveolar macrophages. Furthermore, it increased itaconic acid production through inhibition of immune response gene 1 (IRG1) and succinate dehydrogenase (SDH) activity—an enzyme responsible for the negative regulation of HIF-1α/NLRP3 signaling and, consequently, for the polarization of M1 to M2 macrophages [281].

Taken together, these studies broaden the therapeutic relevance of flavonoids beyond sterile LPS-induced injury, encompassing infectious models of lung disease, including *P. aeruginosa*, *Mycoplasma*, and RSV. Despite differences in pathogens and molecular targets, flavonoids attenuate lung inflammation, cytokine release, and tissue damage by modulating key pathways involved in inflammatory signaling. Overall, these findings highlight flavonoids as promising multi-target agents for the treatment of infectious lung inflammation.

##### Acute Lung Injury Secondary to Cecal Ligation and Puncture (CLP) Sepsis

Sang et al. (2022) investigated the protective mechanisms of quercetin against acute lung injury secondary to CLP sepsis, focusing on its effects on endoplasmic reticulum stress and mitochondrial dysfunction [282]. Using a CLP mouse model and LPS-stimulated lung epithelial cells, it was demonstrated that quercetin effectively ameliorates pulmonary damage and oxidative stress. Mechanistically, quercetin treatment was found to inhibit endoplasmic reticulum stress by downregulating markers such as C/EBP homologous protein (CHOP) and GRP78, while simultaneously improving mitochondrial function, as evidenced by increased mitochondrial membrane potential and reduced ROS production. Transcriptome analysis revealed that these effects are mediated by the upregulation of the SIRT1/AMPK pathway. Furthermore, genetic knockdown of SIRT1 abrogated quercetin’s antioxidant effects in vitro, confirming its critical role. These findings suggest that quercetin protects against acute lung injury by suppressing ER stress and mitochondrial dysfunction through the induction of the SIRT1/AMPK pathways [282].

Studies have also investigated the effects of luteolin on acute lung injury induced by sepsis through CLP. Xie et al. (2021) demonstrated that luteolin attenuated lung damage and systemic inflammation [283]. The therapeutic effect of the compound is linked to its ability to inhibit NF-κB signaling and reduce pro-inflammatory cytokines such as TNF-α, IL-1β, and IL-6. Crucially, luteolin also modulates the immune response, promoting the differentiation of regulatory T cells (Tregs) and increasing IL-10 production. This leads to a shift in macrophage polarization from an inflammatory (M1) to a reparative (M2) phenotype, thus facilitating lung tissue repair [283].

Celebi et al. (2021) investigated the protective effects of luteolin in a rat model of CLP sepsis-induced acute lung injury [284]. Administered post-sepsis, luteolin significantly ameliorated lung tissue damage, a finding supported by both histopathological examination and biochemical markers. Luteolin treatment reduced oxidative stress, as evidenced by a decrease in MDA levels and an unexpected reduction in antioxidant markers like GSH and SOD activity. The compound also demonstrated potent anti-inflammatory properties, suppressing the mRNA expression of key cytokines such as TNF-α and IL-10. These findings suggest that luteolin, through its antioxidant and anti-inflammatory actions, is a promising therapeutic candidate for mitigating sepsis-induced acute lung injury [284].

Similar effects of luteolin were observed by Zhang et al. (2023) in acute lung injury secondary to the CLP sepsis model [285]. Treatment with luteolin reduced systemic inflammation and lung damage in septic mice. Mechanistically, these effects were associated with an anti-pyroptosis effect via Akt1, nitric oxide synthase 2 (NOS2), and cathepsin G (CTSG) [285]. Furthermore, Zhang et al. (2021) demonstrated that luteolin significantly inhibits inflammation and lung injury, as well as levels of caspase-11, caspase-1, gasdermin D (GSDMD), IL-1α, and IL-1β [286]. Taken together, the treatment increased the frequency of Treg cells and IL-10 levels [286].

Neonatal sepsis is a life-threatening inflammatory condition. Zhang et al. (2021) evaluated the effect of luteolin in a neonatal sepsis-induced lung injury model (cecal suspension injection; CSI) [287]. The results showed that luteolin administration decreased cold-inducible RNA-binding protein (CIRP) mRNA and protein, improved lung architecture, and reduced pulmonary edema and apoptosis. Furthermore, luteolin reduced the expression of HIF-1α and NLRP3 [287]. These findings demonstrate that luteolin has potent therapeutic actions for sepsis in both adult and neonatal mice.

Acute lung injury is driven by a severe inflammatory response, with the Toll-like receptor 7 (TLR7) pathway playing a critical role. Geng et al. (2025) demonstrate that in septic mice, macrophages polarize toward a pro-inflammatory M1 phenotype, a process dependent on TLR7 activation [288]. Genetic deficiency of TLR7 was found to promote a shift towards the anti-inflammatory M2 phenotype, thereby attenuating acute lung injury. Building on this, the natural flavone apigenin was shown to significantly reduce sepsis-induced lung inflammation and injury. Mechanistically, apigenin achieves this by directly blocking the binding of TLR7 to its agonist, miR-146a, thereby inhibiting macrophage-driven inflammation in a TLR7-dependent manner. These findings position TLR7 as a key therapeutic target and highlight apigenin as a promising lead compound for the treatment of sepsis and other inflammatory diseases [288].

Cicek et al. (2021) investigated the protective effects of apigenin against sepsis (CLP)-induced acute lung injury in a rat model [289]. Sepsis significantly increased pro-inflammatory cytokines (TNF-α, IL-1β, IL-6) while decreasing the anti-inflammatory cytokine IL-10, leading to a severe inflammatory response. Concurrently, oxidative stress was heightened, evidenced by elevated lipid peroxidase (LPO) and reduced levels of antioxidants such as SOD, CAT, and GSH [289]. Furthermore, sepsis induced significant apoptosis, as shown by increased immunoreactivity of Bax and Caspase-3. Apigenin treatment effectively reversed these pathological changes. It attenuated the inflammatory cytokine storm, restored antioxidant balance, and significantly reduced apoptosis. These findings suggest that apigenin holds therapeutic potential for mitigating sepsis-induced lung injury by inhibiting both inflammatory and oxidative stress pathways [289].

Thus, in addition to pulmonary diseases that are not initiated by pathogens, flavonoids are also active against pulmonary diseases initiated by infections. This is an important aspect of flavonoids’ activity because immunosuppressive drugs can make the host susceptible to infections, and this is not the case for flavonoids.

**Table 12:** Summary of the literature on selected flavonoids against pathogen-related- or pathogen-induced lung damage, from 2020 to 2025.

Pathogen-related or pathogen-induced lung damage				
AUTHORS	TREATMENTS	SUBJECTS	MODEL INDUCTION	MAIN FINDINGS
LPS-induced pulmonary injury				
Chen et al. (2023) [236]	• Luteolin [70 μmol/kg] • Intraperitoneal administration	• Male BALB/c mice [20–25g]	•LPS [5mg/kg] • Intraperitoneal administration	Luteolin effects compared to stimulus group • ↑ Expression of ENaC • ↓inflammatory cell infiltration
Yu et al. (2024) [266]	• Quercetin-3-glucuronide [0.15 μmol] • Oral administration • For 30 days	• Male C57BL/6 mice • 6 weeks old	• LPS [7 μg] • Intranasal administration	Quercetin effects compared to stimulus group: • ↓ SOD, GSH, and Nrf2 • ↓ Beclin-1, p62 and LC3-II • ↓ HO-1 and NQO1
Deng et al. (2023) [267]	• Quercetin [20, 40 or 60 mg/kg] • Oral administration • Single dose	• Male C57BL/6 mice [22 g] • 6–8 weeks old	• LPS [5 mg/kg] • Intratracheal administration	Quercetin effects compared to stimulus group: • ↓ Edema, alveolar wall thickness, and the cellular infiltration • ↓ Protein leakage • ↓ Ferroptosis • ↓ TNF-α, IL-6, and IL-1β • ↑ GSH and GPx • ↓ MPO, MDA, iron, ROS, and 4-HNE • ↑ Expression of SIRT1 and Nrf2
Chen et al. (2022) [268]	• Quercetin [50 mg/kg] • Intraperitoneal administration • For 3 days	• Male and female C57BL/6 mice [22 g] • 8–12 weeks old	• LPS [20 mg/kg] • Intraperitoneal administration	Quercetin effects compared to stimulus group: • ↓ Lung inflammation and alveolar wall destruction • ↓ Lactate synthesis • ↓ Pulmonary permeability • ↑ Survival • ↓ TNF-α, IL-6, and IL-1β • ↓ NLRP3 inflammasome activation • ↑ mRNA levels of SIRT1 • ↑ PKM2
Li et al. (2023) [269]	• Dihydroquercetin [25–50 mg/kg] • Intraperitoneal administration	• Balb/c mice [20 -22 g] • 6–8 weeks old	• LPS [10 mg/kg] • Intranasal instillation	Dihydroquercetin effects compared to stimulus group: • ↓ Tissue damage, apoptosis, and lung wet-dry ratio • ↓ M1 marker CD86, iNOS and TNF-α • ↑ M2 marker CD206, CD163, and Arg-1 • ↓ IL-1β, IL-6, and TNF-α • ↓ FBXW7 and proteins Notch1 and HES1 • ↑ IRF4 and miR-132-3p
Zhang et al. (2024) [271]	• MCQ/R NPs [100 mg/kg] • Intraperitoneal administration • For 5 days	• Balb/c mice [20–25 g] • 6–8 weeks old	• LPS [5 mg/kg] • Intraperitoneal administration	Quercetin effects compared to stimulus group: • ↓ Lung wet-to-dry weight ratio • ↓ Inflammatory cells • ↓ Alveolar size, thickness of the alveolar septa, inflammatory infiltration, and pulmonary interstitial edema • ↓ IL-1β, IL-6, and TNF-α • ↓ TLR4 and NLRP3 mRNA expression
Zhai et al. (2023) [272]	• Free quercetin and Liposomal quercetin • Intraperitoneal administration • Single dose	• C57/B6 mice	• LPS [3 mg/kg] • Intraperitoneal administration	Quercetin effects compared to stimulus group: • ↓ Leukocyte infiltration • ↓ IL-1β and TNF-α mRNA levels • ↓ IL-1β and IL-18
Tiboc-Schnell et al. (2020) [208]	• Quercetin [80 mg/kg] • Intranasal administration • For 7 days	• Female Wistar rats [100 ± 10 g] • 20 days	• LPS [5 or 10 μg] • Intranasal administration • For 7 days	Quercetin effects compared to stimulus group: • ↓ TNF-α and IL-6 • ↓ Exudate and inflammation
Shaker et al. (2024) [270]	• Quercetin [100 mg/kg] • Intraperitoneal administration	• BALB/c mice [20–25 g] • 7 weeks old	• LPS [5 mg/kg] • Intraperitoneal administration	Quercetin effects compared to stimulus group: • ↓ TNF-α, IL-1β and IL-6 • ↓ Tissue damage
Gao et al. (2024) [275]	• Kaempferol [1, 3, 9 mg/kg] • Intravenous administration	• Male BALB/c mice	• LPS [10 mg/kg] • Intravenous administration	Kaempferol effects compared to stimulus group • ↓ Edema, cell infiltration, and vascular permeability • ↓ IL-6 and TNF-α • ↑ ZO-1, VE-cadherin, and occludin • ↓ SphK1/S1P/S1PR1/MLC2 • Preservation of alveolar structure
Li et al. (2024) [273]	• Luteolin (L7Gn) [4 mg/mL] • Inhalation	• Male Wistar rats [220 g]	• LPS [4 mg/mL] • Intranasal administration	Luteolin effects compared to stimulus group • ↓ NLRP3 Inflammasome • ↓ IL-1β and IL-18
Zhou et al. (2024) [274]	• Apigenin [50 mg/kg] • Intraperitoneal injection	• Male C57BL/6 mice (20–24 g) • 8 week old	• LPS [5 mg/kg] • Intraperitoneal administration • Treatment after 24h LPS administration	Quercetin effects compared to stimulus group: • ↓ IL-6, IL-1β, and TNF -α, • ↓ IFNB1, CXCL10, and ISG15 • ↓ mRNA levels of IFNB1 IL-6, IL-1β, TNF-α, CXCL10, ISG15
Li et al. (2022) [276]	• Epicatechin [25, 50, and 100 mg/kg] • Oral administration	• BALB/c mice	• LPS [5 mg/kg] • Intraperitoneal administration	Epicatechin effects compared to stimulus group: • ↓ TNF-α, IL-6, and IL-1β • ↓ iNOS and COX-2 mRNA • ↓ phosphorylation of ERK, JNK, p38, p65, and IκB-α
*P. aeruginosa*-induced acute lung inflammation				
Jia et al. (2024) [277]	• Quercetin [12.5 and 50 mg/kg] • Intraperitoneal administration	• C57BL/6 mice (20–24 g) • 8 weeks old	• Intranasal *P. aeruginosa* infection • Intraperitoneal administration	Quercetin effects compared to stimulus group: • ↓ MPO activity • ↓ IL-1β, IL-6 and TNF-α • ↓ PI3K, Akt, IκB-α and NF-κB p65
Wang et al. (2025) [278]	• Kaempferol [2.5, 10 and 30 mg/kg] • Intraperitoneal administration after intranasal *P. aeruginosa* infection	• Male C57BL/6N mice (20–24 g) • 8 weeks old	• Intranasal *P. aeruginosa* infection • Intraperitoneal injection • 2 doses after 8h	Kaempferol effects compared to stimulus group • ↓ TNF, IL-1β, IL-6, and MIP-2 • ↓ activation of GSK3β, JNK, c-Jun, and NF-κB p65
Gu and Pang (2025) [279]	• Luteolin [20 and 60 mg/kg] • Intraperitoneal administration	• Male C57BL/6 mice • 8 weeks old	• Intranasal *P. aeruginosa* infection	Luteolin effects compared to stimulus group • ↓ MPO activities • ↓ IL-1β, IL-6, TNF-α and MIP-2 • ↑ IL-10
*Mycoplasma*-induced acute lung inflammation				
Mei et al. (2023) [280]	•Apigenin [5, 10 and 20] mg/kg/day] • Intraperitoneal administration • For 7 days	• Female Balb/c mice • 6–8 weeks old	• *M. hyopneumoniae* infection intranasal administration	Apigenin effects compared to stimulus group • ↓ Expression of Uhrf1 and TNF-α, • ↓ Necroptosis in primary alveolar macrophages,
Pulmonary infection induced by respiratory syncytial virus				
An et al. (2024) [281]	• Quercetin [30, 60 or 120 mg/kg] • Oral administration	• BALB/c mice	• Respiratory syncytial virus • Intranasal administration	Quercetin effects compared to stimulus group • ↑ Itaconic acid • ↓ SDH and SDH/HIF-α/NLRP3 • ↓ Inflammation • Polarization of alveolar macrophages from M1 to M2
Cecal ligation and puncture-induced acute lung injury				
Sang et al. (2022) [282]	• Quercetin [50 mg/kg] • Oral administration	• C57BL/6 mice (19–27 g) • 8–10 weeks old	• Cecal ligation and puncture	Quercetin effects compared to stimulus group • ↓ ROS and MDA • ↓ TNF-α, IL-6 and IL-1β
Celebi et al. (2020) [284]	• Luteolin [2 and 4 mg/kg] • Intraperitoneal administration	• Female albino Wistar rats (300 g)	• Cecal ligation and puncture	Luteolin effects compared to stimulus group: • ↑ SOD, GSH • ↓ TNF-α and IL-10 mRNA
Zhang et al. (2023) [285]	• Luteolin [20 mg/kg] • Intraperitoneal administration	• Male C57BL/6 mice • 8 weeks old	• Cecal ligation and puncture	Luteolin effects compared to stimulus group: • ↓ Levels of MPO and IL-1β and TNF-α • ↓ Expression of NOS2 and GSDMD • ↑ Expression of Akt
Xie et al. (2021) [283]	• Luteolin [0.2 mg/kg] • Intraperitoneal administration	• Male C57BL/6 mice • 8–12 weeks old	• Cecal ligation and puncture	Luteolin effects compared to stimulus group: • ↓ Lung injury • ↓ IL-1β, IL-6, TNF-α, and IL-17A • ↑ IL-10 • ↓ MPO^+^ neutrophils • ↓ Lung weight/dry weight ratio • ↓ Nuclear translocation of NF-κB p65 and NF- κB p65 phosphorylation activation (p-NF- κB p65) • ↑ CD45^+^, CD25^+^, FOXP3^+^ • ↓ M1 cells • ↑ M2 cells
Zhang et al. (2021) [287]	• Luteolin [10 mg/kg] • Intraperitoneal administration	• C57BL/6 mouse pups [5–7 days]	• Cecal slurry injection • Intraperitoneal administration	Luteolin effects compared to stimulus group: • ↓ CIRP mRNA and protein • ↓ Edema and apoptosis • ↓ HIF-1α and NLRP3
Cicek et al. (2021) [289]	• Apigenin [20, 40 and 60 mg/kg] • Intraperitoneal administration	• Female Wistar (200–220 g) • 12–16 weeks old	• Cecal ligation and puncture	Apigenin effects compared to stimulus group: • ↑ SOD, CAT, GSH • ↑ IL-10 • ↓ Bax and caspase-3 • ↓ TNF-α, IL-1β, and IL-6
Geng et al. (2025) [288]	• Apigenin [30 mg/kg] • Intraperitoneal administration • For 3 days	• Male C57BL/6 J and • Tlr7tm1Flv/J • 8–16 weeks old	• Cecal ligation and puncture	Apigenin effects compared to stimulus group: • ↓ Albumin, IL-6, and IL-1β • ↑ miR-146a
Zhang et al. (2021) [286]	• Luteolin [20 mg/kg] • Intraperitoneal administration	• Male C57BL/6 mice • 6–8 weeks old	• Cecal ligation and puncture	Luteolin effects compared to stimulus group: • ↓ IL-1β, IL-6, TNF-α, IL-17 • ↑ IL-10 • ↓ Protein levels of cleaved-caspase-11, caspase-1, cleaved-caspase-1, cleaved-GSDMD, IL-1α and IL-1β

Abbreviations: Akt (protein kinase B), Arg-1 (arginase-1), Bax (Bcl-2 Associated X protein), CAT (catalase), CD163 (cluster of differentiation 163), CD25 (cluster of differentiation 25), CD45 (cluster of differentiation 45, also known as leukocyte common antigen—LCA), CD86 (cluster of differentiation 86), CIRP1 (CAT2 interacting RING protein 1), COX-2 (cyclooxygenase-2), CXCL10 (C-X-C motif chemokine ligand 10), ENaC (epicatechinthelial sodium channel), ERK (extracellular signal-regulated kinase), FBXW7 (F-box with 7 tandem WD40), FOXOP3 (forkhead box protein P3), GPx (glutathione peroxidase), GSDMD (gasdermin D), GSH (reduced glutathione), GSK-3β (glycogen synthase kinase 3 beta), HES1 (hairy and enhancer of split-1), HIF-1α (hypoxia-inducible factor 1-alpha), HO-1 (heme oxygenase-1), IFNB1 (interferon beta 1), IκB-α (nuclear factor of kappa light polypeptide gene enhancer in B-cells inhibitor alpha), IL-10 (interleukin-10), IL-17 (interleukin-17), IL-18 (interleukin-18), IL-1β / IL-1a (interleukin-1 beta/ interleukin-1 alpha), IL-6 (interleukin-6), iNOS (inducible nitric oxide synthase), CD206 (cluster of differentiation 206), IRF4 (interferon regulatory factor 4), ISG15 (interferon-stimulated gene 15), JNK (c-Jun N-terminal kinases), LC3-II (lipidated form of Microtubule-associated protein 1A/1B-light chain 3), M1 (classically activated macrophages), M2 (alternatively activated macrophages), MDA (malondialdehyde), MIP-2 (macrophage inflammatory protein-2), MLC1 (megalencephalic leukoencephalopathy with subcortical cysts), MPO (myeloperoxidase), NF-κB (p65 nuclear factor of kappa light polypeptide gene enhancer in B-cells subunit p65), NLRP3 (NOD-, LRR- and pyrin domain-containing protein 3), NOS2 (nitric oxide synthase 2), Notch1 (Neurogenic locus notch homolog protein 1), NQO1 (NAD(P)H quinone oxidoreductase 1), Nrf2 (nuclear factor erythroid 2-related factor 2), p38 (p38 mitogen-activated protein kinase), PI3K (phosphoinositide 3-kinase), PKM2 (pyruvate kinase muscle isozyme M2), ROS (reactive oxygen species), 4-HNE 4-Hydroxynonenal, S1P (sphingosine-1-phosphate), S1PR1 (sphingosine-1-phosphate receptor 1), SIRT1 (sirtuin 1), SOD (superoxide dismutase), SphK1 (sphingosine kinase 1), TLR4 (toll-like receptor 4), TNF-α (tumor necrosis factor-alpha), Uhrf1 (ubiquitin-like containing PHD and RING finger domains 1), ↑ (increase), ↓ (decrease).

#### 5.3.3. Allergic Airway Inflammation and Asthmatic Model of Pulmonary Damage

Asthma is a prevalent chronic inflammatory lung condition characterized by airway inflammation, remodeling, and bronchial hyperresponsiveness. The intricate pathophysiology of this disease involves a complex interplay of cellular and molecular mechanisms, including oxidative stress, cellular senescence, ferroptosis and adaptive immune response. Exogenous allergens, such as pollen and dust mite, activate T helper 2 (Th2) lymphocytes, which secrete key interleukins like IL-4, IL-5 and IL-13. This leads to B cell proliferation and the production of immunoglobulin E (IgE) antibodies. The binding of IgE to high-affinity IgE receptor (FcεRI) on mast cells triggers degranulation, releasing a potent mix of pro-inflammatory mediators (e.g., histamine, leukotrienes) that drive bronchoconstriction, mucus hypersecretion, and airway inflammation [290]. Eosinophils also express FcεRI and respond to Th2 cytokines, contributing to allergic pulmonary inflammation [291].

A defining pathological feature of asthma is airway remodeling, a process of relentless injury and repair that alters lung tissue structure and function. This remodeling includes subepithelial fibrosis, goblet cell hyperplasia, and airway smooth muscle (ASM) cell proliferation, which collectively contribute to airway obstruction and a decline in pulmonary function [292,293]. A critical cellular mechanism underlying these changes is the epithelial–mesenchymal transition (EMT). During EMT, airway epithelial cells lose their epithelial markers (E-cadherin, ZO-1) and acquire a mesenchymal phenotype, characterized by increased expression of N-cadherin, vimentin, and α-SMA [294,295]. These transformed cells, particularly myofibroblasts, are major producers of extracellular matrix proteins (e.g., collagen, fibronectin), which are responsible for the fibrotic thickening of the airway wall [296].

Central to EMT regulation is the Wnt/β-catenin signaling pathway. Under normal conditions, newly synthesized cytoplasmic β-catenin is targeted for degradation by a destruction complex that includes GSK-3. However, the phosphorylation and inactivation of GSK-3β disrupt this complex, leading to the cytoplasmic accumulation and subsequent nuclear translocation of β-catenin. In the nucleus, β-catenin acts as a co-activator for transcription factors like T-cell factor (TCF)/lymphoid enhancer-binding factor (LEF), driving the expression of genes that promote EMT and fibrosis. Understanding these intricate pathways provides a strong rationale for developing novel therapies that target EMT and airway remodeling to improve long-term asthma management [297].

Table 13 summarizes studies with the selected flavonoid using allergic airway inflammation experimental models induced by ovalbumin. Quant et al. (2024) demonstrated that luteolin suppressed airway inflammation and attenuated airway remodeling associated with epithelial–mesenchymal transition—effects resulting from β-catenin blockade [297]. Taken together, the treatment reduced bronchial wall thickness when compared to the untreated group [297].

Fang et al. (2023) investigated the anti-asthmatic effect of quercetin in ovalbumin-sensitized mice [298]. As a result, treatment with quercetin reduced periostin expression in bronchoalveolar fluid and the inflammatory response, fibrosis, and airway hyperreactivity. Furthermore, quercetin reduced the expression of the TGF-β1/Smad pathway in lung tissue, improving asthma symptoms and providing a protective effect [298].

Similarly, Rajizadeh et al. (2023) demonstrated that quercetin reduced the expression of the *Gata3*, *Tnf*, *Tgfb1*, *Il1b*, and *Acta2* genes and increased the expression of the *Tbx21* gene in animals with ovalbumin-induced asthma [299]. Furthermore, the treatment decreased oxidative stress and IL-6 and TNF-α levels and increased IL-10 levels in lung tissue [299]. These findings reinforce an anti-asthmatic role for quercetin.

Studies have also investigated the actions of kaempferol on allergic airway inflammation induced by ovalbumin. Molitorisova et al. (2020) demonstrated that kaempferol ameliorated chronic airway inflammation by significantly decreasing levels of key Th2 cytokines, including IL-5 and IL-13, as well as granulocyte-macrophage colony-stimulating factor (GM-CSF) and eosinophil counts in the bronchoalveolar lavage fluid [300]. The treatment also reduced the pro-fibrotic protein TGF-β1 in lung tissue and normalized cough reflex sensitivity without affecting ciliary beat frequency. These findings demonstrate that kaempferol effectively modulates allergic airway inflammation and associated asthma features like airway hyperresponsiveness and cough reflex abnormalities [300].

Similarly, treatment with kaempferol 3-O-gentiobioside also mitigated mucus hypersecretion by downregulating mucin 5AC (MUC5AC) expression [301]. Mechanistically, these protective effects were linked to the inhibition of the NOTCH signaling pathway, with kaempferol 3-O-gentiobioside specifically targeting the NOTCH1 receptor. Furthermore, kaempferol 3-O-gentiobioside suppressed the activation of the NLRP3 inflammasome and the TLR4/NF-κB pathway. These findings suggest that kaempferol 3-O-gentiobioside is a promising therapeutic candidate for allergic asthma, acting by suppressing inflammation and mucus overproduction through the NOTCH signaling axis [301].

Other flavonoids, such as luteolin, show promise in treating allergic asthma by modulating autophagy. In an ovalbumin-induced asthmatic mouse model, luteolin significantly attenuated airway hyperresponsiveness, mucus hypersecretion, and inflammatory cell infiltration [302]. The treatment also reduced levels of key pro-inflammatory cytokines like IL-4, IL-5, and IL-13, and decreased ovalbumin-specific IgE in the serum. Crucially, the study found that luteolin’s therapeutic effects are linked to its ability to inhibit the excessive autophagy observed in the asthmatic lung. Mechanistically, luteolin achieved this by activating the PI3K/Akt/mTOR pathway and inhibiting the formation of the Beclin-1-PI3KC3 complex. These findings provide biological evidence for luteolin’s potential as a clinical agent for allergic asthma by targeting autophagy [302].

Regarding apigenin, studies have demonstrated its therapeutic effects in ovalbumin-induced chronic asthma in obese patients [303]. It was observed that treatment with apigenin suppressed inflammation and acted as a regulator of immune homeostasis. Furthermore, it reduced airway hyperresponsiveness, inflammatory cell infiltration, airway epithelial cell apoptosis, collagen deposition, and pulmonary oxidative stress. Mechanistically, these effects were mediated by the ROS-ASK1-MAPS pathway [303].

Epicatechin shows promise as a treatment for allergic asthma [304]. In a mouse model, epicatechin reduced lung inflammation and key cytokines. This protective effect stems from its ability to inhibit NF-κB signaling while activating the Nrf2-HO-1 antioxidant pathway. The study also found that epicatechin beneficially altered the gut microbiota, suggesting a dual mechanism for its anti-asthmatic properties [304].

Thus, flavonoids are also active against allergic pulmonary inflammation. The mechanisms of flavonoids for pulmonary inflammation in general depend on the disease context, likely because of their multi-target mechanism. This aspect of flavonoid mechanisms of action may be crucial for their adaptive nature, making these molecules beneficial in varied disease contexts.

In general, studies suggest that flavonoids have broad protective potential in pulmonary disorders. Although the conditions mentioned above differ substantially in their causes and pathological progression, they share common mechanisms, including oxidative stress and uncontrolled inflammation. In this context, flavonoids appear to act not through a single pathway, but by modulating multiple interconnected biological processes. This multi-target profile may explain why compounds such as quercetin, luteolin, apigenin, kaempferol, and epicatechin exhibit beneficial effects in different models of lung injury. However, current evidence is still largely experimental, with considerable variation in disease models, doses, routes of administration, formulations, and outcomes evaluated. Thus, despite the promising results, the therapeutic relevance of flavonoids for lung diseases remains dependent on additional translational data. It is also interesting to note that there is a negative correlation between asthma and flavonoid intake [305]. This suggests that assessing flavonoid intake is an essential parameter in clinical settings [306,307,308]. While there is evidence of activity and disease outcome improvement by flavonoid intake as discussed above, it is possible that in patients who already ingest enough flavonoids, additional nutraceutical use of flavonoids may present limited activity, while nutraceutical flavonoid treatment would be more beneficial to patients with low ingestion of flavonoids [34,309,310].

## 6. Clinical Trials on Flavonoid or Flavonoid-Containing Dietary Intake Against Inflammatory Conditions

### 6.1. Clinical Trials on Quercetin

Table 14 summarizes clinical trials on quercetin intake in the treatment of inflammatory conditions related to selected systems (liver, nervous system, lung), between 2020 and 2025. Regarding the liver, a randomized, double-blind, placebo-controlled pilot study was conducted to investigate the effects of quercetin on hematological parameters in patients with non-alcoholic fatty liver disease (NAFLD). For this study, 90 patients with NAFLD received supplementation with quercetin capsules (500 mg) or placebo twice daily for 12 weeks, and blood samples were collected at the end of the treatment period for laboratory analysis. The results showed a significant increase in erythrocytes, mean corpuscular hemoglobin concentration, and mean platelet volume, while mean corpuscular volume, red blood cell distribution width, platelet distribution width, and ferritin decreased. These findings demonstrated that quercetin has a hematological modulating effect and promotes significant improvements in hematological parameters in patients with NAFLD [311].

In addition, a randomized, double-blind, placebo-controlled clinical trial, involving 41 patients with NAFLD treated with quercetin (500 mg/day) or placebo for 12 weeks, with a 4-week washout period before switching treatments, showed that quercetin significantly reduced intrahepatic lipid content (from 11.5% ± 6.4% to 9.6% ± 5.8%; *p* = 0.013; adjusted *p* = 0.028) compared to placebo, and promoted a modest reduction in body weight and BMI (*p* < 0.05). There was a positive correlation between weight loss and reduction in liver fat, with the effect being more pronounced in women. No relevant adverse events were reported. Thus, the study concludes that supplementation with quercetin for 12 weeks can reduce liver fat in patients with NAFLD, possibly due to the slight weight loss induced by the treatment [312].

Furthermore, a double-blind, randomized, controlled study is underway to investigate the effects of combined treatment with quercetin and dasatinib in patients with NAFLD. However, no results have yet been published [313].

Regarding the nervous system, current clinical studies investigate the effects of quercetin on Alzheimer’s disease. Gonzales et al. (2023) conducted an open-label phase I clinical trial to investigate the safety, feasibility, and efficacy of the combined senolytic therapy of dasatinib and quercetin in symptomatic patients in the early stages of Alzheimer’s disease. For the study, five participants completed the 12 weeks of investigations, and as a result, it was observed that the treatment was well tolerated and presented a favorable safety profile. However, only dasatinib was detected in the cerebrospinal fluid and demonstrated efficacy in increasing IL-6 levels and decreasing senescence-related cytokines and chemokines [314].

More recently, an open-label pilot study also investigated the safety, feasibility, and efficacy of dasatinib and quercetin combination therapy. For the study, 12 patients aged 65 or older received the combination treatment for two consecutive days, every two weeks, over a 12-week period. To date, the results demonstrate modest treatment effects, but with some positive signs for cognition and inflammation, making it necessary to conduct larger, controlled trials [315].

Regarding the respiratory system, a randomized, double-blind, placebo-controlled clinical trial conducted by Han et al. (2020) evaluated the safety of quercetin supplementation in patients with COPD with forced expiratory volume in 1 second between 35% and 80%. Participants received placebo or escalating doses of quercetin (500, 1000, or 2000 mg/day) for one week. The results showed that quercetin was well tolerated, with no serious adverse events related to treatment, as determined by blood tests and pulmonary function tests. Only mild effects, such as gastroesophageal reflux, were reported in both the placebo and quercetin groups. These findings indicate that quercetin, even at high doses, is safe for individuals with chronic obstructive pulmonary disease [316].

Similarly, Patel et al. (2025) conducted a phase II pilot clinical study to examine the effects of quercetin on inflammation in patients with COPD [317]. The results showed that the treatment was safe and well-tolerated, and plasma quercetin levels increased significantly after treatment. Furthermore, bronchoalveolar lavage fluid levels of IL-8, IL-1β, and 8-isoprostane, as well as serum SP-D levels, were significantly different in these patients, and reported symptoms showed a tendency to decrease. Further prospective studies are needed to confirm these effects [317].

Another clinical study investigated the effects of quercetin on epithelial remodeling from bronchopulmonary cells of COPD patients. In this study, cells were treated with quercetin for three days, and bronchial brushing samples from COPD patients treated with quercetin (2000 mg/day) or placebo for months were used to confirm the effects. In the cells, quercetin significantly increased transepithelial resistance, the number of ciliated cells, and the expression of genes associated with tissue and epithelial development and differentiation. Furthermore, it decreased the number of goblet cells and IL-8 levels. In patients, there was also an increase in genes associated with tissue and epithelial development and differentiation and a reduction in TGF-β and IL-8 levels [318]. Another clinical trial aimed at evaluating whether quercetin reduces oxidative stress and inflammation in patients with COPD is currently in the recruitment phase [319].

Finally, a randomized, open-label, controlled clinical trial was conducted with 100 outpatients with mild to moderate symptoms of COVID-19. Participants were divided into two groups: one received standard treatment combined with oral quercetin supplementation (Quercetin Phytosome^®^, 500 mg), and the other received standard treatment alone. After one week, the group receiving quercetin showed faster recovery, with a higher number of patients testing negative for SARS-CoV-2 (34 vs. 12) and earlier resolution of symptoms (26 vs. 12). A significant reduction in LDH levels was also observed, indicating less inflammation. The supplementation was well tolerated, with no reports of adverse effects. These results suggest that quercetin may have a promising therapeutic role in the early stages of COVID 19 [320].

Taken together, the available clinical evidence suggests that quercetin is a safe and promising adjuvant compound for inflammatory disorders affecting the liver, nervous system, and respiratory tract. Thus, these findings corroborate the translational potential of quercetin in human inflammatory diseases, while reinforcing the need for larger, well-controlled clinical trials to confirm its efficacy and define its therapeutic indications more clearly. Controlling the ingestion of other nutraceuticals and assessing individual intake of flavonoids within their regular diet are parameters essential to identifying the activity of quercetin and discriminating whether, in patients with reasonable flavonoid intake, supplementation would still be beneficial or not [308].

**Table 14:** Clinical trials on quercetin intake in the treatment of inflammatory conditions related to selected systems (liver, nervous system, lung), from 2020 to 2025.

Quercetin intake in the treatment of inflammatory conditions				
AUTHORS	TREATMENTS	STUDY PHASE AND PARTICIPANTS	DISEASE	MAIN FINDINGS
Pasdar et al. (2020) [311]	• Quercetin [500 mg] • Oral administration • For 12 weeks	• Randomized, double-blind, placebo-controlled pilot study • Iranian Center for Clinical Trials Identifier: IRCT2016060628299N1 • 90 participants with nonalcoholic fatty liver disease	• Nonalcoholic fatty liver disease	Quercetin effects: • ↑ Red blood cell, mean corpuscular hemoglobin concentration, and mean platelet volume • ↓ Mean corpuscular volume, red blood cell distribution width-coefficient of variation, platelet distribution width, and ferritin
Li et al. (2024) [312]	• Quercetin [500 mg] • Oral administration • For 12 weeks • 4 weeks washout period	• Randomized, double-blind, placebo-controlled crossover clinical trial • Trial is registered at www.chictr.org.cn as ChiCTR2100047904 • 41 participants with nonalcoholic fatty liver disease	• Nonalcoholic fatty liver disease	Quercetin effects: • ↓ Intrahepatic lipid • ↓ Body weight and body mass
Lipsitz (2025)[315]	• Quercetin [1250 mg] and Dasatinib [100 mg] • Oral administration • 2 consecutive days, every 2 weeks over a period of 12 weeks	• Single arm, open label, pre-post pilot study • Trial is registered at ClinicalTrials.gov as NCT05422885 • 12 adults aged 65 or older with slow gait speed and mild cognitive impairment	• Alzheimer’s disease	Quercetin and dasatinib effects: • ↑ Cognition and brain function • Slight improvement in overall cognitive function and neurovascular coupling • ↓ Load of senescent cells and IL-6
Han et al. (2020) [316]	• Quercetin [500, 1000 or 2000 mg] • Oral administration • For 1 week	• Phase I clinical trial • Trial is registered at ClinicalTrials. gov as NCT01708278 • COPD patients with mild to moderate disease between the age group of 40 to 65 years	• COPD	Quercetin effects: • No drug-related severe adverse events • One of the patients reported mild adverse events
Patel et al. (2025) [317]	• Quercetin [2000 mg] • For 6 months	• Pilot phase II clinical trial • 14 patients with ≥ pack-year smoking history and CRP > 3 mg/L	• COPD	Quercetin effects: • ↑ Plasma quercetin levels • Alteration in the levels of IL-8, IL-1β, 8-isoprostane, and SP-D • ↓ Disease symptoms • No drug-related adverse events
McCluskey et al. (2024) [318]	• Quercetin [1 µM or 2000 mg] • For 3 days or 6 months	• Phase I and II clinical trial • Airway basal cells from patients with COPD	• COPD	Quercetin effects: • ↑ Transepithelial resistance and ciliated cells • ↓ Fewer goblet cells • ↑ HOXB2 and ELF3 • ↓TGF-β and IL-8
Di Pierro et al. (2023) [320]	• Quercetin [500 mg] • Oral administration • For 1 week	• Open-label randomized controlled clinical trial • Trial is registered at ClinicalTrials.gov as NCT04861298 • Outpatients with early-stage mild to moderate symptoms of COVID-19	• SARS-CoV-2	Quercetin effects: • ↑ Speedy recovery • ↓ LDH • No drug-related adverse events

Abbreviations: IL-6 (interleukin 6); IL-1β (interleukin-1 beta); IL-8 (interleukin-8); LDH (lactate dehydrogenase); SP-D (surfactant protein-D); HOXB2 (homeobox B2); ELF3 (early flowering 3); TGF-β (transforming growth factor beta); COPD (chronic obstructive pulmonary disease), ↑ (increase), ↓ (decrease).

### 6.2. Clinical Trials on Kaempferol

No clinical trial has been conducted investigating kaempferol in diseases affecting the liver, nervous system or the respiratory system within 2020–2025. There were three clinical trials in healthy volunteers, which suggest a safe profile for the treatment with kaempferol.

A double-blind, randomized, placebo-controlled crossover trial in 13 male athletes found that a single 10 mg oral dose of kaempferol did not improve 400 meters sprint times but significantly reduced respiratory and heart rates during repeated sprints and lowered markers of muscle damage (myoglobin, aspartate transaminase). This suggests kaempferol may help reduce cardiopulmonary load and muscle damage during strenuous exercise, making it a potentially useful supplement for athletes under high physical stress [321].

A randomized, double-blind, placebo-controlled trial evaluated the safety of high-dose kaempferol aglycone (50 mg daily for 4 weeks) in 24 healthy adults. No significant changes were observed in anthropometric, blood, or urine parameters, and no adverse events were reported. The study concluded that this dosage is safe for short-term use in healthy individuals [322].

A double-blind, placebo-controlled, randomized, crossover trial with 33 city workers assessed the effects of 10 mg kaempferol daily for 2 weeks. Kaempferol intake led to increased daily step count, distance covered, recreational activities, and improved sleep quality. Heart rate decreased during physical activity and sleep, suggesting potential benefits for long-term quality of life through behavioral changes [322].

### 6.3. Clinical Trials on Luteolin

No clinical trial has been conducted using luteolin as treatment for diseases affecting the liver, nervous system or the respiratory system within 2020–2025. Nonetheless, four clinical trials have been carried out, suggesting a safe profile for the treatment with luteolin [323,324,325].

In the randomized clinical trial by Terzo et al. (2023), a combination of chlorogenic acid and luteolin was given for six months to individuals with pre-obesity. Supplementation resulted in significant improvements in cardiometabolic parameters, such as a reduction in waist circumference, lower fasting glucose levels, decreased insulin resistance, an improved lipid profile, and reduced inflammatory markers, all without notable adverse effects. These findings highlight luteolin as a promising component of a nutraceutical approach to managing cardiometabolic risk factors [324].

Finally, Sánchez-Macarro et al. (2020) examined a combination of citrus flavones (luteolin), flavanones, and olive oil polyphenols in 100 healthy volunteers. After eight weeks, there was a significant reduction in LDL, along with an increase in plasma antioxidant capacity, improved endothelial function, and a decrease in inflammatory markers, all with no significant adverse effects. The study indicates that this combination could be a promising nutritional approach to preventing cardiovascular disease [325].

In addition, Cárdenas-Rodríguez et al. (2025) have recently reviewed the role of luteolin in post-COVID syndrome and emphasized how luteolin can cross the blood–brain barrier, inhibit microglia activation, and reduce inflammatory cytokines, which helps alleviate persistent neurological symptoms such as mental fog, fatigue, and anxiety [326].

### 6.4. Clinical Trials on Apigenin

Much like kaempferol and luteolin, no clinical trial has been conducted using apigenin as a treatment for inflammatory diseases affecting the liver, nervous system or the respiratory system within 2020–2025. There were two clinical trials that suggest a safe profile for the treatment with apigenin-7-O-glucoside and chamomile extract [327,328].

Borges et al. (2022) examined how apigenin is absorbed, distributed, metabolized, and excreted in healthy men. The study found that the free form of apigenin has very low bioavailability, while its glycosylated form (apigenin-7-O-glucoside) is better absorbed, with peak plasma concentrations occurring between 2 and 8 h, depending on the metabolite. These data are crucial for developing formulations that enhance its absorption and utilization [327].

### 6.5. Clinical Trials on Epicatechin

Clinical evidence for epicatechin supplementation is limited between 2020 and 2025. The available studies in humans focus mainly on the nervous system, including a clinical trial in Friedreich’s ataxia summarized in Table 15 [329]. In contrast, clinical trials specific to liver and lung diseases are still scarce or nonexistent, especially those evaluating epicatechin as an isolated compound.

Regarding liver diseases, one randomized, double-blind clinical trial investigated the effects of consuming green tea with high-density catechins (such as epicatechin) in patients with NAFLD. For the study, 17 patients consumed green tea with high-density (1080 mg/700 mL) catechins, low-density catechins (200 mg/700 mL), or placebo for 12 weeks. As a result, it was observed that body fat, serum ALT levels, and urinary excretion of 8-isoprostane decreased significantly in the group that consumed green tea with high-density catechins, suggesting that consuming 700 mL of green tea containing high-density catechins presents positive effects in patients with NAFLD [330].

Regarding the nervous system, a single-center, open-label, baseline-controlled phase II study was conducted to investigate the safety and efficacy of epicatechin in pediatric patients with Friedreich’s ataxia. For the study, 10 participants with a confirmed diagnosis of Friedreich’s ataxia received epicatechin (75–150 mg/day) for 24 weeks. The results showed that the treatment was well tolerated and there was an improvement in cardiac structure and function in one of the subgroups; however, no improvement was observed in the primary neurological outcomes (Friedreich’s ataxia assessment scale and 8-meter timed walk test) [329].

In summary, there were some clinical trials developed to assess the efficacy of the selected flavonoids or nutraceuticals containing the selected flavonoids in diseases affecting the liver, nervous system and lungs. In general, positive effects of flavonoid treatment were observed, which support their application as supplements for the adjuvant treatment of human diseases. As for any novel treatment, well-controlled clinical trials for each specific disease are obligatory.

Although all studies presented to date provide important evidence supporting the biological activity and safety of flavonoids, their therapeutic positioning remains a challenge, as these compounds do not easily fit into conventional pharmacological classifications. Flavonoids have demonstrated modulatory mechanisms in inflammatory diseases, fibrotic diseases, and infectious diseases, while most applicable drug classes generally operate within more restricted therapeutic contexts. For instance, in general, antibiotics are not applied as asthma treatment when there is no infection, though there can be exceptions to antibiotic use in inflammatory diseases such as rheumatoid arthritis, likely because of a pathobiont role in the disease [331,332]. Anti-fibrotic drugs are not used as treatments for infection [333], and immunosuppressants can increase the incidence of infections [334]. Some non-steroidal anti-inflammatory drugs can slow the progression of fibrosis [335], but they are not applied to promote infection resolution [336].

Despite this broad therapeutic potential, low absorption and bioavailability of flavonoids remain as major challenges for clinical translation. However, these limitations should be interpreted in conjunction with the generally favorable safety profile of flavonoids. For instance, quercetin clinical trials have reached a 2 g/day in an escalating dose without side effects [316]. This means that it is likely that, as there is low absorption, activity might be achieved with high doses and still without side effects. This also means that in pre-clinical studies, relatively high doses of flavonoids cannot be considered a paradigm limiting the development of these compounds into clinical application because there is low toxicity. Thus, even if the dose may seem high, if there is no toxicity, there should be no constraint because the dose would be high solely by direct comparison with molecules of other classes. Relatively high doses might be a consequence of low absorption and low bioavailability. Furthermore, limited absorption and bioavailability can be bypassed by pharmaceutical development of controlled release systems as approached elsewhere [18,308,337] or even structural modifications to increase solubility [338]. These approaches are expected to increase the actual doses of flavonoids, and in these cases, toxicity may be an unexpected concern. On the other hand, animal models applying intraperitoneal treatment (e.g., bypassing absorption limitations) with flavonoids have not encountered toxicity in therapeutic doses [18].

## 7. Modern Nutrient Delivery Systems for Flavonoid-Based Nutraceuticals: Nanoencapsulation, Phytosomes, Liposomes, and Beyond

Despite the robust preclinical and increasing number of clinical evidence for the anti-inflammatory and antioxidant activities of flavonoids, their therapeutic application as nutraceuticals continues to face a fundamental pharmacokinetic bottleneck. As discussed throughout this review, compounds such as quercetin, kaempferol, luteolin, apigenin, and epicatechin share two critical physicochemical liabilities: limited aqueous solubility and rapid phase II metabolism upon oral administration, which together yield bioavailabilities that are frequently below 10% for the aglycone form [339].

As presented in the initial topics, quercetin and kaempferol are rapidly metabolized after absorption and circulate as methylated, glucuronidated, and sulfated metabolites. Thus, their protective potential may depend not only on their original chemical structure but also on the biological activity, tissue distribution, and possible deconjugation of these metabolites at inflammatory sites [45]. In the case of kaempferol, the low oral bioavailability is associated with first-pass metabolism, especially by intestinal and hepatic glucuronidation [340].

Apigenin has low bioavailability in part due to low solubility and conjugation by UDP-glucuronosyltransferase and sulfotransferase enzymes, while luteolin also has low water solubility and low bioavailability, which limits its systemic potential [341,342]. Therefore, although both present promising protective mechanisms, their clinical application may depend on formulation strategies, such as nanoformulations or other delivery technologies.

In contrast, epicatechin appears to have a more favorable bioavailability profile. Pharmacokinetic studies in humans indicate that epicatechin is widely absorbed, although it circulates as phase II metabolites and not as a free compound [343,344]. This suggests that epicatechin may have greater translational potential compared to other flavonoids, although its biological effects should be attributed to the combined action of its conjugated metabolites and microbiota-derived catabolites.

Addressing these limitations is a prerequisite for the rational translation of flavonoid research into evidence-based nutraceutical and pharmaceutical formulations. The past decade has produced a rich landscape of advanced delivery platforms that are directly relevant to the flavonoids reviewed here, and their consideration is essential for evaluating both existing experimental results and future research directions.

### 7.1. Polymeric Nanoencapsulation

Polymeric nanoparticles—typically prepared from biodegradable materials such as chitosan, poly(lactic-co-glycolic acid) (PLGA), or zein—represent some of the most versatile and clinically translatable platforms for flavonoid delivery. By encapsulating hydrophobic flavonoids within a nanoparticle matrix, this strategy simultaneously increases aqueous dispersibility, protects the active compound from premature gastrointestinal degradation, and enables tunable release kinetics. Particle surface chemistry can be tailored for passive enhanced permeability and retention effects in inflamed tissues or, increasingly, for active receptor-mediated targeting.

Within the context of the present review, chitosan-based nanoencapsulation of quercetin has provided compelling evidence on the pharmacological value of this approach. In an experimental silicosis model, intratracheal delivery of chitosan–quercetin nanoparticles produced markedly superior anti-fibrotic, antioxidant, and anti-inflammatory outcomes compared to the free flavonoid, including significant reductions in ROS, MDA, IL-1β, and TNF-α, alongside improved lung histology and reduced extracellular matrix deposition [250]. Critically, this enhancement was achieved with minimal systemic toxicity, underscoring the tissue-targeting potential of the nanocarrier platform. The authors further demonstrated downregulation of α-smooth muscle actin, a key fibrosis marker, suggesting that nanocarrier-assisted quercetin delivery holds genuine promise for pulmonary fibrotic diseases such as silicosis and, by extension, other chronic inflammatory lung pathologies.

A conceptually distinct and innovative nanoparticle design was reported by Zhang et al. (2024), who synthesized D-mannitol–cerium–quercetin/rutin coordination polymer nanoparticles (MCQ/R NPs). This metal–organic framework-like architecture exploits the chelating properties of flavonoids to form well-defined nanostructures with intrinsic antioxidant and anti-inflammatory dual functionality. In an LPS-induced acute lung injury model, MCQ/R NP administration produced superior attenuation of pulmonary tissue inflammation relative to conventional quercetin or rutin formulations [272], demonstrating that nanoparticle architecture can amplify, rather than merely preserve, the biological activity of the encapsulated phytochemical.

Beyond quercetin, kaempferol nanoparticle formulations have shown compelling neuroprotective outcomes. Intraperitoneal administration of kaempferol nanoparticles in models of postoperative neurocognitive disorder significantly decreased hippocampal neuroinflammation markers (TNF-α, IL-6, IL-1β), reduced microglial activation, and improved cognitive performance [226], outcomes that were not consistently reproduced by the free flavonoid at equivalent doses. These findings reinforce the concept that nano-formulation can expand the therapeutic application of flavonoids by enabling concentrations at target tissues that are pharmacologically relevant but not achievable through conventional supplementation.

### 7.2. Solid Lipid Nanoparticles

Solid lipid nanoparticles (SLNs) represent a second-generation lipid-based carrier that offers the biocompatibility and lipophilic drug solubilization of emulsions while providing a solid matrix that confers physical stability and prolonged release. Because the blood–brain barrier (BBB) constitutes an additional pharmacokinetic barrier beyond gastrointestinal absorption, SLNs are of particular relevance for neurological applications.

Kaempferol-loaded SLNs (K-SLNs) administered orally in a focal cerebral ischemia model achieved meaningful concentrations in the central nervous system, leading to decreased infarct volume, reduced ROS generation, suppression of NF-κB and phosphorylated STAT3 signaling, and enhanced myelination and neurological recovery [225]. The authors explicitly attributed the superior efficacy to the surface potential of the SLNs, which facilitated BBB transcytosis—a mechanism unavailable to the free flavonoid. This example is instructive for the field because it demonstrates that the choice of carrier architecture is not merely a formulation convenience but a mechanistic determinant of central nervous system target engagement and, ultimately, therapeutic outcome.

### 7.3. Liposomal Encapsulation

Liposomes—phospholipid bilayer vesicles that can accommodate both hydrophilic and lipophilic cargo—are among the most clinically advanced drug delivery platforms, with multiple liposomal drug products approved for human use. Their capacity to encapsulate flavonoids within a biomimetic membrane structure protects the compound from oxidative degradation, promotes cellular uptake via membrane fusion, and can be functionalized with targeting ligands for organ-specific delivery [345].

Quercetin-loaded liposomes have been characterized as stable formulations with enhanced anti-inflammatory activity in the context of hepatic ischemia-reperfusion injury, in which they reduced inflammatory markers and improved tissue recovery relative to free quercetin [161]. In a sepsis model induced by LPS challenge, liposomal encapsulation potentiated quercetin’s inhibitory effects on pulmonary NF-κB-dependent cytokine production and NLRP3 inflammasome activation and was associated with a significant reduction in septic mortality in mice [273]. These findings are noteworthy not only pharmacologically but also as proof-of-concept that liposomal delivery can rescue the therapeutic activity of a flavonoid in a high-inflammatory milieu in which the free compound may be rapidly inactivated or insufficiently absorbed.

The specificity of liposomal delivery can be further enhanced by surface modification with targeting ligands. Wei et al. (2021) demonstrated this principle by coating quercetin-loaded liposomes with galactosylated chitosan to exploit the asialoglycoprotein receptor (ASGPR), which is highly expressed on hepatocytes. In an acute liver injury model induced by D-galactosamine/LPS, these hepatocyte-targeted liposomes promoted M2 macrophage polarization, inhibited liver enzyme release, reduced lipid oxidation, and enhanced antioxidant activity [173]—outcomes consistent with active organ targeting rather than passive tissue distribution. This study exemplifies the next generation of liposomal design for nutraceutical applications: moving from organ-level accumulation toward cell-type-selective delivery.

### 7.4. Phytosomes (Phospholipid Complexes)

Phytosomes are molecular complexes formed by the non-covalent interaction between a phytochemical—typically a polyphenol—and phosphatidylcholine, the principal phospholipid of cell membranes. This complexation confers amphiphilic character on an otherwise poorly soluble molecule, dramatically increasing its oral bioavailability by facilitating passage through intestinal epithelial membranes. Phytosomes are generally recognized as safe and require no organic solvents in their final form, which makes them among the most accessible advanced delivery platforms for nutraceutical commercialization [346].

The clinical relevance of phytosome technology for flavonoids is directly supported by data included in this review. In a randomized, open-label controlled clinical trial of 100 patients with mild-to-moderate COVID-19, participants receiving standard treatment supplemented with Quercetin Phytosome^®^ (500 mg/day) demonstrated faster viral clearance (34 vs. 12 patients testing negative after one week) and earlier symptom resolution compared to standard treatment alone, alongside a significant reduction in lactate dehydrogenase (LDH), a biomarker of systemic inflammation [321]. The formulation was well tolerated with no adverse effects reported. These results provide direct human evidence that phytosome encapsulation can translate the bioavailability limitations of quercetin into clinically measurable therapeutic benefit—a crucial distinction from the predominantly preclinical evidence base for other delivery platforms.

The bioavailability enhancement achieved by phospholipid complexation has been quantified in pharmacokinetic studies external to this review: the phytosome form of quercetin has been reported to increase relative oral bioavailability about 20-fold higher than that attained with crystalline quercetin in some formulations [347]. This magnitude of improvement has profound implications for dose selection in clinical trials and for the interpretation of preclinical dose-efficacy relationships.

### 7.5. Emerging and Complementary Platforms: Nanoemulsions, Cyclodextrins, and Structural Modifications

Beyond the four platforms described above, several additional strategies merit consideration in any comprehensive discussion of flavonoid delivery. Nanoemulsions—thermodynamically stable, oil-in-water dispersions with droplet sizes below 200 nm—improve the solubility of lipophilic flavonoids without the need for solid-state encapsulation and have shown particular promise for dermal and pulmonary administration routes [348]. Cyclodextrin inclusion complexes, in which the hydrophobic flavonoid is accommodated within the hydrophobic cyclodextrin cavity, improve aqueous solubility and bioavailability through a host–guest complexation mechanism that requires no lipid excipients and is compatible with oral, parenteral, and inhalation delivery [349].

Structural modification represents a complementary approach that operates at the molecular rather than formulation level. Glycosylation, methylation, and acylation of the flavonoid scaffold can modulate membrane permeability, metabolic stability, and target selectivity without requiring a carrier system [35]. The flavonoid glycosides rutin and hyperoxide, for instance, have markedly different pharmacokinetic profiles from their respective aglycones (quercetin and quercetin-3-glucuronide), and the interconversion of these forms by the gut microbiota represents a poorly characterized but potentially influential variable in the clinical pharmacology of dietary flavonoids.

Finally, the integration of targeted delivery with controlled-release kinetics—achievable through stimuli-responsive nanoparticles that release their cargoes in response to pH, redox potential, or enzymatic activity characteristic of inflamed tissue—represents a frontier that has begun to be explored for quercetin in hepatic disease models [152] As highlighted by Feng et al. (2025) with quercetin–iron nanoparticles in acute liver failure, the convergence of antioxidant nanomaterial chemistry and flavonoid pharmacology may yield hybrid therapeutic entities whose mechanisms of action are qualitatively distinct from either component alone [174] Therefore, the evidence reviewed (Table 16) collectively demonstrates that nano- and micro-encapsulation technologies are not merely incremental refinements but are transformative strategies capable of qualitatively altering the pharmacological profile of flavonoid nutraceuticals.

Inflammatory diseases represent a major global public health challenge, affecting populations across all geographic regions [350]. Chronic non-communicable diseases associated with persistent inflammation, such as cardiovascular diseases, diabetes mellitus, chronic respiratory diseases, neurodegenerative disorders, liver diseases, autoimmune conditions, and cancer, account for a large proportion of global morbidity and mortality. These diseases share common pathophysiological mechanisms, such as chronic inflammation and oxidative stress, but their prevalence and incidence depend on regional characteristics such as environmental exposure, dietary habits, socioeconomic factors, access to healthcare, aging population and genetic background [351].

Chronic respiratory diseases are common in populations with high exposure to air pollution and tobacco use. The prevalence of metabolic and liver diseases is increasing in Westernized and fast-urbanizing populations. Neurodegenerative diseases are also becoming more common worldwide due to population aging, especially in high- and middle-income countries. Although regional differences exist, inflammatory signaling pathways including reactive oxygen species (ROS), NF-κB activation, MAPKs, inflammasome signaling, and dysregulation of cytokines are consistently implicated in these pathological conditions [352].

## 8. Safety, Toxicology, and Regulatory Aspects of Flavonoid-Based Nutraceuticals

The therapeutic development of flavonoids as nutraceuticals and candidate pharmaceutical agents requires that their efficacy be accompanied by a rigorous characterization of their safety, dose-dependent toxicological profile, and compliance with existing regulatory frameworks. While the preclinical and clinical evidence reviewed in the preceding sections consistently indicates a favorable tolerability profile for quercetin, kaempferol, luteolin, apigenin, and epicatechin, a number of important caveats—encompassing high-dose toxicity, pharmacokinetic herb–drug interactions, vulnerable populations, and regulatory status across major jurisdictions—must be explicitly addressed to allow a balanced evaluation of their translational potential.

### 8.1. General Safety Profile and Observed Adverse Effects in Human Studies

At dietary intake levels, which for most Western populations range from 20 to 500 mg total flavonoids per day depending on food habits, the five flavonoids reviewed here are universally regarded as safe. This conclusion is supported by decades of epidemiological data on flavonoid-rich diets and by the absence of serious adverse events in the clinical trials summarized in Section 6 of this review. The multi-target, partial-inhibition mechanism of action whereby flavonoids modulate rather than abolish endogenous inflammatory and redox pathways may itself confer a mechanistic basis for this favorable safety window, since complete pathway blockade, which is the typical source of dose-limiting toxicity for conventional drugs, is not their mode of action.

At supplemental doses used in clinical trials (typically 100–2000 mg/day), the adverse event profile across the reviewed compounds is characterized by mild and generally transient gastrointestinal effects. The most systematically documented evidence comes from quercetin. Han et al. (2020) conducted a randomized, double-blind, placebo-controlled dose-escalation study in COPD patients using quercetin at 500, 1000, and 2000 mg/day for one week; no serious adverse events were recorded, and only mild gastroesophageal reflux—present in both quercetin and placebo groups—was noted. This finding directly underpins that a 2 g/day quercetin dose was administered to patients without side effects [316] and is important for framing dose selection in future clinical trials. For luteolin, a 6-month randomized trial of a chlorogenic acid–luteolin combination reported no notable adverse effects [324], and an 8-week citrus flavone combination study in healthy volunteers likewise recorded no significant adverse events [325]. For apigenin, a 38-week chamomile extract trial (500 mg × 3 daily) reported no serious adverse events [328]. Epicatechin at 75–150 mg/day for 24 weeks was well tolerated in a pediatric Friedreich’s ataxia cohort [329].

Two important caveats to this generally reassuring picture must be stated. First, most clinical trials cited in this review are of short-to-medium duration (4–38 weeks) and involve modest sample sizes. Long-term safety data at pharmacological doses (i.e., ≥500 mg/day) for all five flavonoids remain limited, and formal no observed adverse effect levels and acceptable daily intakes have not been established by regulatory agencies for isolated flavonoid supplements in most jurisdictions. Second, the possibility of prooxidant activity at high concentrations—observed in vitro for quercetin and kaempferol under specific conditions—and the dose-dependent paradoxical hepatotoxicity reported by Tian et al. (2021) for high-dose kaempferol (20–40 mg/kg i.v.) in an acute liver failure model [175] are experimental signals that, while not directly replicated in human studies, preclude categorical claims of absolute safety at all doses and routes.

### 8.2. Pharmacokinetic Herb–Drug Interactions

Flavonoids are substrates and, at pharmacological concentrations, inhibitors of multiple cytochrome P450 (CYP) isoenzymes and drug transporter proteins, making herb–drug pharmacokinetic interactions a clinically relevant safety concern, particularly for patients with inflammatory diseases who frequently receive polypharmacy. The same CYP isoenzymes that metabolize flavonoids (principally CYP1A1, CYP1A2, CYP2C9, CYP2C19, and CYP3A4) are also responsible for the biotransformation of a large proportion of drugs in clinical use [3]. Inhibition of these enzymes by flavonoids can reduce drug clearance, elevate plasma drug concentrations, and precipitate toxicity.

Quercetin is the most extensively studied compound in this regard and presents the most clinically significant interaction profile. It is an inhibitor of CYP3A4, CYP1A2, and P-glycoprotein, and has been shown in pharmacokinetic studies to increase the plasma area under the curve of co-administered cyclosporine, digoxin, and paclitaxel [3]. Relevant to the patient populations discussed in this review, quercetin may potentiate the anticoagulant effect of warfarin by displacing it from albumin binding sites and inhibiting its CYP2C9-mediated metabolism—a combination requiring clinical caution in patients with liver fibrosis or inflammatory conditions receiving anticoagulant therapy [33,45]. Apigenin inhibits CYP1A2, CYP2C9, and CYP2C19, raising the potential for elevated exposure to warfarin, phenytoin, and proton pump inhibitors. Its GABA-A receptor agonism creates an additive sedation risk with benzodiazepines and alcohol that is of particular relevance for patients with anxiety or neuroinflammatory disorders, both populations discussed in this review [56,327]. Epicatechin and green tea catechins inhibit intestinal CYP3A4 and organic anion-transporting polypeptides (OATP1B1/1B3), with documented pharmacokinetic interactions with rosuvastatin and nadolol. The latter interaction—relevant for cardiovascular comorbidities common in patients with the diseases reviewed here—can substantially reduce nadolol bioavailability [64,65,66]. Although these interactions are largely characterized in vitro or in single-dose pharmacokinetic studies, their clinical relevance at the supplemental doses used in the reviewed trials merits prospective monitoring in future clinical studies.

It is worth noting that the direction of interaction is not uniformly adverse: co-administration of quercetin nanoparticles with certain chemotherapeutic agents has been explored as a strategy to sensitize tumor cells, and flavonoid-mediated CYP inhibition has been proposed as a pharmacological tool to improve oral bioavailability of co-administered drugs with first-pass metabolism [3,33]. However, these potential benefits do not negate the need for interaction screening in clinical trial protocols.

### 8.3. Considerations for Vulnerable Populations

Several population subgroups warrant specific safety consideration when flavonoid supplementation is contemplated at pharmacological doses.

Pregnancy and lactation. Dietary flavonoid intake from food is not contraindicated in pregnancy. However, high-dose supplementation raises concerns for several reasons. Quercetin and kaempferol have demonstrated weak phytoestrogenic activity and may interfere with estrogen metabolism via CYP1A2 inhibition, potentially affecting gestational hormonal homeostasis. Apigenin has demonstrated uterotonic effects in rodent models at supra-dietary doses. The absence of clinical trial data in pregnant women means that regulatory agencies universally recommend against pharmacological-dose flavonoid supplementation during pregnancy and lactation until controlled safety data are available [45,56,63,353].

Pediatric populations. The epicatechin trial by Qureshi et al. (2020) in pediatric Friedreich’s ataxia patients (75–150 mg/day) represents one of the few prospective safety assessments in children and demonstrated acceptable tolerability [329]. The luteolin study in children with autism spectrum disorder by Taliou et al. (2013) likewise reported good tolerability [323]. However, allometric dose scaling, developmental differences in CYP enzyme expression, and the long-time horizons involved in pediatric nutritional interventions warrant dedicated pediatric safety studies before broad clinical application.

Hepatic impairment. Several flavonoids, most notably concentrated green tea catechins (epicatechin-containing), have been associated with drug-induced liver injury in case reports and systematic reviews, primarily at very high doses from concentrated supplements [117]. While this review focuses on epicatechin in the context of protective hepatic effects—which are extensively documented preclinically—the hepatotoxicity signals from high-dose catechin supplements underscore the importance of distinguishing between dietary consumption and concentrated supplement administration [67]. The European Commission to the European Food Safety Authority (EFSA) 2018 Scientific Opinion specifically identified >800 mg/day total catechins from supplements as a hepatotoxicity risk [354]. Patients with pre-existing liver disease should therefore use concentrated epicatechin or catechin supplements with medical supervision.

### 8.4. Genotoxicity and Promutagenic Considerations

Quercetin was evaluated by the International Agency for Research on Cancer (IARC) in 1983 on the basis of in vitro mutagenicity in the Ames assay and early rodent carcinogenicity data [355]. This evaluation has been substantially revised in subsequent decades as the mechanisms of apparent genotoxicity have been better characterized. The mutagenic activity of quercetin in bacterial systems is dependent on metabolic activation conditions that do not replicate human physiology, and long-term animal studies at high doses have not confirmed carcinogenicity in a regulatory context [3]. A critical appraisal of the available genotoxicity and carcinogenicity data for quercetin concluded that its in vitro mutagenicity does not translate to in vivo genotoxic risk, and that quercetin is safe at doses consistent with food supplement use [356]. Nevertheless, genotoxicity assessment remains a standard regulatory requirement for high-dose isolated flavonoid preparations, and investigators submitting marketing authorization applications or novel food notifications in the European Union or the United States should include an ICH-compliant genotoxicity battery.

### 8.5. Regulatory Status Across Major Jurisdictions

The regulatory classification of flavonoid preparations varies substantially across jurisdictions, reflecting the inherent ambiguity between foods, food supplements, and medicinal products for this compound class. In the United States, the five flavonoids reviewed here are regulated as dietary supplements under the Dietary Supplement Health and Education Act of 1994, which does not require pre-market demonstration of safety or efficacy [357]. Quercetin holds GRAS status, and epicatechin is consumed as part of recognized food sources. The FDA has nonetheless issued consumer advisories on concentrated green tea extract supplements due to hepatotoxicity signals [358] and requires that structure-function label claims carry a disclaimer of non-evaluation by the agency [359].

In the European Union (EU), food supplements are governed by Directive 2002/46/EC [360], and isolated flavonoid preparations above threshold concentrations may require pre-market authorization under the Novel Food Regulation 2015/2283 [361]. The most relevant regulatory assessments are two EFSA Scientific Opinions: the 2018 opinion on green tea catechins identified hepatotoxicity as a potential risk above 800 mg/day [354], and a critical appraisal of quercetin safety data concluded that quercetin at doses relevant to supplement use does not represent a confirmed genotoxic risk in vivo [356], a position broadly reflected in EFSA’s labelling guidance for EU member states.

In Brazil, food supplements are regulated under RDC n° 243/2018, which establishes the general sanitary requirements for this product category, and Instructor Normative (IN) n° 28/2018, which defines the positive lists of permitted constituents—including bioactive substances such as quercetin—along with minimum and maximum daily intake limits by population group [362,363]. Quercetin is a bioactive substance with a defined maximum daily dose of 280 mg for adults. Health claims associated with supplement constituents must be substantiated and listed in the IN n° 28/2018 positive list. Brazilian clinical trials involving flavonoid supplements must be registered on the Brazilian platform for assessment and ethical approval requests called “Plataforma Brazil”, and ANVISA notification is required when the intervention constitutes a regulated supplement product.

Across all major jurisdictions, a consistent pattern is: flavonoids consumed at dietary levels are universally regarded as safe and require no specific regulatory action, whereas isolated, high-dose supplement preparations occupy a more complex regulatory space that demands varying degrees of pre-market safety substantiation, constituent authorization, and labelling compliance.

## 9. Conclusions

In terms of the mechanisms of action, flavonoids act on multiple targets. On one side, all selected flavonoids present structures that have intrinsic antioxidant activity because, for instance, they can donate hydrogen from the hydroxyl groups and accept electrons that are stabilized within the benzene rings. In addition to this mechanism, the selected flavonoids can inhibit the activation of intracellular signaling pathways (e.g., MAPK) and transcription factors (e.g., NF-κB) as well as induce the activation of the transcription factor Nrf2 that upregulates the expression of antioxidant endogenous molecules (Figure 7). These are the main mechanisms of the top five most studied flavonoids selected for the present narrative review articles, as discussed in detail above. These mechanisms explain how flavonoids and flavonoid-rich foods exhibit significant antioxidant and anti-inflammatory activity in various experimental models. There are also disease/context specific mechanisms of the flavonoids as discussed. It is possible that this multi-target mechanism of flavonoids is responsible for the relative absence of major side effects as mentioned in the clinical trials cited above, because by not abolishing one mechanism, but rather reducing multiple targets, flavonoids do not achieve full inhibition of endogenous pathways that may also present physiological roles. At the same time, the multi-target mechanisms may also be the explanation for the adaptive bioactivity of flavonoids since these molecules can inhibit varied disease context mechanisms.

Finally, as we discussed above, there is a scarcity of well-controlled clinical trials with high numbers of patients testing flavonoids. This is a contrast with the large number of studies on flavonoids, which reached 16,055 published articles in 2025. In this sense, it seems that the major gap to finally translate these pre-clinical studies on the biological activities and nutraceutical properties of flavonoids into clinical use depends on standardized human intervention studies, studies on metabolic interactions, and the development of formulations that enhance bioavailability and organ-targeted administration forms to achieve the maximal potential of these compounds.

## Figures and Tables

**Table 3:** Summary of the literature on selected flavonoids against doxorubicin-induced liver damage from 2020 to 2025.

Doxorubicin-induced liver toxicity				
AUTHORS	TREATMENTS	SUBJECTS	MODEL INDUCTION	MAIN FINDINGS
Ahmed et al. (2022) [147]	• Quercetin [50 mg/kg] • Oral administration • Every other day for 5 weeks	• Male Wistar rats [120–145 g] • 10 weeks old	• Doxorubicin [2 mg/kg] • Intraperitoneal administration • Two times a week for 5 successive weeks	Quercetin effects compared to doxorubicin group: • ↓ Serum MDA, AST, ALT, and ALP activities, albumin and total bilirubin levels • ↓ Histological liver alterations, AFP, and CA19.9 serum levels • ↑ SOD, GSH, GPx, GST, and Nrf2 expression • ↓ Liver p53 and TNF-α expression
Owumi et al. (2021) [146]	• Luteolin [100 mg/kg] • Oral administration • For 14 consecutive days	• Male Wistar rats [160 ± 5 g]	• Doxorubicin [2 mg/kg] • Intraperitoneal administration • Every other day for 6 days	Luteolin effects compared to doxorubicin group: • ↓ ALT, AST, ALP, urea, creatinine, LDH, and γGT • ↓ ROS, MDA, IL-1β, TNF-α, caspases-3 and caspase-9 • ↑ SOD, CAT, GSH, and IL-10

Abbreviations: AFP (alpha-fetoprotein), AST (aspartate aminotransferase), ALP (alkaline phosphatase), ALT (alanine aminotransferase), CA 19.9 (an antigen released by pancreatic cancer cells), CAT (catalase), GPx (glutathione peroxidase), γGT (gamma-glutamyl transferase), GST (glutathione S-transferase), GSH (reduces glutathione), IL-1β (interleukin-1 beta), IL-10 (interleukin-10), LDH (lactate dehydrogenase), MDA (malondialdehyde), Nrf2 (nuclear factor erythroid 2-related factor 2), ROS (reactive oxygen species), SOD (superoxide dismutase), TNF-α (tumor necrosis factor-alpha), ↑ (increase), ↓ (decrease).

**Table 5:** Summary of the literature on selected flavonoids against liver ischemia-reperfusion injury, from 2020 to 2025.

Hepatic ischemia-reperfusion injury				
AUTHORS	TREATMENTS	SUBJECTS	MODEL INDUCTION	MAIN FINDINGS
Lin et al. (2025) [159]	• Quercetin [50 mg/kg] • Intraperitoneal administration	• Wild-type male C57BL/6J mice	• 90 min of hepatic ischemia followed by reperfusion • Samples obtained 6 h later	Quercetin effects compared to stimulus group: • ↓ ALT, AST, MDA, and LDH • ↓ F4/80, MPO • ↑ CAT • ↓ IL-1β and TNF-α • ↓ Caspase-8/ASC binding • ↓ NLRP3 and AIM2 inflammasome formation
Ferreira-Silva et al. (2022) [160]	• Quercetin liposomal formulation [1.3 mg/kg] • Intravenous administration 24 h before the surgical procedure	• Male Wistar Han rats • 56–62 days old	• 20 min of hepatic ischemia followed by reperfusion • Blood samples obtained 6 and 24 h later	Quercetin liposomal formulation effects compared to stimulus group: • ↓ Levels of ALT, ALP, and γGT • ↓ TNF-α
Chen et al. (2022) [161]	• Kaempferol pretreatment [30 or 60 mg/kg] • Oral administration • For 7 days	• Male C57BL/6 mice [18–22 g] • 8–10 weeks old	• 60 min of hepatic ischemia followed by reperfusion • Day 8 • Samples obtained 6 h later	Kaempferol effects compared to stimulus group: • ↓ ALT, AST, and MDA • ↑ SOD and GSH • ↓ TNF-α, IL-6, p-NF-κB/p65 and Bax • ↑ IL-10, Bcl-2, Nrf2, and HO-1

Abbreviations: AIM2 (absent in melanoma 2, a member of inflammasome), ALP (alkaline phosphatase), ALT (alanine aminotransferase), ASC (apoptosis-associated speck-like protein containing a CARD), AST (aspartate aminotransferase), Bax (Bcl-2 Associated X protein), Bcl-2 (B-cell lymphoma 2), CAT (catalase), F4/80 (cell surface glycoprotein marker for mature mouse macrophages), γGT (gamma-glutamyl transferase), GSH (reduces glutathione), HO-1 (heme-oxygenase-1), IL-1β (interleukin-1 beta), IL-6 (interleukin-6), IL-10 (interleukin-10), LDH (lactate dehydrogenase), MDA (malondialdehyde), MPO (myeloperoxidase), NLRP3 (NOD-, LRR- and pyrin domain-containing protein 3), p-NF-κB/p65 (phosphorylated nuclear factor kappa-light-chain-enhancer of activated B cells p65 subunit), Nrf2 (Nuclear factor erythroid 2-related factor 2), SOD (superoxide dismutase), TNF-α (tumor necrosis factor-alpha).

**Table 13:** Summary of the literature on selected flavonoids against allergic airway inflammation induced by ovalbumin, from 2020 to 2025.

Allergic airway inflammation (Asthma model)				
AUTHORS	TREATMENTS	SUBJECTS	MODEL INDUCTION	MAIN FINDINGS
Quan et al. (2024) [297]	• Luteolin [10 and 20 mg/kg]	• Male C57BL/6 [20–25 g] • 6 weeks old	• Ovalbumin [100 µg and nebulized 0,2%] • Intraperitoneal administration (0,7 and 14 day) • Ultrasonic nebulizer (21 to 28 day)	Luteolin effects compared to stimulus group: • ↓ TSLP, IL-33, and IL-25 • ↓TGF-β1, MMP-9, and α-SMA expression levels
Fang et al. (2023) [298]	• Quercetin [30 mg/kg] • Oral administration • For 7 days	• BALB/c mice • 6 weeks old	• Ovalbumin [dose and route of administration not specified]	Quercetin effects compared to stimulus group: • ↓ Periostin in bronchoalveolar lavage • ↓ Airway inflammation, fibrosis, and bronchial hyperreactivity • ↓ TGF-β1/Smad
Rajizadeh et al. (2023) [299]	• Quercetin [50 mg/kg] • Intraperitoneal administration • For 7 days	• Male Wistar rats [200–250 g] • 8 weeks old	• Ovalbumin [1 mg for sensibilization and 1 g for inhalation challenge] • Intraperitoneal administration for sensitization [Day 0 and day 7] • Inhalation for challenge [For 28 days]	Quercetin effects compared to stimulus group: • ↓ MDA • ↑ SOD, GPx, and CAT • ↓ TNF-α and IL-6 • ↑ IL-10 • ↓ Expression of GATA-3, α-SMA, IL-1β, TNF-α, and TGF-β • ↑ Expression of T-bet • ↓ Inflammation
Molitorisova et al. (2021) [300]	• Kaempferol [20 mg/kg]	• Male Dunkin-Hartley guinea pigs [200–350 g]	• Ovalbumin [5mg] • Intraperitoneal administration (1st and 4th day) • Subcutaneous administration (1st, 9th day) • Inhalation (12th, 15th, 18th and 20th day) • For 21 days	Kaempferol effects compared to stimulus group: • ↓ TGF-β1, IL-5, IL-13 • ↓ Eosinophils • ↓ Cough reflex
Wu et al. (2025) [301]	• Kaempferol [10, 20 and 40 mg/kg] • Oral administration	• Female BALB/c mice [18–20 g] • 4 weeks old	• Ovalbumin [100 µg and nebulized 5%] •Intraperitoneal administration (0,7 and 14 day) • Ultrasonic nebulizer (21 to 35 day)	Kaempferol effects compared to stimulus group: • ↓ IgE, TNF-α, Histamine, IL-1β, IL-6, and IL-8 • ↓ NOTCH1, NOTCH2, and NOTCH3 • ↓ TLR4 and NLRP3
Wang et al. (2021) [302]	• Luteolin [10 and 20 mg/kg] • Gavage administration	• Female balb/c mice [20–25 g] • 6 weeks old	• Ovalbumin [20 µg and nebulized 2%] • Intraperitoneal administration (0,7 and 14 day) • Ultrasonic nebulizer (22 to 29 day)	Luteolin effects compared to stimulus group: • ↓ Lymphocytes and eosinophils • ↓ IgE levels • ↓ Staining of PAS and Masson • ↑ PI3K p85 and p-mTOR levels
Yu et al. (2023) [303]	• Apigenin [10 and 20 mg/kg] • Oral administration	• Male SPF C57BL/6 J mice [18–22g] • 6 weeks old	• Ovalbumin [20 µg and nebulized 3%] • Intraperitoneal administration (0,7 and 14 day) • Ultrasonic nebulizer (21 to 62 day)	Apigenin effects compared to stimulus group: • ↓ Total leukocytes, neutrophils, eosinophils and lymphocytes • ↓ Peribronchial collagen deposition • ↓ mRNA expression of IL-5, IL-4, IL-13, IL-17, TNF-α, and IFN-γ • ↓ ASK1, JNK, and p38

Abbreviations: α-SMA (alpha-smooth muscle actin), ASK1 (apoptosis signal-regulating kinase 1), IFN-γ (interferon gamma), IgE (immunoglobulin E), IL-13 (interleukin-13), IL-17 (interleukin-17), IL-1β (interleukin-1β), IL-25 (interleukin-25), IL-33 (interleukin-33), IL-4 (interleukin-4), IL-5 (interleukin-15), IL-6 (interleukin-6), IL-8 (interleukin-8), JNK (c-Jun N-terminal kinases), MMP-9 (matrix metalloproteinase-9), NLRP3 (NOD-, LRR- and pyrin domain-containing protein 3), NOTCH 1 (Neurogenic locus notch homolog protein 1), NOTCH 2 (Neurogenic locus notch homolog protein 2), NOTCH 3 (Neurogenic locus notch homolog protein 3), p-mTOR (phosphorylated mechanistic target of rapamycin), p38 (p38 mitogen-activated protein kinase), PAS (Periodic Acid–Schiff histological staining), PI3K p85 (phosphoinositide 3-kinase subunit p85), TGF-β1 (transforming growth factor beta-1), TLR4 (toll-like receptor 4), TNF-α (tumor necrosis factor-alpha), TSLP (thymic stromal lymphopoietin), ↑ (increase), ↓ (decrease).

**Table 15:** Clinical trials on epicatechin intake in the treatment of inflammatory conditions related to selected systems (liver, nervous system, lung), from 2020 to 2025.

Epicatechin intake in the treatment of inflammatory conditions				
AUTHORS	TREATMENTS	STUDY PHASE AND PARTICIPANTS	DISEASE	MAIN FINDINGS
Qureshi et al. (2020) [329]	• Epicatechin [75–150 mg/day] • For 24 weeks	• Phase II, open-label, baseline-controlled single-center trial • Trial is registered at ClinicalTrials.gov as NCT02660112 • 10 participants, ages 10 to 22, with confirmed Friedreich’s ataxia	• Friedreich’s ataxia	Epicatechin effects: • ↓ Systolic blood and diastolic blood pressure • Stronger in hypertensive individuals • ↑ Endothelial function and bioavailability of NO • ↓ Oxidative stress and inflammation

Abbreviation: NO (nitric oxide).

**Table 16:** Summary of advanced delivery systems studied for flavonoids reviewed in this article, with key pharmacological advantages and supporting experimental evidence.

Main flavonoid-based delivery systems			
DELIVERY SYSTEM	REPRESENTATIVE FLAVONOID(S)	KEY ADVANTAGE(S)	SUPPORTING EVIDENCE (SELECTED)
Polymeric nanoparticles (chitosan) [249]	Quercetin	Lung targeting; ↑ anti-fibrotic efficacy; minimal systemic toxicity	Chitosan–quercetin nanoparticles in silicosis model: ↓ ROS, MDA, IL-1β, TNF-α; improved histology
Coordination polymer nanoparticles [271]	Quercetin, Rutin	Multi-drug platform; ROS scavenging; inflammatory action	Polymer nanoparticles in LPS-induced acute lung injury: ↓ lung inflammation vs. conventional treatment
Kaempferol nanoparticles [225]	Kaempferol	Brain-targeted delivery; ↓ neuroinflammation; ↑ cognitive recovery	Kaempferol nanoparticles effects: ↓ TNF-α, IL-6, IL-1β; ↓ microglial activation; ↑ postoperative cognitive performance
Solid lipid nanoparticles [224]	Kaempferol	Blood–brain barrier penetration; sustained release; enhanced central nervous system bioavailability	Oral kaempferol solid lipid nanoparticles in focal cerebral ischemia: ↓ infarct volume, ↓ ROS, ↓ NF-κB/p-STAT3, ↑ myelination
Liposomes [160,272]	Quercetin	Aqueous compatibility; ↑ encapsulation efficiency; organ-level targeting	Quercetin liposomes in sepsis: ↑ inhibition of NF-κB cytokines; ↓ inflammasome activation; ↓ mortality [272] Quercetin liposomes in hepatic ischemia/reperfusion injury: ↓ inflammatory markers; ↑ tissue recovery [160]
Galactosylated chitosan-modified liposomes [173]	Quercetin	Active hepatocyte targeting via asialoglycoprotein receptor; macrophage polarization	Quercetin liposomes in D-galactosamine /LPS model: M2 macrophage polarization; ↓ liver enzymes; ↓ lipid oxidation; ↑ antioxidant activity
Phytosome (phospholipid complex) [320]	Quercetin	Modulation of the host’s hyperinflammatory response; GRAS status	Randomized controlled trial (COVID-19): faster viral clearance (34 vs. 12 negative), earlier symptom resolution; ↓ LDH; well tolerated
Quercetin-loaded nanoparticle [152]	Quercetin	Superior hepatoprotection; ↑ bioavailability; targeted delivery vs. free form	Quercetin nanoparticles in acrylamide-induced hepatotoxicity: MAPK/NF-κB/NLRP3 signaling pathways modulation
Quercetin-Fe nanoparticles [174]	Quercetin	Dual antioxidant–anti-inflammatory action; anti-senescence; ROS neutralization	Quercetin-Fe nanoparticles in acute liver failure: blocked macrophage inflammatory activation; ↓ apoptosis; ↑ liver regeneration

Abbreviations: ROS (reactive oxygen species), MDA (malondialdehyde), NF-κB (nuclear factor kappa B), IL (interleukin), TNF-α (tumor necrosis factor alpha), LPS (lipopolysaccharide), GRAS (generally recognized as safe), and LDH (lactate dehydrogenase), ↑ (increase), ↓ (decrease).

## Data Availability

No new data were created or analyzed in this study. Data sharing is not applicable to this article.

## Abbreviations

4CL	4-coumarate CoA ligase
AA	Arachidonic acid
ABTS	2,2′-azino-bis(3-ethylbenzothiazoline-6-sulfonic acid)
ACE	Angiotensin-converting enzyme
ACEI	Inhibitory angiotensin-converting enzyme
AChE	Acetylcholinesterase
AFP	Alpha fetoprotein
AIM2	Absent in melanoma 2
AKI	Acute kidney injury
ALI	Acute lung injury
ALP	Alkaline phosphatase
ALT	Alanine transaminase
AP-1	Activator protein-1
Arg1	Arginase 1
ASC	Apoptosis-associated speck-like protein containing a CARD
ASM	Airway smooth muscle
AST	Aspartate transaminase
ATF-6	Activating transcription factor-6
Bax	Bcl-2 associated X protein
Bcl-2	B-cell lymphoma 2
BDNF	Brain-derived neurotrophic factor
c-IAP	Cellular inhibitor of apoptosis proteins
C1q	Complement 1q
C3	Complement 3
C4H	Cinnamate 4-hydroxylase
CA19.9	Carbohydrate antigen
CAA	Cellular antioxidant activity
CAT	Catalase
CCI	Chronic construction injury
CCL2	Chemokine ligand 2
CD11b	Cluster of differentiation 11b
CD163	Cluster of differentiation 163
CD206	Cluster of differentiation 206
CD31	Cluster of differentiation 31
CD4	Cluster of differentiation 4
CD86	Cluster of differentiation 86
CDK	Cyclin-dependent kinase
CEA	Carcinoembryonic antigen
CeLutNCs	Cerium-luteolin ion nanocomplexes
CHI	Chalcone isomerase
CHOP	C/EBP homologous protein
CHS	Chalcone synthase
CIRP	Cold-inducible RNA-binding protein
CLP	Cecal ligation and puncture
COPD	Chronic obstructive pulmonary disease
COX	Ciclooxygenases
COX-2	Ciclooxygenase-2
CREB	c-AMP response element-binding protein
CRP	C reactive protein
CSI	Cecal suspension injection
CTSG	Cathepsin G
CXCL10	C-X-C motif chemokine ligand 10
CXCL2	C-X-C motif chemokine ligand 2
CXCR2	C-X-C motif chemokine receptors 2
CYP2A13	Cytochrome P450 family 2 subfamily A member 13
DNA	Deoxyribonucleic acid
DNMT3A	DNA methyltransferase 3a
DPPH	2,2-diphenyl-1-picrylhydrazyl
EGFR	Epicatechindermal growth factor receptor
EMT	Epicatechinthelial-mesenchymal transition
eNOS	Endothelial nitric oxide synthase
ER	Endoplasmatic reticulum
ERK	Extracellular signal-regulated kinases
ETC	Electron transport chains
FBXW7	F-box/WD repeat-containing protein 7
FcεRI	High-affinity IgE receptor
FOXO1A	Forkhead box protein O1
FRAP	Ferric reducing antioxidant power
FTH1	Ferritin pes chain 1
GDBF	Glial cell-derived neurotrophic factor
GFAP	Glial fibrillary acidic protein
GM-CSF	Granulocyte-macrophage colony-stimulating factor
GPR	G protein-coupled receptor
GPx	Glutathione peroxidase
GR	Glutathione reductase
GRK-2	G protein-coupled receptor kinase 2
GRP78	Glucose-regulated protein 78
GSDMD	Gasdermin D
GSH	Glutathione
GSK-3β	Glycogen synthase kinase-3 beta
GST	Glutathione S-transferase
H+	Hydrogen electrons
H_2_O_2_	Hydrogen peroxide
HDAC2	Histone deacetylase 2
HDAC3	Histone deacetylase 3
Hes1	Hairy and enhancer of split 1
HIF1α	Hypoxia-inducible factor 1-alpha
HMGB-1	High mobility group box 1
OH	Hydroxyl
HO-1	Heme oxygenase-1
HO^−^_2_	Hydroperoxyl
HUVEC	Human umbilical vein endothelial cells
Iba-1	Ionized calcium-binding adapter molecule 1
IC50	Half Maximal Inhibitory Concentration
ICAM-1	Intercellular adhesion molecule 1
IDO	Indoleamine-2,3-dioxygenase
IFN	Interferon
IFN-γ	Interferon gamma
IgE	Immunoglobulin E
IKKα	IκB kinase-alpha
IL-10	Interleukin 10
IL-12	Interleukin 12
IL-13	Interleukin 13
IL-18	Interleukin 18
IL-1α	Interleukin 1 alpha
IL-1β	Interleukin-1 beta
IL-4	Interleukin 4
IL-6	Interleukin 6
IL-8	Interleukin 8
iNOS	Inducible nitric oxide synthase
IRE1	Inositol-requiring enzyme-1
IRF	Interferon regulatory factor
IRG1	Immune response gene 1
IκB-α	Nuclear factor of kappa light polypeptide enhancer in B-cells inhibitor alpha
JAK	Janus kinase
JNK	c-Jun amino-terminal kinases
K-SLN	Kaempferol-loaded solid lipid nanoparticles
K3G	Kaempferol 3-O-gentiobioside
Keap1	Kelch-like ECH-associated protein 1
Ki-67	Proliferation marker
L7Gn	luteolin-7-O-glucuronide
LC3	Autophagy-related protein light chain 3
LC3	Microtubule-associated protein 1 light chain 3
LDH	Lactate dehydrogenase
LEF	Lymphoid enhancer-binding factor
LOX	Lipoxygenases
LPO	Lipid peroxidase
LPS	Lipopolysaccharides
MAO	Monoamino oxidase
MAPK	Mitogen-activated protein kinase
MCAO	Middle cerebral artery occlusion
MCL2	Myosin light chain 2
MCP-1	Monocyte chemoattractant protein-1
MCQ/R NP	D-mannitol-cerium-quercetin/rutin coordination polymer nanoparticles
MDA	Malondialdehyde
MG	Mycoplasma gallisepticum
MiR-15a	Micro RNA 15a
MnSOD	Manganese superoxide dismutase
MPO	Myeloperoxidase
mRNA	Messenger ribonucleic acid
MRP	Multi-drug resistance-associated protein
MT-I	Metallothionein-I
MT-II	Metallothionein-II
mtDNA	mitochondrial DNA
mTORC1	Mammalian target of rapamycin complex 1
MUC5AC	Mucin 5AC
MyD88	Myeloid differentiation primary response 88
NADPH	Nicotinamide adenine dinucleotide phosphate
NCOA4	Nuclear receptor coactivator 4
NF-κB	Nuclear factor kappa B
NFATc2	Nuclear factor of activated T-cells cytoplasmic 2
NIK	NF-κB-inducing kinase
NLRP3	NLR family pyrin domain containing 3
NO	Nitric oxide
NOS2	Nitric oxide synthase 2
NOX	NADPH oxidase
NOx	Nitrogen oxides
NQO-1	NAD(P)H-quinone oxidoreductase 1
Nrf2	Nuclear factor erythroid 2-related factor 2
O_2_	Oxygen
O_2_•^−^	Superoxide anion
ONOO	Peroxynitrite
OPS	O-specific polysaccharide
ORAC	Oxygen radical absorbance capacity
OVA	Ovalbumin
p-Akt	Phosphorylated protein kinase B
p-CREB	Phosphorylated cyclic AMP response element-binding protein
p-ERK	Phosphorylated extracellular signal-regulated kinase
p-IKK	Phosphorylated IκB kinase
p-IκB-α	Phosphorylated inhibitor of nuclear factor kappa B
p-JNK	Phosphorylated c-Jun N-terminal kinase
p-NF-κB	Phosphorylated nuclear factor kappa B
p-PI3K	Phosphorylated phosphoinositide 3-kinase
P2X7R	P2X7 receptor
PAL	Phenylalanine ammonia-lyase
PARP1	Poly ADP-ribose polymerase 1
PCO	Protein carbonyl
PERK	Protein kinase R-like ER kinase
PGC-1α	PPARγ coativator-1 alpha
PGE2	Prostaglandin E2
PHD	Prolyl hydroxylase domain
PI3K/Akt	Phosphatidylinositide 3-kinases/protein kinase B
PINK1	PTEN-induced putative kinase 1
PIVKA-II	Protein induced by vitamin K absence or antagonist-II
PKM2	Pyruvate kinase isozyme M2
PND	Postoperative neurocognitive disorder
PPARγ	Peroxisome proliferator-activated receptor gamma
PSD95	Postsynaptic density protein 95
Q3G	Quercetin-3-glucuronide
QFN	Quercetin–iron nanoparticles
RAGE	Receptor for advanced glycation end products
RelB	A subunit of NF-κB called RELB proto-oncogen
RNS	Reactive nitrogen species
ROCK-1	Rho-associated coiled-coil kinase 1
ROS	Reactive oxygen species
RSV	Respiratory syncytial virus
SASP	Senescence-associated secretory phenotype
SDH	Succinate dehydrogenase
Sema4D	Semaphorin 4D
SIRT1	Sirtuin1
SIRT6	Sirtuin 6
SOD	Superoxide dismutase
SphK1	Sphingosine kinase 1
STAT	Signal transducer and activator of transcription
STING	Stimulator of interferon genes
t-CREB	Total Cyclic AMP Response Element-Binding Protein
TBA	Thiobarbituric acid
TBARS	Thiobarbituric acid reactive substances
TCA	Tricarboxylic acid
TCF	T-cell factor
TDO	Tryptophan 2,3-dioxygenase
TGF-β1	Transforming growth factor beta 1
Th2	T helper 2
TLR4	Toll-like receptor 4
TLR7	Toll-like receptor 7
TNF-α	Tumor nuclear factor-alpha
TOMM20	Translocase of outer mitochondrial membrane 20
TRAF	TNF receptor-associated factor
TRAF6	TNF receptor-associated factor 6
Tregs	Regulatory T cells
TRIM25	Tripartite motif-containing protein 25
TrkB	Tropomyosin receptor kinase B
TRPV1	Transient receptor potential vanilloid 1
TSH	Thyroid-stimulating hormone
TSH	Total sulfhydryl
TXNIP	Thioredoxin-interacting protein
UV	Ultraviolet
VCAM-1	Vascular cell adhesion molecule 1
VE-cadherin	Vascular endothelial cadherin
VEGF	Vascular endothelial growth factor
XO	Xanthine oxidase
ZO-1	Zonula occludens-1
α-SMA	Alpha smooth muscle actin
β2-AR	Beta-2 adrenergic receptor
γGT	Gama-glutamyl transferase
γH2AX	DNA double-strand breaks marker
